# Real-World Experiences in Autistic Adult Diagnostic Services and Post-diagnostic Support and Alignment with Services Guidelines: Results from the ASDEU Study

**DOI:** 10.1007/s10803-021-04873-5

**Published:** 2021-01-27

**Authors:** Maria Luisa Scattoni, Martina Micai, Antonio Ciaramella, Tommaso Salvitti, Francesca Fulceri, Laura Maria Fatta, Luise Poustka, Robert Diehm, Georgi Iskrov, Rumen Stefanov, Quentin Guillon, Bernadette Rogé, Anthony Staines, Mary Rose Sweeney, Andrew Martin Boilson, Thora Leósdóttir, Evald Saemundsen, Irma Moilanen, Hanna Ebeling, Anneli Yliherva, Mika Gissler, Tarja Parviainen, Pekka Tani, Rafal Kawa, Astrid Vicente, Célia Rasga, Magdalena Budişteanu, Ian Dale, Carol Povey, Noelia Flores, Cristina Jenaro, Maria Luisa Monroy, Patricia García Primo, Tony Charman, Susanne Cramer, Christine Kloster Warberg, Ricardo Canal-Bedia, Manuel Posada, Diana Schendel

**Affiliations:** 1 Istituto Superiore Di Sanità, Research Coordination and Support Service, Regina Elena 299, 00161 Roma, Italy; 2 Department of Child and Adolescent Psychiatry and Psychotherapy, University Medical Center Göttingen, Gottingen, Germany; 3 Department of Child and Adolescent Psychiatry and Psychotherapy, Medical University of Vienna, Wien, Austria; 4 Institute for Rare Diseases, Plovdiv, Bulgaria; 5 Department of Social Medicine and Public Health, Faculty of Public Health, Medical University of Plovdiv, Plovdiv Town, Bulgaria; 6 Université Toulouse Jean Jaurès, CERPPS, Toulouse, Occitanie France; 7 School of Nursing, Psychotherapy & Community Health, Dublin City University, Dublin, Republic of Ireland; 8 The State Diagnostic and Counselling Centre, 200 Kópavogur, Iceland; 9 Clinic of Child Psychiatry, University and University Hospital of Oulu, Oulu, Finland; 10 Medical Faculty, Oulu University Hospital, Oulu, Finland; 11 University of Oulu, Logopedic Child Language Research Center, Oulu, Finland; 12 Finnish Institute for Health and Welfare, Helsinki, Uusimaa Finland; 13 University of Turku, Research Centre for Child Psychiatry, Turku, Finland; 14 Division of Family Medicine, Department of Neurobiology, Care Sciences and Society, Karolinska Institute, Stockholm, Sweden; 15 Finnish Association for Autism and Asperger’s Syndrome, Helsinki, Uusimaa Finland; 16 Department of Psychiatry, University of Helsinki, Helsinki, Finland; 17 Faculty of Psychology, University of Warsaw, Warsaw, Poland; 18 Center for Biodiversity, Functional and Integrative Genomics, Instituto Nacional de Saúde Doutor Ricardo Jorge, Lisbon, Portugal; 19 Victor Babeş” National Institute for Research and Development in Pathology and Biomedical Sciences, Timisoara, Romania; 20 National Autistic Society, The Centre for Autism, London, UK; 21 Departamento de Personalidad, Evaluación y Tratamiento Psicológicos, INICO—Instituto Universitario de Integración en la Comunidad University of Salamanca, Salamanca, Spain; 22 Departamento de Psicología Evolutiva y de la Educación, INICO—Instituto Universitario de Integración en la Comunidad University of Salamanca, Salamanca, Spain; 23 Instituto de Salud Carlos III, Institute of Rare Diseases Research Madrid, Madrid, Spain; 24 Institute of Psychiatry, Kings College London, London, UK; 25 Department of Public Health, Aarhus University, Aarhus, Denmark; 26 Lundbeck Foundation Initiative for Integrative Psychiatric Research, iPSYCH, Aarhus, Denmark; 27 Department of Economics and Business, Aarhus University, National Centre for Register-Based Research, Aarhus, Denmark

**Keywords:** Autism spectrum disorder, Adults, Diagnosis, Services

## Abstract

Research providing an evidence-base for autistic adult services is sparse. The Autism Spectrum Disorders in the European Union (ASDEU) network implemented an on-line survey to determine gaps in autistic adult diagnostic evaluation and post-diagnostic support services. More than 55% in all groups experienced most of the recommended features for diagnostic evaluation for autistic adults. In contrast, < 2% of adults or carers, and < 21% of professionals experienced each of the recommended features for post-diagnostic support. In contrast to 61% of professionals, only about 30% of autistic adults and carers had knowledge of good local services models for autism diagnosis in adulthood. There are major differences between good practice guidelines for diagnostic and post-diagnostic care for autistic adults, and what is actually experienced by services users and professionals.

**Supplementary Information:**

The online version contains supplementary material available at 10.1007/s10803-021-04873-5.

Autism Spectrum Disorder is a lifelong, neurodevelopmental condition characterized by deficits in social communication and interaction, and restricted, repetitive repertoires of behavior, interests and activities (Diagnostic and Statistical Manual of Mental Disorders-fifth edition; DSM-5 2013; Howlin et al. 2004,2013; Woolfenden et al. 2012). Thus, autistic persons may face challenges and have to rely on support from their families and communities throughout the lifespan. With the rising number of persons diagnosed with autism—up to 3% of children and adolescents in the US and Europe (Baio et al. 2018; Christensen et al. 2018; Delobel-Ayoub et al. 2020; Schendel and Thorsteinsson 2018; Xu et al. 2018)—it is expected that there will be an increasing demand for adult-specific services. However, knowledge concerning evidenced-based services for autistic adults is sparse (Shattuck et al. 2020).

## Diagnosis in Adulthood

Because autistic characteristics are usually evident in early childhood, much of the research on good practices for the identification and diagnosis of autism has focused on children and young people (Happé and Charlton 2012; Mukaetova-Ladinska et al. 2012). Yet many persons with autism begin the autism diagnostic process in adulthood (García-Primo et al. 2014; Happé et al. 2016; Magán-Maganto et al. 2017, Mukaetova-Ladinska et al. 2012; Povey et al. 2011). Autism diagnosis in adulthood faces several challenges: (a) the paucity of adult-specific screening and diagnostic tools (Baghdadli et al. 2017; National Collaborating Centre for Mental Health; Interagency Autism Coordinating Committee 2010, 2011); (b) the possible reduction of symptom severity and co-occurring conditions later in life (Baghdadli et al. 2017; Russell et al. 2016; Tantam 2000; Trammell et al. 2013; Takara and Kondo 2014); (c) poor recall of the individual’s early life developmental history (van Niekerk et al. 2010); (d) cultural factors that may mask autistic signs (DSM-5; APA 2013; Lai and Baron-Cohen 2015; Lai and Lombardo 2011); and (e) limited experience and training in adult autism of many professionals (Nicolaidis et al. 2014). The autistic diagnostic process in adulthood may be also complicated by the overlap between autistic manifestations and other psychiatric or neurodevelopmental conditions (Baghdadli et al. 2017; Cath et al. 2008). Finally, the timing and likelihood of autism identification in females may be further thwarted by diagnostic camouflaging, misdiagnosis or co-morbidities influence (Lai et al. 2015).

## Diagnostic Process and Post-diagnostic Support Experiences

In general, there is very limited research on best practices for diagnosis and post-diagnosis needs of autistic adults and their families to promote good outcomes (Howlin 2013; Magiati et al. 2014). A recent study in the United Kingdom by Crane et al. (2018) used a qualitative methodology to investigate the perceptions and experiences of ten autistic adults, ten parents of autistic children, and eight non-medical and three medical professionals. The responders reported the need for improvements in the ‘process of understanding and accepting autism’, ‘barriers to satisfaction with the diagnostic process’, and ‘inadequate post-diagnosis support provision’ (Crane et al. 2018; p. 3765). A lack of awareness of autism spectrum among professionals, especially family doctors and teachers, also hindered the diagnostic evaluation. Further, the post-diagnostic support was described as directionless, at least until a crisis was reached. Similar results were found in a study by Mukaetova-Ladinska and Stuart-Hamilton (2016) where 31% of autistic adults felt that post-diagnostic support they received was inadequate and the major problem concerned long-term support. Also, from qualitative analysis of interviews by parents of young autistic adults there emerged difficulties in accessing services and inappropriateness of the programs (e.g., bad match for the needs of the autistic person and their family, staff qualifications issues) (Anderson et al. 2018).

## Guidelines on the Diagnostic Process and Post-diagnostic Support for Autistic Adults

It is necessary to develop and implement autistic adult-specific service guidelines, policies and services based on evidence in order to improve users’ and providers’ satisfaction. In England and Wales, efforts in this direction have been provided by the National Institute for Health and Care Excellence (NICE). NICE recommendations for diagnosis and care management of autistic adults were first published in 2012 with minor revisions in subsequent years and included good practices for screening, assessment and interventions with the aim to reduce core symptoms and co-occurring conditions in autistic persons older than 18 years. For instance, the guidelines suggest that an autism evaluation should examine several features such as language and communication, physical or mental conditions, sensory problems, neurodevelopmental conditions (e.g., ADHD), disruptive and self-injurious behaviors and abuse by others. It is advised that biologic or genetic tests or neuroimaging not be used routinely in assessments but may be indicated in individual circumstances (e.g., evidence of dysmorphic features, learning disability, epilepsy). Additionally, it is recommended that the assessment should be done by a multidisciplinary team and that post-diagnostic support for adults should be implemented, for example, via written recommendations for medical issues and health care and how to manage risks and crisis (NICE 2012, NICE 2016). Other European examples of publicly available quality standards for autistic adults’ services are provided by Autism Europe (2013) and the National Audit Office in the United Kingdom (2009). Non-European examples of guidelines for the care of autistic adults are provided by the diagnostic process for children, adolescents and adults referred for assessment of autism spectrum disorder in Australia: A national guideline (Whitehouse et al. 2017), Autism Spectrum Disorders: Missouri best practice guidelines for screening, diagnosis, and assessment (2010), New Zealand Autism Spectrum Disorder Guideline (2016). Despite these resources, however, it is unknown how well or how widely the recommendations are implemented in practice in community settings.

## Aims and Objectives

The present study aimed to explore the real-world knowledge and experiences in diagnostic evaluation and the post-diagnostic support services for autistic adults in Europe and if the experiences were consistent with guidelines and recommendations, in order to identify gaps and opportunities for improvement. Information was gathered via an on-line survey exploring the experiences of current services practices for autistic adults. The survey was carried out by researchers working within the Autism Spectrum Disorder in the European Union (ASDEU 2015–2018) network, a collaboration of 11 European countries (http://asdeu.eu/). The data were used to compare perceptions and experiences of (1) autistic adults, (2) carers of autistic adults and (3) professionals in adult services, to determine the degree of alignment between the respondents’ actual experiences of services and published guidelines. With these data we hoped to identify potential target areas for improving the diagnostic processes and post-diagnostic support in addition to informing policy in this area.

## Method

### Survey Development

Survey questions were based upon a variety of published guidelines and recommendations regarding services for autistic adults (Autism Europe 2013; Kendall et al. 2013; National Audit Office 2009; NICE 2012, 2014a, b, 2016; Think Autism: Updating the 2010 Adult Autism Strategy) which describe quality standards for health and social services for autistic adults and associated approaches for the evaluation and profiling of needs and gaps in services for autistic adults. Response options for many questions were developed to reflect how closely respondents believed that the local services that they had experienced ‘fit’ these recommendations. Thus, the survey results provide a measure of how closely services provision at the local ASDEU level aligned with published guidelines.

Three versions of the survey were developed: for autistic adults; family/caregivers of autistic adults; and administrators/professionals/service providers for adults. The draft surveys went through several stages of revision with input from all ASDEU sites. An autistic adult tested the on-line questionnaire for autistic adults and gave feedback. The survey’s questions and response options are presented in the Supplementary Material 1.

### Survey Description

In the survey’s introduction, responders were instructed to select answers that seemed to fit most closely with what they knew or had experienced and to answer to the best of their knowledge and experience. Questions were written using everyday language and avoiding technical terms that might not be understood or applicable across different countries. The present study used data from two sections of the survey: (1) the demographic characteristics of responders, including 12 question for the autistic adults, nine for carers, and seven for professionals; (2) the experiences and perceptions on the autism diagnostic process and post-diagnostic support, including four subsections: (i) age at diagnosis, waiting time for service and knowledge of local diagnostic services (four questions for autistic adults and carers, and three questions for professionals); (ii) information on how to get a diagnostic evaluation for autism in adulthood (i.e., available on internet, on print, easy to find or understand); (iii) recommended and NOT recommended features for an adult diagnostic evaluation for autism spectrum (13 features for autistic adults, 14 for carers, and 21 for professionals), and (iv) recommended features for autistic adult post-diagnostic support (four features for autistic adults and six each for carers and professionals). The autistic diagnostic and post-diagnostic support section was restricted to respondents who had adult diagnosis experience, e.g., autistic adults who were diagnosed with autism at 18 years of age or older.

### Recruitment and Survey Distribution

The lead site for the adult services component of ASDEU (Denmark) provided all ASDEU partners with information and suggestions on how and to whom surveys could be distributed. Subsequently, all partners sent out survey notices and invitations to participate to autism organizations (national, local, voluntary) and service providers organizations (public and private; including residential facilities, job training and education programs). Furthermore, these organizations were encouraged to publish the survey links through their channels (e-newsletters, websites, or social media accounts). The researchers at each site also disseminated their surveys through their professional networks and on social media. This approach to recruitment was the only feasible process, given the limited resources of the ASDEU study.

The survey was launched in mid-February 2017 in three languages (English, Spanish, and Danish). By mid-September 2017, all three versions of the survey had been launched in 11 languages (English, Spanish, Danish, French, Polish, Icelandic, German, Finnish, Italian, and Romanian, as well as Portuguese for professional version); data for this analysis were based on the total responses obtained up to December 2017.

Each ASDEU site obtained local ethical approval as needed before distributing the survey in their country. All procedures in studies involving human participants were in accordance with the ethical standards of the institutional and/or national research committee and with the World Medical Association Declaration of Helsinki and its later amendments or comparable ethical standards. Prior to starting the survey, respondents had to read the information about the survey and give their informed consent electronically. No personal identifying information was collected. For analysis, data were handled in aggregated form; no feedback to participants was provided nor were individual respondent’s results reported. The background information section of the survey obtained a few demographic characteristics in order to classify the respondent for analysis purposes (e.g., gender, age, highest education level, country of residence, population size of the community where living/working).

### Analysis Methods

Overall, data from the 2009 completed or partially completed surveys were distributed as follows: autistic adults (n = 667), carers of autistic adults (n = 591) and professionals (n = 751). For the purpose of the present study, responses specific to the two sections on demographic characteristics and experiences and perceptions on the autism diagnostic process and post-diagnostic support were analyzed. The survey did not ask autistic adult or carer respondents to provide the source of the adult’s autism diagnosis. The autism diagnostic process and post-diagnostic support section was completed by responders who answered ‘Yes’ to the following questions: ‘were you 18 years of age or older when you got the autism spectrum diagnosis?’ (autistic adult, 53%, n = 356); ‘Did the adult get the autism spectrum diagnosis when he or she was 18 years of age or older?’ (carer, 15%, n = 88); ‘Do you have knowledge of and current work experience (in the last 2 years) in diagnostic procedures in adults and post-diagnostic support for autistic adults?’ (professional, 20%, n = 151). Aggregated descriptive statistics were calculated for all questions. We performed stratified analysis to see if variation in responses was associated with gender of the autistic adults or with level of independence/support needs of the autistic adult reported by carers. Due to the small sample size (n = 11), responders who answered ‘Other/no answer’ to the question about their gender were excluded from the gender stratified analysis.

We also performed two sensitivity analyses. First, we repeated analyses of select questions (knowledge of good local models; diagnostic information easy to find or understand; experience with recommended features for the diagnostic process and post-diagnostic support) by country of residence. Second, we repeated analyses of questions regarding the adult’s or carer’s experience with recommended features for the diagnostic process and post-diagnostic support after excluding respondents who we were able to ascertain with certainty that they had received the ASD diagnosis before publication of the 2012 NICE guidelines (based on comparing the reported age range at completion of the survey and age range for age at diagnosis).

## Results

### Respondent Demographic Characteristics

The demographic characteristics (Supplementary Material 2) are presented for those autistic adults (n = 356) and carers of autistic adults (n = 88) who reported an autism diagnosis at 18 years of age or older and for those professionals (n = 151) who had knowledge of and work experience—in the last 2 years—in diagnostic procedures in adults and post-diagnostic support for autistic adults. Respondents across all three groups were mainly women (autistic adults: 73%; carers: 90%; professionals: 74%), whereas 39% of cared-for autistic adults (by the carers) were women. The age ranges of the majority of respondents were: autistic adult responders, 26–45 years (62%), carers, 46–64 years (59%), and adults cared for by carers, 18–35 years (65%). Almost all autistic adults (97%) reported that they completed the survey by themselves. The largest group of participants lived in Denmark (34%, n = 218, across all 3 respondent groups). The other most-represented countries were Finland (16%, n = 96), France (16%, n = 95), Spain (7%, n = 42), Italy (6%, n = 36), Poland (5%, n = 34), and Iceland (4%, n = 26). The majority of responders (68%, n = 419) lived in cities that are not capital cities and responders were well distributed across communities of different population sizes.

### Autistic Adult Responders’ Education and Employment

The majority of autistic adult responders were not enrolled in educational programs at the time of survey completion (81%, n = 289), but of these 47% (n = 137) had reached college or a university education level. Among the autistic adults who were attending an educational program (part or full time, 19%, n = 67), the majority were enrolled in a college or university education program (79%, n = 53). 50% of the autistic adults reported that they were unemployed (n = 182). The main reason selected for unemployment was having a disability that prevented them from having a job (36%, n = 65) (Supplementary Material 2).

### Carers’ Characteristics and Characteristics of the Autistic Adult They Cared for

More than 70% of carers reported to have known their cared-for autistic adult the adult’s whole life and most carers were the adult’s parents. More than half of carers reported that they were currently employed (53%, n = 47). More than a half of cared-for autistic adults were reported by the carer to have high or some independence, whereas the remainder (31%, n = 27) required a high level of support in daily living or institution-like care (Supplementary Material 2).

### Professional Respondents’ Backgrounds and Characteristics of Their Workplace

Half of the professionals had worked in the adult services and care sector for more than 10 years. Most of the professionals were psychologists (44%, n = 67), psychiatrists (12%, n = 18), or teachers/pedagogues (11%, n = 16). More than a half of professionals’ source of experience and knowledge about services for adults was reported to come from their current job at a city or regional level, while only 16% stated their services knowledge was most closely connected to the national level (Supplementary Material 2).

### Experiences and Perceptions on the Autism Diagnostic Process and Post-diagnostic Support

#### Age at Diagnosis, Waiting Time for Service and Knowledge of Adult Diagnostic Services

Adults and carers were asked to report the age when the adult got the autism spectrum diagnosis. For autistic adults—those who reported to be diagnosed in adulthood—the mean age at diagnosis was 35 years (SD: 4.5, Range: 18–62, n = 349) while cared-for autistic adults’ mean age at diagnosis was 28 years (SD: 4.8, range: 18–68, n = 82). More autistic females (73%, n = 259) were diagnosed in adulthood than males (24%, n = 86). More carers of highly/partially independent autistic adults (69%, n = 61) reported that their adults were diagnosed after 18 years old, than those of autistic adults that needed high-level of support or institution-like care (31%, n = 27) (Supplementary Material 3).

Around 30% of respondents across all three groups reported that the waiting time between the request for a diagnostic evaluation and beginning of the evaluation was between 1 and 3 months. About the same percentage of responders said that the waiting time reached more than 6 months (Table 1). Similar results were observed among autistic females and males, and adults with low and high level of independence (Supplementary Material 3).

**Table 1:** Waiting time for service and knowledge of local diagnostic services

Question	Answer	Autistic adult		Carer		Professional	
		*n (%)*	*N*	*n (%)*	*N*	*n (%)*	*N*
Waiting time between the request for a diagnostic evaluation and the beginning of the evaluation	< 1 month	51 (14.4)	354	5 (7.4)	68	14 (9.3)	150
	1–3 months	107 (30.2)		18 (26.5)		34 (22.7)	
	3–6 months	67 (18.9)		13 (19.1)		36 (24.0)	
	> 6 months	86 (24.3)		25 (36.8)		45 (30.0)	
	Don’t know	43 (12.2)		7 (10.3)		21 (14.0)	
Number of diagnosis services known in the country specific for autistic adults	0	23 (4.3)	537	21 (4.2)	494	1 (0.7)	145
	1–5	104 (19.3)		95 (19.2)		33 (23.9)	
	6–10	13 (2.4)		21 (4.2)		20 (14.5)	
	> 10	35 (6.5)		73 (14.8)		28 (20.3)	
	Don’t know how many in my country	264 (49.2)		205 (41.5)		56 (40.6)	
	Don’t know if there are any specific for autism spectrum in the country	98 (18.3)		79 (16.0)		7 (5.1)	
Knowledge of a good local model of autism diagnosis service	Yes	153 (28.6)	535	151 (30.8)	491	84 (60.9)	138
	No	235 (43.9)		197 (40.1)		22 (15.9)	
	Don’t know	147 (27.5)		143 (29.1)		32 (23.2)	

N of respondents in each group varies since this section was not restricted to persons diagnosed in adulthood or professionals who had knowledge of and work experience in diagnostic procedures in adults and post-diagnosis support for autistic adults

All survey respondents, regardless of age of autism diagnosis, were asked how many diagnosis services in their country were specific for autism spectrum (i.e., autistic adults, n = 439; carers, n = 415; professionals, n = 138; Table 1). About 20% of each respondent group reported that they knew of 1–5 diagnostic services specific for autistic adults in their country, but only 7% of autistic adults and 15% of carers knew of more than 10 services. In comparison, 20% of professionals knew of more than 10 adult-specific services (Table 1). More carers of autistic adults that need high-level of support or institution-like care (21%) knew of > 10 diagnosis services specific for autistic adults than carers of highly/partially independent autistic adults (9%) (Supplementary Material 3). Notably, more than 40% of respondents across all groups answered that they were aware of the presence of some diagnostic services specific for autistic adults in their country, but they did not know exactly how many. All respondents were also asked if, based on their experience and knowledge, they knew of a diagnostic service for adults in their area or elsewhere in their country that worked very well for autistic adults (i.e., autistic adults, n = 535; carers, n = 491; professionals, n = 138; Table 1). A strikingly large proportion of respondents answered ‘No’ to this question (autistic adult: 44%; carer: 40%, professional: 16%) or ‘Don’t know’ (autistic adult: 28%; carer: 29%, professional: 23%) (Table 1). More autistic males (34%) than females (21%) knew of a good local service for autistic adult diagnosis model that worked well, as did carers of highly/partially independent autistic adults (33%) than carers for autistic adults that need high-level of support/institution-like care (26%) (Supplementary Material 3). The pattern of results in the sensitivity analyses by country of residence (Supplementary material 4) for the question on knowledge of a good local model of autism diagnosis service was not substantially different from the overall results (all countries combined).

#### Information on how to get a Diagnostic Evaluation

**Fig. 1:**
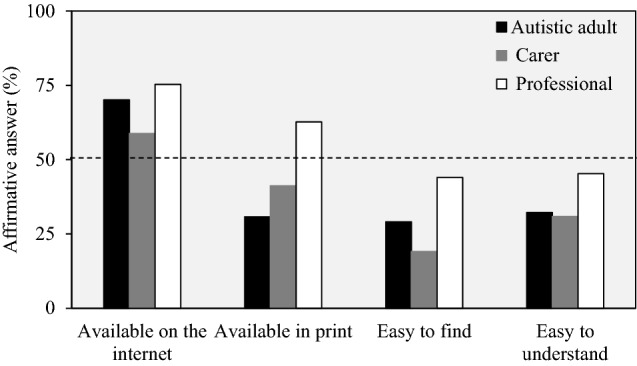
Information on how to get a diagnosis evaluation for autism spectrum in adulthood. Sample size for each answer choice, autistic adult: n = 354; carer: n = 68; professional: n = 150

A higher proportion of professionals compared to autistic adults and carers reported that information on how to get a diagnostic evaluation for autism in adulthood was available on the internet (professionals: 75%; autistic adults: 70%; carers: 59%) or print (professionals: 63%; autistic adults: 31%; carers: 41%; Table 2). Most striking was that 39% of autistic adults, 37% of carers and 20% of professionals reported that they did not know if information on how to get a diagnostic evaluation for autism in adulthood was available in print (Fig. 1; Table 2). More males (39%) reported that information on how to get a diagnosis was available in print than females (29%). More carers of highly/partially independent autistic adults (63%) reported that information was available on the internet than carers of autistic adults that need high-level of support or institution-like care (50%) (Supplementary Material 5).

**Table 2:** Information on how to get a diagnostic evaluation for autism in adulthood

Answer	Autistic adult *(N* = *354)*			Carer *(N* = *68)*			Professional *(N* = *150)*		
	Yes	No	Do not know	Yes	No	Do not know	Yes	No	Do not know
Available on internet	248 (70.1)	50 (14.1)	56 (15.8)	40 (58.8)	10 (14.7)	18 (26.5)	113 (75.3)	19 (12.7)	18 (12.0)
Available in print	109 (30.8)	106 (29.9)	139 (39.3)	28 (41.2)	15 (22.1)	25 (36.8)	94 (62.7)	26 (17.3)	30 (20.0)
Easy to find	103 (29.1)	206 (58.2)	45 (12.7)	13 (19.1)	41 (60.3)	14 (20.6)	66 (44.0)	53 (35.3)	31 (20.7)
Easy to understand	114 (32.2)	178 (50.3)	62 (17.5)	21 (30.9)	30 (44.1)	17 (25.0)	68 (45.3)	50 (33.3)	32 (21.3)

Values expressed as number of responders and frequencies (in parenthesis). The survey question was the following among all groups: *Based on your experience and knowledge, information on how to get a diagnostic evaluation for autism spectrum in adulthood is: …*

Notably 30% or less of the autistic adults or carers and only about 45% of professionals reported that finding or understanding information on how to get a diagnostic evaluation was easy. About 20% of the professionals answered ‘Don’t know’ when asked if the information on how to get a diagnostic evaluation was easy to find or understand (Fig. 1; Table 2). A higher percentage of autistic males answered ‘Yes’ regarding if information on how to get a diagnosis in adulthood was easy to find (37%) or understand (43%) compared to females (27% and 28%, respectively). Also, more carers of highly/partially independent autistic adults reported that information on how to get a diagnosis was easy to find (21%) or understand (33%) than carers of autistic adults that need high-level of support or institution-like care (15% and 25%, respectively) (Supplementary Material 5). The sensitivity analyses by country of residence (Supplementary material 6) did not differ from the overall results (all countries combined).

#### Adult Diagnostic Evaluation

Among all groups, the survey asked about ten recommended features and two features NOT recommended for routine assessments (blood test for genetic studies; brain scan) around the autism diagnostic process. Some questions were specific to autistic adults (i.e., did they complete a questionnaire about symptoms), or carers and professionals (i.e., was there an evaluation of the adult’s development and behavior problems). Finally, questions on seven recommended features and one explicitly not recommended feature for routine evaluations (i.e., biological tests) were asked exclusively of professionals.

Most users and professionals experienced most of the recommended features for the diagnostic evaluation. Among the autistic adults, 65% (n = 225) experienced seven or more of the 11 recommended features. Among carers, 67% (n = 43) experienced nine or more of 12 recommended features. And among professionals, 65% (n = 94) experienced 15 or more of 18 recommended features (Supplementary Material 7).

**Fig. 2:**
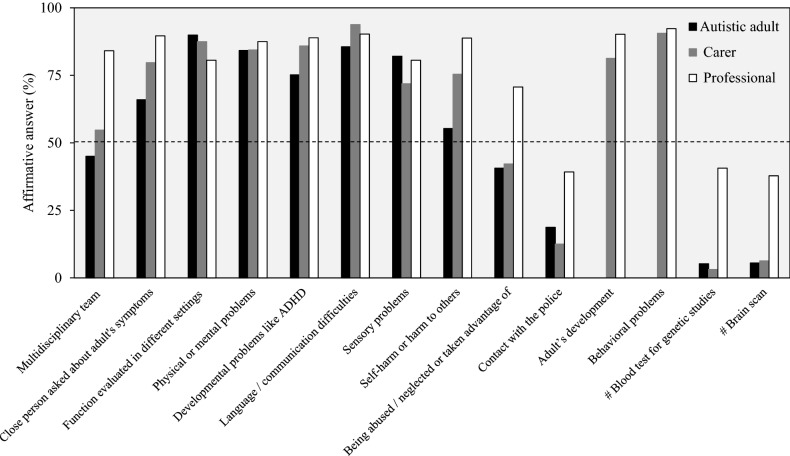
Recommended and NOT recommended features for an adult diagnostic evaluation for autism spectrum. ^#^NOT recommended features (National Institute for Health and Care Excellence 2012). For professionals, the answer choices ‘Standard practice’ or ‘Not standard practice, but often considered’ were considered to be affirmative answers. Sample size for each answer choice, autistic adult: n = 347; carer: n = 64; professional: n = 144 (n = 143, blood test for genetic studies; brain scan; being abused/neglected or taken advantage of; contact with the police)

Specifically, as shown in Fig. 2; Table 3, among each of the three respondent groups (for professionals, considering ‘Standard practice’ and ‘Not standard practice, but often considered’ responses combined) more than 45% experienced each of three recommended features of the autism diagnostic evaluation for adults (i.e., a multidisciplinary team evaluation; a close person was asked about the adult’s symptoms; questions regarding self-harm or harm to others), and over 70% of respondents in each group (considering ‘Standard practice’ and ‘Not standard practice, but often considered’ professional responses combined) experienced each of seven recommended features (i.e., evaluation of functioning in different settings; physical or mental problems; developmental problems; language/communication difficulties; sensory problems, evaluation of behavioral problems; assessment of adult’s development—the last two by carers and professionals). One recommended feature (evaluation of abuse) was experienced by fewer autistic adults or carers (about 40%) than professionals (71% answered that it was a part of the standard routine practice or not standard practice, but often considered). Evaluation of police encounters was experienced by few responders (38% of professionals and less than the 20% of adults or carers).

**Table 3:** Recommended and NOT recommended features for an adult diagnostic evaluation for autism spectrum

Answer	Autistic adult				Carer				Professional					
	Yes	No	Do not know	Total	Yes	No	Do not know	Total	Standard routine practice	Not standard practice, but often considered	Rarely considered	Never considered	Do not know	Total
	*n (%)*			*N*	*n (%)*			*N*	*n (%)*					*N*
Recommended features														
Multidisciplinary team	156 (45.0)	166 (47.8)	25 (7.2)	347	35 (54.7)	28 (43.8)	1 (1.6)	64	96 (66.7)	25 (17.4)	8 (5.6)	5 (3.5)	10 (6.9)	144
Close person asked about adult’s symptoms	229 (66.0)	112 (32.3)	6 (1.7)	347	51 (79.7)	13 (20.3)	0	64	106 (73.6)	23 (16.0)	5 (3.5)	0	10 (6.9)	144
Questionnaire on symptoms	245 (70.6)	92 (26.5)	10 (2.6)	347	N/A				N/A					
Function evaluated in different settings	312 (89.9)	25 (7.2)	10 (2.9)	347	56 (87.5)	7 (10.9)	1 (1.6)	64	94 (65.3)	22 (15.3)	9 (6.2)	6 (4.2)	13 (9.0)	144
Physical or mental problems	292 (84.2)	36 (10.4)	19 (5.5)	347	54 (84.4)	7(10.9)	3 (4.7)	64	111 (77.1)	15 (10.4)	5 (3.5)	1 (0.7)	12 (8.3)	144
Development problems like ADHD	261 (75.2)	58 (16.7)	28 (8.1)	347	55 (85.9)	9 (14.1)	0	64	106 (73.6)	22 (15.3)	1 (0.7)	2 (1.4)	13 (9.0)	144
Language / communication difficulties	297 (85.6)	35 (10.1)	15 (4.3)	347	60 (93.8)	4 (6.3)	0	64	114 (79.2)	16 (11.1)	4 (2.8)	1 (0.7)	9 (6.3)	144
Sensory problems	285 (82.1)	46 (13.3)	16 (4.6)	347	46 (71.9)	12 (18.8)	6 (9.4)	64	90 (62.5)	26 (18.1)	9 (6.2)	5 (3.5)	14 (9.7)	144
Self-harm or harm to others	192 (55.3)	112 (32.3)	43 (12.4)	347	48 (75.4)	12 (18.8)	4 (6.3)	64	100 (69.4)	28 (19.4)	4 (2.8)	0	12 (8.3)	144
Being abused / neglected or taken advantage of	141 (40.6)	161 (46.4)	45 (13.0)	347	27 (42.2)	25 (39.1)	12 (18.8)	64	59 (41.3)	42 (29.4)	10 (7.0)	6 (4.2)	26 (18.2)	143
Contact with the police	65 (18.7)	239 (68.9)	43 (12.4)	347	8 (12.5)	41 (64.1)	15 (23.4)	64	25 (17.5)	31 (21.7)	36 (25.2)	13 (9.1)	38 (26.6)	143
Adult’s development	N/A				52 (81.3)	11 (17.2)	1 (1.6)	64	113 (79.0)	16 (11.2)	1 (0.7)	2 (1.4)	11 (7.7)	143
Behavioral problems	N/A				58 (90.6)	3 (4.7)	3 (4.7)	64	115 (80.4)	17 (11.9)	3 (2.1)	0	8 (5.6)	143
Consultation with other experts	N/A				N/A				74 (51.4)	36 (25.0)	13 (9.0)	4 (2.8)	17 (11.8)	144
Core autistic behaviors	N/A				N/A				116 (81.1)	13 (9.1)	3 (2.1)	0	11 (7.7)	143
Direct observation	N/A				N/A				40 (27.8)	46 (31.9)	23 (16.0)	15 (10.4)	20 (13.9)	144
Standard tests for autism	N/A				N/A				95 (66.0)	24 (16.7)	6 (4.2)	1 (0.7)	18 (12.5)	144
Standard tests for cognitive or psychological functioning	N/A				N/A				100 (69.9)	24 (16.8)	4 (2.8)	0	15 (10.5)	143
Physical examination	N/A				N/A				75 (52.4)	27 (18.9)	11 (7.7)	11 (7.7)	19 (13.3)	143
NOT recommended features														
Blood test for genetic studies	18 (5.2)	320 (92.2)	9 (2.6)	347	2 (3.1)	60 (93.8)	2 (3.1)	64	19 (13.3)	39 (27.3)	33 (23.1)	22 (15.4)	30 (21.0)	143
Brain scan	19 (5.5)	324 (93.4)	4 (1.2)	347	4 (6.3)	58 (90.6)	2 (3.1)	64	24 (16.8)	30 (21.0)	40 (28.0)	16 (11.2)	33 (23.1)	143
Biological tests	N/A				N/A				36 (25.2)	32 (22.4)	27 (18.9)	18 (12.6)	30 (21.0)	143

Recommended and NOT recommended features were retrieved from the National Institute for Health and Care Excellence (2012). N/A = Question was not presented to the respondent group. The questions were the following: autistic adult = *Based on your experience, were the following factors part of your diagnostic evaluation for autism spectrum?*; Carer = *Were the following factors part of the adult’s diagnostic evaluation for autism spectrum?*; Professional = *Thinking of the adult diagnostic service for autism spectrum that you know best, how often are the following factors part of the adult’s diagnostic evaluation for autism spectrum in adults?*

More than 70% of professionals responded that four other recommended features (consultation with other experts; evaluation of autistic core behaviors; use of standard tests for autism and tests for cognitive or psychological functioning; physical examination) were part of the standard routine practice or not standard practice, but often considered during the autistic adults’ diagnosis. Less than 60% of professionals, however, responded that direct observation in social settings were part of the diagnostic assessment (‘Standard practice’ and ‘Not standard practice, but often considered’ responses combined).

More autistic males filled a questionnaire on symptoms during the diagnostic evaluation (74%), and were asked about contact with the police (26%) than females (60%, 17%, respectively) (Supplementary Material 8). More autistic females (range 43–84%) experienced several recommended features (i.e., evaluated for developmental problems like ADHD; sensory problems; being abused/neglected or taken advantage of) compared to males (36–76%) (Supplementary Material 8).

Two features that are explicitly NOT recommended to be part of a standard evaluation (neuroimaging; genetic tests) were claimed to be part of the diagnosis evaluation for less than 6% of autistic adults or carers, while around 40% of professionals reported that these features were standard routine practice or often considered. Biological tests, a NOT recommended feature, was reported to be a standard routine practice or not standard but often considered for 48% of professionals. In addition, more than 20% of professionals said that they did not know if the NOT recommended features were generally part of the standard diagnostic evaluation for autism (Table 3; Fig. 2). Carers of autistic adults with high/partial independence and carers of adults with high need of support reported similar experiences with the NOT recommended features, whereas, among those reporting having experienced the NOT recommended features, more autistic males were present (neuroimaging, 9%; genetic tests, 10%) than females (neuroimaging, 4%; genetic tests, 4%) (Supplementary Material 8). The sensitivity analyses by country of residence (Supplementary material 9) did not differ from the overall results (all countries combined). Only autistic adults living in Denmark, Iceland, United Kingdom, and Germany had experienced less often the presence of a multidisciplinary team in the diagnostic evaluation for autism spectrum (Supplementary material 9). Removing adult and carer respondents who reported the adult’s autism diagnosis occurred at a time before the 2012 publication of NICE guidelines (14 adults in the diagnostic evaluation section and 13 autistic adults in the post-diagnostic support section; 6 carers in both sections) also did not substantially change the pattern of results compared to results based on the full sample (Supplementary material 10).

#### Post-diagnostic Support

Most recommended features for autism post-diagnostic support were not experienced by users. Among autistic adults, 67% (n = 229) experienced none of the recommended features and 70% (n = 45) of carers experienced none or only one recommended feature. In contrast, 61% (n = 85) of professionals experienced—as standard routine practice or often considered—three or more of six recommended features (Supplementary Material 11).

**Fig. 3:**
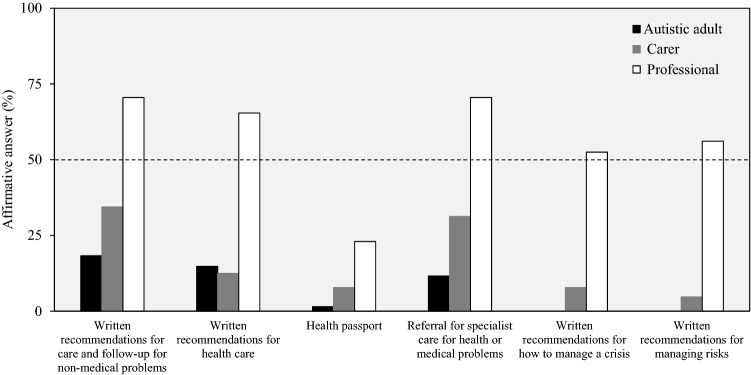
Recommended features for autistic adult post-diagnosis support. For professionals, ‘Standard practice’ and ‘Not standard practice, but often considered’ were considered as affirmative answers. Sample size for each answer choice, autistic adult: n = 344; carer: n = 65 (n = 64, written recommendations for how to manage a crisis); professional: n = 139

Specifically, each of the recommended features (four for autistic adult responders; six for carers and professionals) for autism post-diagnostic support were experienced by less than 35% of autistic adults or carers while between 50 and 60% of autistic adults and carers responded for each recommended feature that it was not experienced, but it was needed. In contrast, between 53 and 71% of professionals reported that each of the recommended post-diagnostic features (except for ‘Health passport’) were ‘standard routine practice’ or ‘not standard but often considered’ (Table 4; Fig. 3). The ‘Health passport’ (to carry important information about the adult’s needs and care) was reported to be a standard practice or often considered by only 23% of professionals and 52% reported that a ‘Health passport’ was never or rarely considered. Across all three groups, between 3 and 27% answered they did not know if each factor of post-diagnostic support was considered in practice (Table 4).

**Table 4:** Recommended features for autistic adult post-diagnosis support

Answer	Autistic adult (N = 344)				Carer (N = 64)				Professional (N = 139)				
	Yes	No, but it was needed	No and it was not needed	Do not know	Yes	No, but it was needed	No and it was not needed	Do not know	Standard routine practice	Not standard practice, but often considered	Rarely considered	Never considered	Do not know
Written recommendations for care and follow-up for non-medical problems	63 (18.3)	182 (52.9)	73 (21.2)	73 (21.2)	22 (34.4)	35 (54.7)	3 (4.7)	4 (6.3)	72 (51.8)	26 (18.7)	12 (8.6)	4 (2.9)	25 (18.0)
Written recommendations for health care	51 (14.8)	175 (50.9)	94 (27.3)	94 (27.3)	8 (12.5)	37 (57.8)	13 (20.3)	6 (9.4)	47 (33.8)	44 (31.6)	17 (12.2)	3 (2.2)	28 (20.1)
‘Health passport’ to carry important information about the adult needs and care	5 (1.5)	205 (59.6)	109 (31.7)	109 (31.7)	5 (7.8)	44 (68.8)	10 (15.6)	5 (7.8)	10 (7.2)	22 (15.8)	33 (23.7)	39 (28.1)	35 (25.2)
Referral for specialist care for health or medical problems	40 (11.6)	171 (49.7)	109 (31.7)	109 (31.7)	20 (31.3)	32 (50.0)	10 (15.6)	2 (3.1)	57 (41.0)	41 (29.5)	16 (11.5)	3 (2.2)	22 (15.8)
Written recommendations for how to manage a crisis	N/A				5 (7.8)	39 (60.9)	13 (20.3)	7 (10.9)	36 (25.9)	37 (26.6)	28 (20.1)	4 (2.9)	34 (24.5)
Written recommendations for managing risks	N/A				3 (4.7)	31 (48.4)	18 (28.1)	12 (18.75)	41 (29.5)	37 (26.6)	21 (15.1)	4 (2.9)	36 (25.9)

Values expressed as number of responders and frequencies (in parenthesis). N/A = Question was not presented to the respondent group. The questions were the following: autistic adult = *After you got the autism spectrum diagnosis, which of the following things happened?*; Carer = *After the adult got the autism spectrum diagnosis, which of the following things happened?*; Professional = *Thinking of the adult diagnostic service for autism spectrum that you know best, how often are the following factors considered as parts of the post-diagnostic activities for autistic adults?*

There were few differences in the post-diagnostic support experience by gender or level of independence of the carer’s adult, except that more autistic males (23%) and carers of higher independence autistic adults (42%) reported receiving written recommendations for care and follow-up for non-medical problems than females (18%) and carers of autistic adults that need a high level of support or institution-like care (16%), respectively. In addition, more carers of higher independence autistic adults (38%) experienced a referral for specialist care for health or medical problems than carers of autistic adults that need high-level support or institution-like care (16%) (Supplementary Material 12).

The pattern of results in the sensitivity analyses by country of residence (Supplementary material 13) was not substantially different from the overall results (all countries combined). Removing adult and carer respondents who reported the adult’s autism diagnosis occurred at a time before the 2012 publication of NICE guidelines also did not substantially change the pattern of results compared to results based on the full sample (Supplementary material 14).

## Discussion

The ASDEU on-line survey on autistic adult services was distributed in 11 European countries with the aim to explore the knowledge of users and providers and their experiences in autistic adult diagnostic services and post-diagnostic support and if their experiences were aligned with published guidelines and recommendations. The survey section on autism diagnostic evaluation and post-diagnostic support services was completed by 595 participants and provides a snapshot of the knowledge and experiences of diagnosis and post-diagnostic services for autistic persons when it occurs in adulthood from three different points of view: autistic adults, carers of autistic adults and professionals in adult services. Knowledge about availability of autism diagnostic services for adults varied considerably by respondent group and adult characteristic but notably less than half of respondents reported that the information on how to get a diagnosis was easy to find or to understand and knowledge of local diagnostic models that work well for autistic adults was generally low. A marked positive finding was that the majority of respondents reported to have experienced the majority of recommended features for an autism diagnostic evaluation in adulthood. In contrast, only a minority of the services users (adults and carers) reported to have experienced the recommended post-diagnostic support services. Overall, the results highlight differences in knowledge and experiences of autistic diagnostic services between users and providers, as well as potential gaps and opportunities for improvement of diagnostic service delivery for autistic adults.

### Respondent Characteristics

The number of respondents that were included in this analysis is less than for the survey overall (53% of autistic adults; 15% of carers and 20% of professionals). This reflects that half of the autistic adults and the majority of the carer’s autistic adults got the autism spectrum diagnosis when they were younger than 18 years of age, and the majority of professionals did not have knowledge of and current work experience (in the last 2 years) in diagnostic procedures in adults and post-diagnostic support for autistic adults.

The present study comprises a convenience sample. However, to date there are no ongoing population-based assessments of demographic characteristics of autistic adults in the EU, therefor it is unknown how the sample might differ from a true, representative sample. Most respondents were females even among autistic adults, which is common for on-line surveys (Smith 2008). This aspect needs to be considered, however, when interpreting the results in view of male–female differences in some autism features which may differentially impact their health and health behaviors (e.g., health care seeking). Thus, autistic male health experiences may be under-represented in the results. Based on achieved education level, employment status and the ability of almost all to complete the questionnaire with no help, the autistic adult respondents likely reflect higher functioning adults than has been observed in previous studies (Happé et al. 2016; Bancroft et al. 2012; Taylor et al. 2015), while the adults cared for by the carers were fairly evenly divided between adults with some level of independence and adults needing a high level of support. Carers were primarily parents of their autistic adult, although there were relatively higher numbers of carers of adults with some level of independence who were not parents than carers of adults needing high level support. On average, the autistic adult responders were somewhat older than the adults cared for by the carers but both groups of adults were generally in their 20 s and 30 s. Half of professional respondents were highly experienced in the adult services and care sector (for more than 10 years) and most commonly were psychologists. Their knowledge and experience about services for adults were mainly relevant to their community or to a larger, regional area. Respondents from all three groups were well distributed across communities of different sizes within their countries and lived in many EU countries. The single largest group of respondents lived in Denmark which may skew some results, but for which questions and in what direction it is difficult to determine with certainty.

### Knowledge of Diagnostic Services and Pre-diagnostic Gathering of Information

Professionals were somewhat more likely to know of more diagnosis services that were specific for autism in adults than autistic adults and carers although more than 40% of respondents across all groups declared that they knew there were adult-specific services for autism diagnosis in their country but they did not know how many. More concerning was that among all survey respondent groups the knowledge of local diagnostic models that work well for autistic adults was generally low (autistic adults and carers: less than 31%; professionals: 61%); autistic females and carers of adults needing a high level of support were least likely to know of good local models.

Among all groups, the majority reported that the information on how to get a diagnostic evaluation for autism in adulthood was available on the internet, although these proportions were lowest for autistic females and carers of adults needing a high level of support. In contrast, less than 50% of autistic adults or carers reported that information on how to get an autism diagnosis in adulthood was available in print, whereas the majority of professionals reported that print material was available. This difference in reported availability of information on the internet or in print by autistic adults and carers should be investigated further to determine if disparities in access to the internet is a barrier to accessing diagnostic services information. Also, of concern was the evidence that less than half of respondents among all the categories reported that information on how to get a diagnosis was easy to find or to understand and these proportions were lowest for autistic females and carers of adults that need high support. Indeed, a more difficult experience by females during the diagnostic process has been observed in Siklos and Kerns (2007). Further, more than 20% of professionals did not know if diagnostic information was easy to find or understand.

Overall, these results suggest that delivery of information on diagnostic services for autistic adults could be improved and especially ensuring that information is easy to find and easy to understand. The results also suggest that gender and level of independence of autistic adults may adversely impact user experiences around the diagnostic process.

Reports of the waiting time between the request for a diagnostic evaluation and the beginning of evaluation was similar among groups: about 30% of the responders reported wait times of between 1 to 3 months and another 30% reported wait times of more than 6 months. It is important to ensure a short waiting time between the request for an evaluation and the evaluation not only for more timely interventions but also to increase service users’ satisfaction (Crane et al. 2016; Jones et al. 2014; Howlin and Moore 1997; Osbourne and Reed 2008; Siklos and Kerns 2007; Smith et al. 1994).

### Alignment with Guidelines: Recommended Characteristics of the Autism Diagnostic Evaluation in Adulthood

Among the three groups, more than the 55% of the responders reported to have experienced the majority of recommended features for the autism diagnostic process for adults including: a close person was asked about the adult’s symptoms, assessment of self-harm or harm to others, presence of language/communication problems, physical or mental conditions, sensory problems, and neurodevelopmental conditions like ADHD, and evaluation of functioning in different settings. For more than a half of professionals, consultation with other experts, evaluation of autistic core behaviors, use of standard tests for autism, tests for cognitive or psychological functioning, and physical examination, were considered as standard routine practice. One of the recommended features—evaluation of police encounters—was, however, experienced by less than 50% of all 3 groups. Further, the recommendation to evaluate a history of abuse was experienced by < 50% of autistic adults and carers while 71% of professionals reported it as a routine practice or often considered. There were notable gender differences in some responses, specifically evaluation of developmental problems, sensory problems, being abused/neglected or being taken advantage of were reported more often by autistic females, while evaluation of contact with the police was reported more often by autistic males. The features that were recommended to NOT be part of the diagnostic process (neuroimaging; genetic tests) were in fact experienced by only a small the minority of adults and carers (3–6%), although around 41–66% of professionals claimed that these NOT recommended features were standard routine or often considered. Again, there was some gender difference with autistic males more often than males reporting that they had experienced the features that were NOT recommended. In summary, results showed that most recommended features of the diagnostic evaluation are often experienced by users and professionals. However, there is room for improvement and in particular regarding evaluation of potential abuses or police encounters and avoiding inclusion of such features as genetic test and brain scan in routine practice. Further, the potential gender differences highlight the need to consider gender in studies of autism diagnosis services.

### Alignment with Guidelines: Recommended Characteristics for Autism Post-diagnostic support for Adults

In contrast to the results for the diagnostic process, all five recommended features for autism post-diagnostic support for adults were experienced by less than 35% of adults and carers and about half of adults and carers reported that they had not experienced the recommended features but that the features were needed. Notably, autistic females and carers of autistic adults that need a higher level of support were least likely to report that they had been provided with written recommendations for care and follow-up for non-medical problems. These results are consistent with reports of lack of post-diagnostic support to be one of the major concerns in other parental and autistic adults’ surveys (Crane et al. 2016; Howlin and Moore 1997; Jones et al. 2014) and the need to improve post-diagnostic support services to enhance quality of life of autistic adults and their families (Mansell and Morris 2004; Siklos and Kerns 2007; Renty and Roeyers 2006).

Notably, over half of professionals reported that each of the recommended features for post-diagnostic support (except provision of a ‘health passport’) was standard routine practice or often considered. These specific results highlight the potential for discrepant views of adult services by professionals and actual experiences by autistic adults and carers. While poor alignment between services recommendations and actual experiences by users has already been observed in previous studies (Crane et al. 2018; Mukaetova-Ladinska and Stuart-Hamilton 2016), this study reveals for the first time the potential for different perceptions of services between autistic adults and carers versus professionals and highlighting the gap in the post-diagnostic support experienced by autistic adults and their carers.

## Limitations

There are several limitations of this study that need to be acknowledged. First of all, the retrieved guidelines and recommendations on which many questions in the survey were based in most cases have not been rigorously, scientifically assessed. Thus, the guidelines may not be optimal for service delivery, but they served as a common starting point against which we could explore service users’, carers’, and professionals’ experiences of local services across ASDEU countries. The NICE guidelines, first published in 2012, were the primary sources of guidance on autism diagnostic services in adulthood and post-diagnostic support when developing the survey. These guidelines were based on expert review of published material on autism diagnosis practices. On the basis of the review and expert evaluation of the evidence, what was deemed best practices was compiled into the guidelines. Our survey was carried out in 2017 and we can be sure that 5% of the autistic adult respondents and 10% cared for adults were diagnosed before 2012; for the remainder we are less certain if they were diagnosed before 2012. In sensitivity analyses, when we removed the minority of respondents who we are certain were diagnosed before 2012, the pattern of results did not change compared to results based on the full sample. We cannot determine if or how results would change if we had more precise information on the timing of diagnosis vis a vis the 2012 guidelines and could limit our analysis to those respondents who were diagnosed after 2012. Nevertheless, what is most striking even in the sensitivity analysis results is the relatively high proportion of respondents who experienced the recommended diagnostic evaluation practices versus the relatively low proportion of respondents—especially adults and carers—who experienced the recommended post-diagnostic support practices. The results suggest a gap in the alignment between best practices and actual experiences in the diagnosis versus post-diagnosis services process. Further study is warranted to determine if, to date, the gap has narrowed, or possibly disappeared.

It is also crucial to note again that the survey results were derived from a convenience sample and it is not known how well this represents the experience of these groups in the European population. Therefore, results may be best considered as a robust ‘pilot study’, providing an overall impression of the state of autistic adult services across different areas of the European Union. Therefore, results and interpretation of data warrant follow-up for confirmation in well-powered and well-designed scientific studies recruiting larger sample sizes. Being a convenience sample, the present study also has the potential for selection bias in the recruitment of respondents, e.g., access to the online survey was restricted to people with internet access, contacts with local associations and respondents who were motivated to respond may not be representative of all services users or professionals. The autistic adults and the adults described by carers may not be directly comparable since the ages, gender distribution and the degree of independence of these two groups appeared to be different. We also do not know the specific circumstances around the diagnosis of the autistic adults (i.e., provider discipline; type of service) which may both validate and impact the diagnosis and post-diagnosis experiences reported by them.

Also, experiences of services reported by users *versus* professionals may not be directly comparable since professionals’ perceptions were based on a large sample of experiences while users’ perceptions were limited to their own personal experience.

Future research should systematically recruit a larger, representative sample of autistic adults/carers and professionals with knowledge of services for autistic adults (Bejarano-Martín et al. 2020).

## Conclusion

The ASDEU survey provided a rich overview of the state of autistic adult diagnostic services and care in 11 countries of the European Union. Knowledge about availability of autism diagnostic services for adults varied considerably by respondent group and adult characteristic but notably less than half of respondents reported that the information on how to get a diagnosis was easy to find or to understand and knowledge of local diagnostic models that work well for autistic adults was generally low. In particular, the results indicated variation in the degree of alignment between published guidelines for services and care for autistic adults and what is actually experienced by services users and services professionals. The alignment between real-world experiences and published guidelines was closest for the diagnostic assessment process while the outlook for post-diagnostic support was much poorer, especially from the perspectives of autistic adults and carers. Discrepancies between actual service experiences and recommendations for services could serve as a guide for what questions to ask and where to focus future effort concerning diagnostic autistic adult services. Differences by gender or degree of independence of autistic adults in diagnostic services and post-diagnostic support experiences highlight the need to consider these important factors in future studies of autistic adult services. Further, discrepancies in experiences between service users and service professionals highlight the need to consider all three respondent groups in order to gain a complete view of services needs of a community (Shattuck et al. 2020). The conclusions and recommendations drawn on the basis of the survey could be used for designing future systematic studies addressing specific topic areas in autism diagnostic assessment and in particular in post-diagnostic support services.

## Supplementary Information

Below is the link to the electronic supplementary material.

Supplementary file1 (DOC 1548 KB)
