# Supplementary material for: Real-World Experiences in Autistic Adult Diagnostic Services and Post-diagnostic Support and Alignment with Services Guidelines: Results from the ASDEU Study
Source: J Autism Dev Disord. 2021 Jan 27;51(11):4129–46. doi: 10.1007/s10803-021-04873-5 (PMC8510906; doi:10.1007/s10803-021-04873-5)
Supplement: Supplementary file 1 — Supplementary file1 (DOC 1548 KB) [file 10803_2021_4873_MOESM1_ESM.doc]

##

**Real-world experiences in autistic adult diagnostic services and post-diagnostic support and alignment with services guidelines: Results from the ASDEU study.**

## Author Note

We have no conflict of interest to disclose.

**Supplementary Material 1**

*Survey’s questions and answer choices for autistic adults, carers, cared-for adults and professionals*

| Question autistic adult | Question carer | Question professional | Answer autistic adult | Answer carer | Answer professional |
| --- | --- | --- | --- | --- | --- |
| Background information | | | | | |
| N/A | N/A | Thinking of your work history, what job or career title fits you best? | N/A | N/A | General practitioner |
|  |  |  |  |  | Psychiatrist |
|  |  |  |  |  | Medical specialist, other than psychiatrist |
|  |  |  |  |  | Nurse |
|  |  |  |  |  | Other medical professional |
|  |  |  |  |  | Psychologist |
|  |  |  |  |  | Social worker |
|  |  |  |  |  | Teacher/pedagogue |
|  |  |  |  |  | Teaching assistant/nursery assistant |
|  |  |  |  |  | Mental health therapist |
|  |  |  |  |  | Physical or occupational therapist |
|  |  |  |  |  | I work in the area of criminal justice (e.g., police, courts, legal advocate) |
|  |  |  |  |  | Other, please specify |
| N/A | N/A | Thinking of your work history, how many years in total have you been in jobs in adult services and care (social, medical, or other services) | N/A | N/A | < 1 year |
|  |  |  |  |  | 1-2 years |
|  |  |  |  |  | 3-5 years |
|  |  |  |  |  | 6-10 years |
|  |  |  |  |  | > 10 years |
| What is your gender? | What is your gender? | What is your gender? | Male | Male | Male |
|  |  |  | Female | Female | Female |
|  |  |  | Other/no answer | Other/no answer | Other/no answer |
| How old are you? | How old are you? | N/A | 18-25 | 18-25 | N/A |
|  |  |  | 26-35 | 26-35 |  |
|  |  |  | 36-45 | 36-45 |  |
|  |  |  | 46-55 | 46-55 |  |
|  |  |  | 56-64 | 56-64 |  |
|  |  |  | > 65 | > 65 |  |
| N/A | How many years of education did you complete? | N/A | N/A | < 10 years | N/A |
|  |  |  |  | 10-12 years |  |
|  |  |  |  | 13-16 years |  |
|  |  |  |  | > 16 years |  |
| Are you going to a school, a home school or in an education program now? | N/A | N/A | Yes, full time | N/A | N/A |
|  |  |  | Yes, part time |  |  |
|  |  |  | No |  |  |
| If no, do you remember your age when you finished your education? | N/A | N/A | Yes | N/A | N/A |
|  |  |  | No |  |  |
| How old were you when you finished your education? | N/A | N/A | _______ | N/A | N/A |
| If yes, what kind of education are you in now? | N/A | N/A | Primary level school | N/A | N/A |
|  |  |  | Secondary level school (for example high school or gymnasium) |  |  |
|  |  |  | Technical, vocational or job training school |  |  |
|  |  |  | College or university |  |  |
| What kind of education were you in when you finished your education: | N/A | N/A | Secondary - level school (for example high school or gymnasium) | N/A | N/A |
|  |  |  | Technical, vocational or job training school |  |  |
|  |  |  | College or university |  |  |
|  |  |  | Don't know |  |  |
|  |  |  | Secondary - level school (for example high school or gymnasium) |  |  |
| Are you: | What is your current employment status now? Pick the answer that fits you best. | N/A | N/A | Student (carer) | N/A |
|  |  |  |  | Unemployed |  |
|  |  |  |  | Employed (part time or full time) |  |
|  |  |  |  | Self-employed (carer) |  |
|  |  |  |  | Retired (carer) |  |
|  |  |  |  | Volunteer |  |
| If you are unemployed, is it because: | N/A | N/A | You are a student | N/A | N/A |
|  |  |  | You are retired |  |  |
|  |  |  | You are looking for a job |  |  |
|  |  |  | You believe that you cannot find a job |  |  |
|  |  |  | You have a disability that prevents you from having a job |  |  |
|  |  |  | Other, please specify |  |  |
| What country do you live in? | What country do you live in? | What country do you work in? | Austria | Austria | Austria |
|  |  |  | Belgium | Belgium | Belgium |
|  |  |  | Bulgaria | Bulgaria | Bulgaria |
|  |  |  | Denmark | Denmark | Denmark |
|  |  |  | England | England | England |
|  |  |  | Finland | Finland | Finland |
|  |  |  | France | France | France |
|  |  |  | Iceland | Iceland | Iceland |
|  |  |  | Italy | Italy | Italy |
|  |  |  | Northern Ireland | Northern Ireland | Northern Ireland |
|  |  |  | Poland | Poland | Poland |
|  |  |  | Portugal | Portugal | Portugal |
|  |  |  | Republic of Ireland | Republic of Ireland | Republic of Ireland |
|  |  |  | Romania | Romania | Romania |
|  |  |  | Scotland | Scotland | Scotland |
|  |  |  | Spain | Spain | Spain |
|  |  |  | Wales | Wales | Wales |
|  |  |  | Other, specify | Other, specify | Other, specify |
| N/A | What is the autistic adult’s gender? | N/A | N/A | Male | N/A |
|  |  |  |  | Female |  |
|  |  |  |  | Other/no answer |  |
| N/A | How old is the autistic adult? | N/A | N/A | 18-25 | N/A |
|  |  |  |  | 26-35 |  |
|  |  |  |  | 36-45 |  |
|  |  |  |  | 46-55 |  |
|  |  |  |  | 56-64 |  |
|  |  |  |  | > 65 |  |
| N/A | How many years have you known the autistic adult? | N/A | N/A | Less than 1 year | N/A |
|  |  |  |  | 1-5 years |  |
|  |  |  |  | 5-10 years |  |
|  |  |  |  | More than 10 years but not the adult's whole life |  |
|  |  |  |  | The adult's whole life |  |
| N/A | How are you related to the autistic adult? | N/A | N/A | Parent | N/A |
|  |  |  |  | Other family member related by blood (not a parent, but for example a child, cousin, grand parent, uncle etc.) |  |
|  |  |  |  | Spouse or partner |  |
|  |  |  |  | A carer, but not a family member, spouse or partner |  |
| Where do you live now? | Where is the autistic adult living now? | Thinking of your current job location where is it located? | Capital city | Capital city | Capital city |
|  |  |  | Other than a capital city | Other than a capital city | Other than a capital city |
| How many people live in the community where you live now? (Answer the best you can) | How many people live in the community where the autistic adult lives now? Answer as best you can | How many people live in the community where your current job is located? | < 1.000 people | < 1.000 people | < 1.000 people |
|  |  |  | 1.000-20.000 people | 1.000-20.000 people | 1.000-20.000 people |
|  |  |  | 20.000-100.000 people | 20.000-100.000 people | 20.000-100.000 people |
|  |  |  | 100.000-1.000.000 | 100.000-1.000.000 | 100.000-1.000.000 |
|  |  |  | > 1.000.000 | > 1.000.000 | > 1.000.000 |
|  |  |  | Don't Know | Don't Know | Don't Know |
| N/A | Pick the description that best fits the autistic adult. | N/A | N/A | Has a high level of independence | N/A |
|  |  |  |  | Has some independence but needs support |  |
|  |  |  |  | Needs a high level of support in daily living |  |
|  |  |  |  | Needs high level institution-like care |  |
| N/A | N/A | The experience and knowledge about services for adults that you have from your current job is: | N/A | N/A | Most closely connected to your current job location (e.g., capital city or small town) |
|  |  |  |  |  | Most closely connected to your current job location and a wider area (e.g., the region or state where your job is located) |
|  |  |  |  |  | Most closely connected to the whole country |
| Autism spectrum diagnosis and support after the diagnosis, in adulthood | | | | | |
| N/A | N/A | Do you have knowledge of and current work experience (in the last 2 years) in diagnostic procedures in adults and post-diagnosis support for autistic adults? | N/A | N/A | Yes |
|  |  |  |  |  | No |
| Were you 18 years of age or older when you got the autism spectrum diagnosis? | Did the adult get the autism spectrum diagnosis when he or she was 18 years of age or older? | N/A | Yes | Yes | N/A |
|  |  |  | I don’t have a formal diagnosis for autism spectrum | No, he/she was less than 18 years old at the time of the autism spectrum diagnosis |  |
|  |  |  | Don't know | He/she does not have a formal autism spectrum diagnosis |  |
|  |  |  |  | Don't know |  |
| If yes, do you remember your age when you got the autism spectrum diagnosis? | If yes, do you remember the adult's age when he/she got the autism spectrum diagnosis? | N/A | Yes, I remember my age | Yes, I remember his/her age | N/A |
|  |  |  | No, I don't remember my exact age at the time, but I was older than 18 | No, I don't remember his/her age at the time, but he/she was older than 18 |  |
| How old were you when you got the autism spectrum diagnosis? | How old was the adult when he/she got the autism spectrum diagnosis? | N/A | _____ | _____ | N/A |
| N/A | You should answer this section ONLY if you have knowledge and experience with the adult’s autism spectrum diagnostic process | N/A | N/A | Yes, I have knowledge and experience with the adult’s diagnostic process | N/A |
|  |  |  |  | No, I do not have knowledge and experience with the adult’s diagnostic process |  |
| Based on your experience and knowledge, information on how to get a diagnostic evaluation for autism spectrum in adulthood in your country, is: | Based on your experience and knowledge, information on how to get a diagnostic evaluation for autism spectrum in adulthood is: | Based on your current work experience and knowledge, information on how to get a diagnostic evaluation for autism spectrum is: | Available on the internet (Yes; No; Don’t know) | Available on the internet (Yes; No; Don’t know) | Available on the internet (Yes; No; Don’t know) |
|  |  |  | Available in print (Yes; No; Don’t know) | Available in print (Yes; No; Don’t know) | Available in print (Yes; No; Don’t know) |
|  |  |  | Easy to find (Yes; No; Don’t know) | Easy to find (Yes; No; Don’t know) | Easy to locate (Yes; No; Don’t know) |
|  |  |  | Easy to understand (Yes; No; Don’t know) | Easy to understand (Yes; No; Don’t know) | Easy to understand (Yes; No; Don’t know) |
| What was the waiting time between the request for a diagnostic evaluation and the beginning of the evaluation for you? | What was the waiting time between the request for a diagnostic evaluation and the beginning of the evaluation for the adult? | Thinking of the adult diagnostic service for autism spectrum that you know best, what is the waiting time between the referral (or the request for an evaluation) and the beginning of the evaluation? | < 1 month | < 1 month | < 1 month |
|  |  |  | 1-3 months | 1-3 months | 1-3 months |
|  |  |  | 3-6 months | 3-6 months | 3-6 months |
|  |  |  | > 6 months | > 6 months | > 6 months |
|  |  |  | Don't know | Don't know | Don't know |
| Based on your experience, were the following factors part of your diagnostic evaluation for autism spectrum? | Were the following factors part of the adult’s diagnostic evaluation for autism spectrum? | Thinking of the adult diagnostic service for autism spectrum that you know best, how often are the following factors parts of the diagnostic evaluation for autism spectrum in adults? | A multidisciplinary team (a team of different kinds of experts) carried out the evaluation (Yes; No; Don’t know) | A multidisciplinary team (a team of different kinds of experts) carried out the evaluation (Yes; No; Don’t know) | Multidisciplinary teams carry out the evaluation (Standard routine practice; Not standard practice, but often considered; Rarely considered; Never considered; Don’t know) |
|  |  |  | N/A | N/A | Consultation takes place with other experts, as needed (Standard routine practice; Not standard practice, but often considered; Rarely considered; Never considered; Don’t know) |
|  |  |  | A family member, carer, partner, or other knowledgeable person was asked about your symptoms (Yes; No; Don’t know) | You or some other knowledgeable person was asked about the adult’s symptoms (Yes; No; Don’t know) | Consultation takes place with a family member, carer, partner, or another knowledgeable person (Standard routine practice; Not standard practice, but often considered; Rarely considered; Never considered; Don’t know) |
|  |  |  | N/A | N/A | Evaluation of core autism spectrum behaviors (Standard routine practice; Not standard practice, but often considered; Rarely considered; Never considered; Don’t know) |
|  |  |  |  |  | Direct observation in social settings (Standard routine practice; Not standard practice, but often considered; Rarely considered; Never considered; Don’t know) |
|  |  |  |  |  | Use of standard tests for autism spectrum (e.g., ADOS) (Standard routine practice; Not standard practice, but often considered; Rarely considered; Never considered; Don’t know) |
|  |  |  |  |  | Use of standard tests for cognitive or psychological functioning (Standard routine practice; Not standard practice, but often considered; Rarely considered; Never considered; Don’t know) |
|  |  |  |  |  | Physical examination (Standard routine practice; Not standard practice, but often considered; Rarely considered; Never considered; Don’t know) |
|  |  |  |  |  | Biological tests (Standard routine practice; Not standard practice, but often considered; Rarely considered; Never considered; Don’t know) |
|  |  |  | You were asked to fill out a questionnaire about your symptoms (Yes; No; Don’t know) | N/A | N/A |
|  |  |  | You had a blood test for genetic studies (Yes; No; Don’t know) | The adult had a genetic test (Yes; No; Don’t know) | Genetic tests (Standard routine practice; Not standard practice, but often considered; Rarely considered; Never considered; Don’t know) |
|  |  |  | You had a scan, for example a brain scan (Yes; No; Don’t know) | The adult had a brain scan (Yes; No; Don’t know) | Neuroimaging (Standard routine practice; Not standard practice, but often considered; Rarely considered; Never considered; Don’t know) |
|  |  |  | N/A | The adult’s development was looked into, for example when the adult first made words (Yes; No; Don’t know) | Evaluation of history of neurodevelopmental disorders (Standard routine practice; Not standard practice, but often considered; Rarely considered; Never considered; Don’t know) |
|  |  |  | N/A | They looked into whether the adult had behavior problems (Yes; No; Don’t know) | Evaluation of behavioral problems (Standard routine practice; Not standard practice, but often considered; Rarely considered; Never considered; Don’t know) |
|  |  |  | You were asked about how well you did in different settings (home, school, job) (Yes; No; Don’t know) | They looked into how well the adult did in different settings (home, school, job) (Yes; No; Don’t know) | Evaluation of functioning in different settings (home, school, job) (Standard routine practice; Not standard practice, but often considered; Rarely considered; Never considered; Don’t know) |
|  |  |  | You were asked if you had physical problems or mental problems (Yes; No; Don’t know) | They looked into whether the adult had physical or mental disorders (Yes; No; Don’t know) | Evaluation of history of physical or other mental comorbid disorders (Standard routine practice; Not standard practice, but often considered; Rarely considered; Never considered; Don’t know) |
|  |  |  | You were asked if you had development problems, like ADHD or learning problems (Yes; No; Don’t know) | They looked into whether the adult had development problems, like ADHD or learning problems (Yes; No; Don’t know) | Evaluation of early developmental history (Standard routine practice; Not standard practice, but often considered; Rarely considered; Never considered; Don’t know) |
|  |  |  | You were asked if you had difficulties with language or the way you communicate with others (Yes; No; Don’t know) | They looked into whether the adult had language or communication problems (Yes; No; Don’t know) | Evaluation of language or communication problems (Standard routine practice; Not standard practice, but often considered; Rarely considered; Never considered; Don’t know) |
|  |  |  | You were asked if you had sensory problems, like being sensitive to noise or bright lights (Yes; No; Don’t know) | They looked into whether the adult had sensory problems, like being sensitive to noise or bright lights (Yes; No; Don’t know) | Evaluation of sensory problems (Standard routine practice; Not standard practice, but often considered; Rarely considered; Never considered; Don’t know) |
|  |  |  | You were asked if you sometimes hurt yourself or hurt others (Yes; No; Don’t know) | They looked into whether the adult hurt him/herself or hurt others (Yes; No; Don’t know) | Evaluation of behavior related to self-harm or harm to others (Standard routine practice; Not standard practice, but often considered; Rarely considered; Never considered; Don’t know) |
|  |  |  | You were asked if you had ever been abused, neglected or been taken advantage of (Yes; No; Don’t know) | They looked into whether the adult had ever been abused, neglected or been taken advantage of (Yes; No; Don’t know) | Evaluation of experience of abuse, neglect or exploitation (Standard routine practice; Not standard practice, but often considered; Rarely considered; Never considered; Don’t know) |
|  |  |  | You were asked if you ever had contact with the police (Yes; No; Don’t know) | They looked into whether the adult ever had contact with the police (Yes; No; Don’t know) | Evaluation of police encounters (Standard routine practice; Not standard practice, but often considered; Rarely considered; Never considered; Don’t know) |
| After you got the autism spectrum diagnosis, which of the following things happened? | After the adult got the autism spectrum diagnosis, which of the following things happened? | Thinking of the adult diagnostic service for autism spectrum that you know best, how often are the following factors considered as parts of the post-diagnostic activities for autistic adults? | Got written recommendations for care and follow-up for non-medical problems (Yes, and it was needed; No, but it was needed; No, and it was NOT needed; Don’t know) | The adult got written recommendations for his/her care and follow-up for non-medical problems (Yes, and it was needed; No, but it was needed; No, and it was NOT needed; Don’t know) | Written recommendations for care and follow-up for non-medical issues, when needed (Standard routine practice; Not standard practice, but often considered; Rarely considered; Never considered; Don’t know) |
|  |  |  | N/A | The adult got written recommendations for managing risks (Yes, and it was needed; No, but it was needed; No, and it was NOT needed; Don’t know) | Written recommendations for managing risks, when needed (Standard routine practice; Not standard practice, but often considered; Rarely considered; Never considered; Don’t know) |
|  |  |  | Got written recommendations for health care (Yes, and it was needed; No, but it was needed; No, and it was NOT needed; Don’t know) | The adult got written recommendations for health care (Yes, and it was needed; No, but it was needed; No, and it was NOT needed; Don’t know) | Written recommendations for health care guidance, when needed (Standard routine practice; Not standard practice, but often considered; Rarely considered; Never considered; Don’t know) |
|  |  |  | N/A | The adult got written recommendations for how to manage a crisis (Yes, and it was needed; No, but it was needed; No, and it was NOT needed; Don’t know) | Written recommendations for crisis management, when needed (Standard routine practice; Not standard practice, but often considered; Rarely considered; Never considered; Don’t know) |
|  |  |  | Got a 'health passport' to carry that has important information about your needs and care (Yes, and it was needed; No, but it was needed; No, and it was NOT needed; Don’t know) | The adult got a 'health passport' to carry that has important information about his/her needs and care (Yes, and it was needed; No, but it was needed; No, and it was NOT needed; Don’t know) | A 'health passport' for the adults to carry with them that has important information about the person's needs and care (Standard routine practice; Not standard practice, but often considered; Rarely considered; Never considered; Don’t know) |
|  |  |  | Got a referral for specialist care for health or medical problems (Yes, and it was needed; No, but it was needed; No, and it was NOT needed; Don’t know) | The adult got a referral for specialist care for health or medical problems  (Yes, and it was needed; No, but it was needed; No, and it was NOT needed; Don’t know) | Referral for specialist care for health or medical conditions, when needed (Standard routine practice; Not standard practice, but often considered; Rarely considered; Never considered; Don’t know) |
| Based on your experience and knowledge, how many diagnosis services in your country are specific for autism spectrum in adults? | Based on your experience and knowledge, how many diagnosis services in your country are specific for autism spectrum in adults? | Based on your current work experience and knowledge, how many diagnostic services in your country are specific for autism spectrum in adults? | 0 | 0 | 0 |
|  |  |  | 1-5 | 1-5 | 1-5 |
|  |  |  | 6-10 | 6-10 | 6-10 |
|  |  |  | > 10 | > 10 | > 10 |
|  |  |  | There are some, but I don't know how many in my country | There are some, but I don't know how many in my country | There are some, but I don't know how many in my country |
|  |  |  | I don't know if there are any that are specific for autism spectrum in my country | I don't know if there are any that are specific for autism spectrum in my country | I don't know if there are any that are specific for autism spectrum in my country |
| Do you know of a diagnostic service for adults, in your area or elsewhere in your country, which works very well for adults on the autism spectrum? | Do you know of a diagnostic service for adults, in your area or elsewhere in your country, which works very well for autistic adults? | Do you know of a diagnostic service for adults, in the "area where you work now" or elsewhere in your country, which has a reputation of exceptional service for diagnosing autism spectrum? | Yes | Yes | Yes |
|  |  |  | No | No | No |
|  |  |  | Don’t know | Don’t know | Don’t know |
| If yes, what is it called? Fill in blank (if you know more than 1, name your first choice) | If yes, what is it called? Fill in blank (if you know more than 1, name your first choice) | If yes, what is it called? Fill in blank (if you know more than 1, name your first choice) | _____ | _____ | _____ |
| If yes, where is it located? Fill in blank (if you know more than 1, name your first choice) | If yes, where is it located? Fill in blank (if you know more than 1, name your first choice) | If yes, where is it located? Fill in blank (if you know more than 1, name your first choice) | _____ | _____ | _____ |

**Supplementary Material 2**

Demographic characteristics of autistic adults, carers, cared-for adults and professionals

| Characteristic | Answer | Autistic adult  *(N=356)* |  | Carer / Cared-for adult  *(N=88)* |  | Professional  *(N=151)* |
| --- | --- | --- | --- | --- | --- | --- |
| Gender | Female | 259 (72.8) |  | 79 (89.8) / 34 (38.6) |  | 112 (74.2) |
|  | Male | 86 (24.2) |  | 9 (10.2) / 54 (61.4) |  | 36 (23.8) |
|  | Other or no answer | 11 (3.9) |  | 0 / 0 |  | 3 (2.0) |
| Age (years) | 18-25 | 40 (11.2) |  | 0/ 21 (23.8) |  | N/A |
|  | 26-35 | 120 (33.7) |  | 10 (11.4) / 36 (40.9) |  |  |
|  | 36-45 | 100 (28.1) |  | 9 (10.2) / 15 (17.1) |  |  |
|  | 46-55 | 77 (21.6) |  | 24 (27.3) / 15 (17.1) |  |  |
|  | 56-64 | 18 (5.1) |  | 28 (31.8) / 1 (1.1) |  |  |
|  | > 65 | 1 (0.3) |  | 17 (19.3) / 0 |  |  |
| Living area | | | | | | |
| Country | Denmark | 157 (44.1) |  | 37 (42.1) |  | 24 (15.9) |
|  | Finland | 49 (13.8) |  | 5 (5.7) |  | 42 (27.8) |
|  | France | 48 (13.5) |  | 19 (21.6) |  | 28 (18.5) |
|  | Spain | 17 (4.8) |  | 10 (11.4) |  | 15 (9.9) |
|  | Italy | 12 (3.4) |  | 4 (4. 6) |  | 20 (13.2) |
|  | Poland | 23 (6.5) |  | 3 (3.4) |  | 8 (5.3) |
|  | Iceland | 15 (4.2) |  | 5 (5.7) |  | 6 (4.0) |
|  | United Kingdom | 21 (5.9) |  | 0 |  | 3 (1.9) |
|  | Republic of Ireland | 9 (2.5) |  | 5 (5.7) |  | 0 |
|  | Germany | 5 (1.4) |  | 0 |  | 3 (1.9) |
|  | Portugal | 0 |  | 0 |  | 2 (1.3) |
| Living area | Capital city | 94 (26.4) |  | 30 (34.1) |  | 52 (34.4) |
|  | Other than a capital city | 262 (73.6) |  | 58 (65.9) |  | 99 (65.6) |
| Community size | < 1.000 people | 24 (6.7) |  | 7 (8.0) |  | 2 (1.3) |
|  | 1.000-20.000 people | 67 (18.8) |  | 21 (23.9) |  | 16 (10.6) |
|  | 20.000-100.000 people | 93 (26.1) |  | 29 (33.0) |  | 46 (30.5) |
|  | 100.000-1.000.000 | 105 (29.5) |  | 16 (18.2) |  | 60 (39.7) |
|  | > 1.000.000 | 37 (10.4) |  | 9 (10.2) |  | 27 (17.9) |
|  | Don't Know | 30 (8.4) |  | 6 (6.8) |  | 0 |
| Education | | | | | | |
| Adult is attending school, home school or education program at the time of survey completion | Yes, full time | 33 (9.3) |  | N/A |  | N/A |
|  | Yes, part time | 34 (9.5) |  |  |  |  |
|  | No | 289 (81.2) |  |  |  |  |
|  |  |  |  |  |  |  |
| If yes, actual education level | Primary level school | 1 (1.5) |  | N/A |  | N/A |
|  | Secondary level school | 6 (9.0) |  |  |  |  |
|  | Technical, vocational or job training school | 7 (10.4) |  |  |  |  |
|  | College or University | 53 (79.1) |  |  |  |  |
| If no, completed education level | Primary level school | 23 (8.0) |  | N/A |  | N/A |
|  | Secondary level school | 49 (17.0) |  |  |  |  |
|  | Technical, vocational or job training school | 72 (24.9) |  |  |  |  |
|  | College or University | 137 (47.4) |  |  |  |  |
|  | Don't Know | 8 (2.8) |  |  |  |  |
| Adult remember the age when completing education | Yes | 262 (90.7) |  | N/A |  | N/A |
|  | No | 27 (9.3) |  |  |  |  |
|  |  |  |  |  |  |  |
| Adult age at completed education | Mean + Standard Deviation [Range] | 29.3 ± 96.7 [6-52] |  | N/A |  | N/A |
|  |  |  |  |  |  |  |
| Carer years of education | < 10 years | N/A |  | 11 (12.5) |  | N/A |
|  | 10-12 years |  |  | 8 (9.1) |  |  |
|  | 13-16 years |  |  | 23 (26.1) |  |  |
|  | >16 years |  |  | 39 (44.3) |  |  |
|  | Other |  |  | 7 (8.0) |  |  |
| Employment | | | | | | |
| Current employment status | Unemployed | 182 (51.1) |  | 10 (11.4) |  | N/A |
|  | Employed (part time o full time) | 124 (34.8) |  | 47 (53.4) |  |  |
|  | Self-employed | 21 (5.9) |  | 3 (3.4) |  |  |
|  | Student | N/A |  | 2 (2.3) |  |  |
|  | Volunteer | 29 (8.2) |  | 2 (2.3) |  |  |
|  | Retired | N/A |  | 24 (27.3) |  |  |
| If unemployed, reasons for unemployment | A disability that prevents from having a job | 65 (35.7) |  | N/A |  | N/A |
|  | Student | 18 (9.9) |  |  |  |  |
|  | Looking for a job | 24 (13.2) |  |  |  |  |
|  | Believing that she/he cannot find a job | 12 (6.6) |  |  |  |  |
|  | Retired | 19 (10.4) |  |  |  |  |
|  | Other | 44 (24.2) |  |  |  |  |
| Adult’s ability to complete the survey with little or no help  *(N=297)* | | | | | | |
| The survey was filled in by autistic adults with little or no help | Alone | 289 (97.3) |  | N/A |  | N/A |
|  | With a parent | 4 (1.6) |  |  |  |  |
|  | With a spouse or partner | 2 (0.7) |  |  |  |  |
|  | With a professional | 0 |  |  |  |  |
|  | Other | 2 (0.7) |  |  |  |  |
| Carer’ relationship with the adult | | | | | | |
| Years of knowledge of the adult | <1 year | N/A |  | 1 (1.1) |  | N/A |
|  | 1-5 years |  |  | 3 (3.4) |  |  |
|  | 5-10 years |  |  | 4 (4.6) |  |  |
|  | >10 years but not the adult's whole life |  |  | 15 (17.1) |  |  |
|  | The adult's whole life |  |  | 65 (73.9) |  |  |
| Relationship with the adult | Parent | N/A |  | 60 (68.2) |  | N/A |
|  | Other family member related by blood |  |  | 9 (10.2) |  |  |
|  | Spouse or partner |  |  | 16 (18.2) |  |  |
|  | A carer, but not a family member, spouse or partner |  |  | 3 (3.4) |  |  |
| Level of independence of the autistic adult | High level of independence | N/A |  | 13 (14.8) |  | N/A |
|  | Some independence but needs support |  |  | 48 (54.6) |  |  |
|  | Needs a high level of support in daily living |  |  | 24 (27.6) |  |  |
|  | Needs high level institution-like care |  |  | 3 (3.4) |  |  |
| Professionals’ backgrounds and characteristics of their workplace | | | | | | |
| Professional type | Psychologist | N/A |  | N/A |  | 67 (44.4) |
|  | Other |  |  |  |  | 18 (11.9) |
|  | Psychiatrist |  |  |  |  | 18 (11.9) |
|  | Teacher/pedagogue |  |  |  |  | 16 (10.6) |
|  | Nurse |  |  |  |  | 7 (4.6) |
|  | General practitioner |  |  |  |  | 7 (4.6) |
|  | Medical specialist, other than psychiatrist |  |  |  |  | 6 (4.0) |
|  | Social worker |  |  |  |  | 4 (2.7) |
|  | Physical or occupational therapist |  |  |  |  | 4 (2.7) |
|  | Teaching assistant/nursery assistant |  |  |  |  | 3 (1.9) |
|  | Other medical professional (open answer) |  |  |  |  | 1 (0.7) |
|  | Mental health therapist |  |  |  |  | 1 (0.7) |
|  | Criminal justice (e.g. police, courts, legal advocate) |  |  |  |  | 0 |
| Years in jobs in adult services and care | <1 year | N/A |  | N/A |  | 6 (4.0) |
|  | 1-2 years |  |  |  |  | 11 (7.3) |
|  | 3-5 years |  |  |  |  | 17 (11.3) |
|  | 6-10 years |  |  |  |  | 40 (26.5) |
|  | >10 years |  |  |  |  | 77 (51.0) |
| Source of experience and knowledge about services for adults | Current job location (e.g., capital city or small town) | N/A |  | N/A |  | 48 (31.8) |
|  | Current job location and a wider area (e.g., the region or state where your job is located) |  |  |  |  | 79 (52.3) |
|  | Most closely connected to the whole country |  |  |  |  | 24 (15.9) |

*Note*. N/A=Question was not presented to the respondent group. Values expressed as number of responders and frequencies (in parenthesis).

**Supplementary material 3**

*Stratified results of age at diagnosis in adulthood, waiting time for service and knowledge of local diagnostic services by autistic by adults’ gender, high/some independence and high level of support/institution-like care of the autistic adult cared reported by carers*

| Question | Answer | Autistic adult | | |  | Carer | | |
| --- | --- | --- | --- | --- | --- | --- | --- | --- |
|  |  | Female | Male | Total |  | High or some independence | High-level of support or institution-like care | Total |
|  |  | *n (%)* | *n (%)* | *N (%)* |  | *n (%)* | *n (%)* | *N (%)* |
| Diagnosis in adulthood | Yes | 259 (75.1) | 86 (24.1) | 345 (100.0) |  | 61 (69.3) | 27 (30.7) | 88 (100.0) |
| Remember the age when the adult got the autism spectrum diagnosis | Yes | 255 (98.5) | 85 (98.8) | 340 (98.6) |  | 60 (98.4) | 22 (81.5) | 82 (93.2) |
|  | No | 4 (1.5) | 1 (1.2) | 5 (1.4) |  | 1 (1.6) | 5 (18.5) | 6 (6.8) |
| Waiting time between the request for a diagnostic evaluation and the beginning of the evaluation | < 1 month | 34 (13.1) | 11 (13.1) | 45 (13.1) |  | 5 (10.4) | 0 (0.0) | 5 (7.4) |
|  | 1-3 months | 81 (31.3) | 23 (27.4) | 104 (30.3) |  | 16 (33.3) | 2 (10.0) | 18 (26.5) |
|  | 3-6 months | 47 (18.2) | 19 (22.6) | 66 (19.2) |  | 8 (16.7) | 5 (25.0) | 13 (19.1) |
|  | > 6 months | 62 (23.9) | 23 (27.4) | 85 (24.78) |  | 15 (31.3) | 10 50.0) | 25 (36.8) |
|  | Don’t know | 35 (13.5) | 8 (9.5) | 43 (12.5) |  | 4 (8.3) | 3 (15.0) | 7 (10.3) |
| Number of diagnosis services known in the country specific for autistic adults | 0 | 16 (4.5) | 6 (3.8) | 22 (4.3) |  | 10 (4.1) | 11 (4.5) | 21 (4.3) |
|  | 1-5 | 67 (18.7) | 30 (18.9) | 97 (18.7) |  | 55 (22.3) | 40 (16.2) | 95 (19.2) |
|  | 6-10 | 10 (2.8) | 3 (1.9) | 13 (2.5) |  | 12 (4.9) | 9 (3.6) | 21 (4.3) |
|  | > 10 | 20 (5.6) | 14 (8.8) | 34 (6.6) |  | 22 (8.9) | 51 (20.7) | 73 (14.8) |
|  | Don't know how many in my country | 176 (49.0) | 82 (51.6) | 258 (49.8) |  | 99 (40.1) | 106 (42.9) | 205 (41.5) |
|  | Don't know if there are any specific for autism spectrum in the country | 70 (19.5) | 24 (15.1) | 94 (18.2) |  | 49 (19.8) | 30 (12.1) | 79 (16.0) |
| Knowledge of good local models of autism diagnosis service | Yes | 92 (25.8) | 54 (34.0) | 146 (28.3) |  | 80 (32.7) | 63 (25.6) | 143 (29.1) |
|  | No | 169 (47.3) | 59 (37.1) | 142 (27.5) |  | 106 (43.3) | 91 (37.0) | 197 (40.1) |
|  | Don’t know | 96 (26.9) | 46 (28.9) | 142 (27.5) |  | 59 (24.1) | 92 (37.4) | 151 (30.8) |

**Supplementary material 4**

Knowledge of a good local model of autism diagnosis service by countries

|  | Autstic adult  (N=535) | | | |  | Carer  (N=491) | | | |  | Professional  (N=138) | | | |
| --- | --- | --- | --- | --- | --- | --- | --- | --- | --- | --- | --- | --- | --- | --- |
| Answer | Yes | No | Do not know | Total |  | Yes | No | Do not know | Total |  | Yes | No | Do not know | Total |
|  | *n (%)* | | | *N* |  | *n (%)* | | | *N* |  | *n (%)* | | | *N* |
| **Knowledge of a good local model of autism diagnosis service** | | | | | | | | | | | | | | |
| Denmark | 28 (13.0) | 116 (53.7) | 72 (33.3) | 216 |  | 25 (17.5) | 64 (44.8) | 54 (37.8) | 143 |  | 8  (38.1) | 6  (28.6) | 7  (33.3) | 21 |
|  |  |  |  |  |  |  |  |  |  |  |  |  |  |  |
| Finland | 22 (32.3) | 24 (35.9) | 22 (32.2) | 68 |  | 11 (28.2) | 9 (23.1) | 19 (48.7) | 39 |  | 15  (39.5) | 6  (15.8) | 17  (44.7) | 38 |
|  |  |  |  |  |  |  |  |  |  |  |  |  |  |  |
| France | 33 (46.5) | 31 (43.7) | 7 (9.9) | 71 |  | 33 (45.8) | 28 (38.9) | 11 (15.3) | 72 |  | 23  (85.2) | 2  (7.4) | 2  (7.4) | 27 |
|  |  |  |  |  |  |  |  |  |  |  |  |  |  |  |
| Spain | 9 (36.0) | 9 (36.0) | 7 (28.0) | 25 |  | 44 (47.3) | 32 (34.4) | 17 (18.3) | 93 |  | 8  (61.5) | 4  (30.8) | 1  (7.7) | 13 |
|  |  |  |  |  |  |  |  |  |  |  |  |  |  |  |
| Italy | 17 (37.0) | 16 (34.8) | 13 (28.3) | 46 |  | 9 (26.5) | 17 (50.0) | 8 (23.5) | 34 |  | 15  (79.0) | 3  (15.8) | 1  (5.3) | 19 |
|  |  |  |  |  |  |  |  |  |  |  |  |  |  |  |
| Poland | 22 (47.8) | 16 (34.8) | 8 (17.4) | 46 |  | 22 (40.0) | 23 (41.8) | 10 (18.2) | 55 |  | 6  (75.0) | 0 | 2  (25.0) | 8 |
|  |  |  |  |  |  |  |  |  |  |  |  |  |  |  |
| Iceland | 4 (20.0) | 8 (40.0) | 8 (40.0) | 20 |  | 6 (13.3) | 18 (40.0) | 21 (46.7) | 45 |  | 3  (75.0) | 1  (25.0) | 0 | 4 |
|  |  |  |  |  |  |  |  |  |  |  |  |  |  |  |
| United Kingdom | 11 (47.8) | 6 (26.1) | 6 (26.1) | 23 |  | 0 | 0 | 0 | 0 |  | 4  (66.7) | 0 | 2  (33.3) | 6 |
|  |  |  |  |  |  |  |  |  |  |  |  |  |  |  |
| Republic of Ireland | 2 (14.3) | 8 (57.1) | 4 (28.6) | 14 |  | 1 (10.0) | 6 (60.0) | 3 (30.0) | 10 |  | 0 | 0 | 0 | 0 |
|  |  |  |  |  |  |  |  |  |  |  |  |  |  |  |
| German | 5 (83.3) | 1 (16.7) | 0 | 6 |  | 0 | 0 | 0 | 0 |  | 0 | 0 | 0 | 0 |
| Portugal | 0 | 0 | 0 | 0 |  | 0 | 0 | 0 | 0 |  | 2  (100.0) | 0 | 0 | 2 |

**Supplementary Material 5**

*Stratified results of information on how to get a diagnostic evaluation for autism in adulthood by autistic by adults’ gender, high/some independence and high level of support/institution-like care of the autistic adult cared reported by carers*

| Answer | Autistic adult  *(N = 343)* | | | | | | | |  | Carer  *(N = 68)* | | | | | | | | | |
| --- | --- | --- | --- | --- | --- | --- | --- | --- | --- | --- | --- | --- | --- | --- | --- | --- | --- | --- | --- |
|  | Yes | | | No | | | Do not know | |  | Yes | | | | No | | | | Do not know | |
|  | Female | Male | Female | | Male | Female | | Male |  | High or some independence | | High-level of support or institution-like care | | High or some independence | | High-level of support or institution-like care | | High or some independence | High-level of support or institution-like care |
| Available on internet | 180 (69.5) | 59 (70.2) | 37 (14.3) | | 11 (13.1) | 42 (16.2) | | 14 (16.7) |  | 30 (62.5) | 10 (50.0) | | 5  (10.4) | | 5  (25.0) | | 13  (27.1) | | 5  (25.0) |
| Available in print | 74 (28.6) | 33 (39.3) | 83 (32.1) | | 19 (22.6) | 102 (39.4) | | 32 (38.1) |  | 20  (41.7) | 8  (40.0) | | 9  (18.8) | | 6  (30.0) | | 19  (39.6) | | 6  (30.0) |
| Easy to find | 69 (26.6) | 31 (36.9) | 154 (59.5) | | 44 (52.4) | 36 (13.9) | | 9  (10.7) |  | 10  (20.8) | 3  (15.0) | | 29  (60.4) | | 12  (60.0) | | 9  (18.8) | | 5  (25.0) |
| Easy to understand | 73 (28.2) | 36 (42.9) | 135 (52.1) | | 37 (44.1) | 51 (19.7) | | 11 (13.1) |  | 16  (33.3) | 5  (25.0) | | 22  (45.8) | | 8  (40.0) | | 10  (20.8) | | 7  (35.0) |

*Note*. Values expressed as number of responders and frequencies (in parenthesis). The original survey question was the following among all groups: *Based on your experience and knowledge, information on how to get a diagnostic evaluation for autism spectrum in adulthood is: ……*.

**Supplementary material 6**

Is information on how to get a diagnostic evaluation easy to find or understand for autism in adulthood? Analysis by countries

| Answer | Autstic adult  (N=354) | | | |  | Carer  (N=68) | | | |  | Professional  (N=150) | | | |
| --- | --- | --- | --- | --- | --- | --- | --- | --- | --- | --- | --- | --- | --- | --- |
|  | Yes | No | Do not know | Total |  | Yes | No | Do not know | Total |  | Yes | No | Do not know | Total |
|  | *n (%)* | | | *N* |  | *n (%)* | | | *N* |  | *n (%)* | | | *N* |
| **Easy to find** |  |  |  |  |  |  |  |  |  |  |  |  |  |  |
| Denmark | 49 (31.4) | 81 (51.9) | 26 (16.7) | 156 |  | 7 (20.6) | 21 (61.8) | 6 (17.6) | 34 |  | 10  (43.5) | 6  (26.1) | 7  (30.4) | 23 |
|  |  |  |  |  |  |  |  |  |  |  |  |  |  |  |
| Finland | 12 (24.5) | 34 (69.4) | 3 (6.1) | 49 |  | 1 (25.0) | 2 (50.0) | 1 (25.0) | 4 |  | 19  (45.2) | 15  (35.7) | 8  (19.1) | 42 |
|  |  |  |  |  |  |  |  |  |  |  |  |  |  |  |
| France | 13 (27.1) | 31 (64.6) | 4 (8.3) | 48 |  | 2 (15.4) | 9 (69.2) | 2 (15.4) | 13 |  | 10  (35.7) | 10  (35.7) | 8  (28.6) | 28 |
|  |  |  |  |  |  |  |  |  |  |  |  |  |  |  |
| Spain | 6 (35.3) | 9 (52.9) | 2 (11.8) | 17 |  | 1 (25.0) | 1 (25.0) | 2 (50.0) | 4 |  | 6  (40.0) | 7  (46.7) | 2  (13.3) | 15 |
|  |  |  |  |  |  |  |  |  |  |  |  |  |  |  |
| Italy | 4 (33.3) | 7 (58.3) | 1 (8.3) | 12 |  | 1 (25.0) | 1 (25.0) | 2 (50.0) | 4 |  | 10  (50.0) | 8  (40.0) | 2  (10.0) | 20 |
|  |  |  |  |  |  |  |  |  |  |  |  |  |  |  |
| Poland | 7 (31.8) | 13 (59.1) | 2 (9.1) | 22 |  | 0 | 1 (100.0) | 0 | 1 |  | 2  (25.0) | 4  (50.0) | 2  (25.0) | 8 |
|  |  |  |  |  |  |  |  |  |  |  |  |  |  |  |
| Iceland | 1 (6.7) | 11 (73.3) | 3 (20.0) | 15 |  | 0 | 3 (100.0) | 0 | 3 |  | 3  (50.0) | 2  (33.3) | 1  (16.7) | 6 |
|  |  |  |  |  |  |  |  |  |  |  |  |  |  |  |
| United Kingdom | 7 (33.3) | 12 (57.1) | 9 (9.5) | 21 |  | 0 | 0 | 0 | 0 |  | 5  (83.3) | 1  (16.7) | 0 | 6 |
|  |  |  |  |  |  |  |  |  |  |  |  |  |  |  |
| Republic of Ireland | 3 (33.3) | 4 (44.4) | 2 (22.2) | 9 |  | 1 (20.0) | 3 (60.0) | 1 (20.0) | 5 |  | 0 | 0 | 0 | 0 |
|  |  |  |  |  |  |  |  |  |  |  |  |  |  |  |
| German | 1 (20.0) | 4 (80.0) | 0 | 5 |  | 0 | 0 | 0 | 0 |  | 0 | 0 | 0 | 0 |
|  |  |  |  |  |  |  |  |  |  |  |  |  |  |  |
| Portugal | 0 | 0 | 0 | 0 |  | 0 | 0 | 0 | 0 |  | 1  (50.0) | 0 | 1  (50.0) | 2 |
|  |  |  |  |  |  |  |  |  |  |  |  |  |  |  |
| **Easy to understand** |  |  |  |  |  |  |  |  |  |  |  |  |  |  |
| Denmark | 47 (30.1) | 75 (48.1) | 34 (21.8) | 156 |  | 7 (20.6) | 16 (47.1) | 11 (32.3) | 34 |  | 8  (34.8) | 8  (34.8) | 7  (30.4) | 23 |
|  |  |  |  |  |  |  |  |  |  |  |  |  |  |  |
| Finland | 15 (30.6) | 25 (51.0) | 9 (18.4) | 49 |  | 1 (25.0) | 2 (50.0) | 1 (25.0) | 4 |  | 19  (45.2) | 12  (28.6) | 11  (26.2) | 42 |
|  |  |  |  |  |  |  |  |  |  |  |  |  |  |  |
| France | 19 (39.6) | 25 (52.1) | 4 (8.3) | 48 |  | 6 (46.1) | 5 (38.5) | 2 (15.4) | 13 |  | 11  (39.3) | 10  (35.7) | 7  (25.0) | 28 |
|  |  |  |  |  |  |  |  |  |  |  |  |  |  |  |
| Spain | 6 (35.3) | 10 (58.8) | 1 (5.9) | 17 |  | 1 (25.0) | 2 (50.0) | 1 (25.0) | 4 |  | 6  (40.0) | 8  (53.3) | 1  (6.7) | 15 |
|  |  |  |  |  |  |  |  |  |  |  |  |  |  |  |
| Italy | 5 (41.7) | 5 (41.7) | 2 (16.7) | 12 |  | 2 (50.0) | 1 (25.0) | 1 (25.0) | 4 |  | 9  (45.0) | 9  (45.0) | 2  (10.0) | 20 |
|  |  |  |  |  |  |  |  |  |  |  |  |  |  |  |
| Poland | 11 (50.0) | 8 (36.4) | 3 (13.6) | 22 |  | 1 (100.0) | 0 | 0 | 1 |  | 6  (75.0) | 1  (12.5) | 1  (12.5) | 8 |
|  |  |  |  |  |  |  |  |  |  |  |  |  |  |  |
| Iceland | 1 (6.7) | 11 (73.3) | 3 (20.0) | 15 |  | 1 (33.3) | 2 (66.7) | 0 | 3 |  | 4  (66.7) | 1  (16.7) | 1  (16.7) | 6 |
|  |  |  |  |  |  |  |  |  |  |  |  |  |  |  |
| United Kingdom | 6 (28.6) | 12 (57.1) | 3 (14.3) | 21 |  | 0 | 0 | 0 | 0 |  | 4  (66.7) | 1  (16.7) | 1  (16.7) | 6 |
|  |  |  |  |  |  |  |  |  |  |  |  |  |  |  |
| Republic of Ireland | 3 (33.3) | 3 (33.3) | 3 (33.3) | 9 |  | 2 (40.0) | 2 (40.0) | 1 (20.0) | 5 |  | 0 | 0 | 0 | 0 |
|  |  |  |  |  |  |  |  |  |  |  |  |  |  |  |
| German | 1 (20.0) | 4 (80.0) | 0 | 5 |  | 0 | 0 | 0 | 0 |  | 0 | 0 | 0 | 0 |
| Portugal | 0 | 0 | 0 | 0 |  | 0 | 0 | 0 | 0 |  | 1  (50.0) | 0 | 1  (50.0) | 2 |

*Note*. The survey question was the following among all groups: *Based on your experience and knowledge, information on how to get a diagnostic evaluation for autism spectrum in adulthood is: …*

**Supplementary material 7**

*Number of recommended features for an adult diagnostic evaluation for autism spectrum* reported by responders

| Number of recommendations | Autistic adult  (N=347) | Carer  (N=64) | Professional  (N=144) |
| --- | --- | --- | --- |
|  | *n (%)* | *n (%)* | *n (%)* |
| 0 | 3 (0.9) | 1 (1.6) | 5 (3.5) |
| 1 | 6 (1.7) | 0 (0.0) | 0 (0.0) |
| 2 | 7 (2.0) | 2 (3.1) | 2 (1.4) |
| 3 | 12 (3.5) | 1 (0.0) | 1 (0.7) |
| 4 | 20 (5.8) | 1 (1.6) | 0 (0.0) |
| 5 | 36 (10.4) | 2 (3.1) | 1 (0.7) |
| 6 | 38 (10.9) | 2 (3.1) | 1 (0.7) |
| 7 | 54 (15.6) | 5 (7.8) | 0 (0.0) |
| 8 | 63 (18.2) | 7 (10.9) | 1 (0.7) |
| 9 | 54 (15.6) | 12 (18.8) | 2 (1.4) |
| 10 | 31 (8.9) | 21 (32.8) | 3 (2.1) |
| 11 | 23 (6.6) | 6 (9.4) | 8 (5.6) |
| 12 |  | 4 (6.3) | 8 (5.6) |
| 13 |  |  | 9 (6.3) |
| 14 |  |  | 9 (6.3) |
| 15 |  |  | 13 (9.0) |
| 16 |  |  | 25 (17.4) |
| 17 |  |  | 22 (15.3) |
| 18 |  |  | 34 (23.6) |

**Supplementary material 8**

Stratified results of information on recommended and NOT recommended features for an adult diagnostic evaluation for autism spectrum by autistic by adults’ gender, high/some independence and high level of support/institution-like care of the autistic adult cared reported by carers

| Answer | Autistic adult  *(N = 337)* | | | | | | |  | | Carer  *(N = 64)* | | | | | |
| --- | --- | --- | --- | --- | --- | --- | --- | --- | --- | --- | --- | --- | --- | --- | --- |
|  | Recommended features | | | | | | | | | | | | | | |
|  | Female | Male | Female | Male | Female | Male | |  | | High or some independence | High-level of support or institution-like care | High or some independence | High-level of support or institution-like care | High or some independence | High-level of support or institution-like care |
|  | Yes | | No | | Do not know | | |  | | Yes | | No | | Do not know | |
| Multidisciplinary team | 116 (45.1) | 33 (41.3) | 117  (45.5) | 46  (57.5) | 24  (9.3) | | 1  (1.3) | |  | 23  (51.1) | 12  (63.2) | 21  (46.7) | 7  (36.8) | 1  (2.2) | 0  (0.0) |
|  |  |  |  |  |  | |  | |  |  |  |  |  |  |  |
| Close person asked about adult’s symptoms | 169 (65.8) | 54 (67.5) | 82  (31.9) | 26  (32.5) | 6  (2.3) | | 0  (0.0) | |  | 37  (82.2) | 14  (73.7) | 8  (17.8) | 5  (26.3) | 0  (0.0) | 0  (0.0) |
|  |  |  |  |  |  | |  | |  |  |  |  |  |  |  |
| Questionnaire on symptoms | 192  (74.7) | 48  (60.0) | 58  (22.6) | 29  (36.3) | 7  (2.7) | | 3  (3.8) | |  | N/A | | | | | |
|  |  |  |  |  |  | |  | |  |  | | | | | |
| Function evaluated in different settings | 230 (89.5) | 73 (91.3) | 20  (7.8) | 4  (5.0) | 7  (2.7) | | 3  (3.8) | |  | 40  (88.9) | 16  (84.2) | 4  (8.9) | 3  (15.8) | 1  (2.2) | 0  (0.0) |
|  |  |  |  |  |  | |  | |  |  |  |  |  |  |  |
| Physical or mental problems | 217 (84.4) | 67 (83.7) | 26  (10.1) | 8  (10.0) | 14  (5.5) | | 5  (6.3) | |  | 40  (88.9) | 14  (73.7) | 4  (8.9) | 3  (15.8) | 1  (2.2) | 2  (10.5) |
|  |  |  |  |  |  | |  | |  |  |  |  |  |  |  |
| Development problems like ADHD | 200 (77.8) | 55 (68.8) | 40  (15.6) | 15  (18.8) | 17  (6.6) | | 10  (12.5) | |  | 40  (88.9) | 15  (79.0) | 5  (11.1) | 4  (21.1) | 0  (0.0) | 0  (0.0) |
|  |  |  |  |  |  | |  | |  |  |  |  |  |  |  |
| Language / communication difficulties | 223 (86.8) | 67 (83.8) | 24  (9.3) | 8  (10.0) | 10  (3.9) | | 5  (6.3) | |  | 41  (91.1) | 19  (100.0) | 4  (8.9) | 0  (0.0) | 0  (0.0) | 0  (0.0) |
|  |  |  |  |  |  | |  | |  |  |  |  |  |  |  |
| Sensory problems | 216 (84.1) | 61 (76.3) | 31  (12.1) | 13  816.3) | 10  (3.9) | | 6  (7.5) | |  | 35  (77.8) | 11  (57.9) | 4  (8.9) | 8  (42.1) | 6  (13.3) | 0  (0.0) |
|  |  |  |  |  |  | |  | |  |  |  |  |  |  |  |
| Self-harm or harm to others | 147 (57.2) | 42 (52.5) | 81  (31.5) | 24  (30.0) | 29  (11.3) | | 14  (17.5) | |  | 33  (73.3) | 15  (79.0) | 8  (17.8) | 4  (21.1) | 4  (8.9) | 0  (0.0) |
|  |  |  |  |  |  | |  | |  |  |  |  |  |  |  |
| Being abused / neglected or taken advantage of | 110 (42.8) | 29 (36.3) | 115  (44.8) | 38  (47.5) | 32  (12.5) | | 13  (16.3) | |  | 19  (42.2) | 8  (42.1) | 16  (35.6) | 9  (47.4) | 10  (22.2) | 2  (10.5) |
|  |  |  |  |  |  | |  | |  |  |  |  |  |  |  |
| Contact with the police | 43 (16.7) | 21 (26.3) | 178  (69.3) | 53  (66.3) | 36  (14.0) | | 6  (7.5) | |  | 5  (11.1) | 3  (15.8) | 26  (57.8) | 15  (79.0) | 14  (31.1) | 1  (5.3) |
|  |  |  |  |  |  | |  | |  |  |  |  |  |  |  |
| Adult’s development | N/A | | | | | | | |  | 39  (86.7) | 13  (68.4) | 5  (11.1) | 6  (31.6) | 1  (2.2) | 0  (0.0) |
|  |  | | | | | | | |  |  |  |  |  |  |  |
| Behavioral problems | N/A | | | | | | | |  | 41  (91.1) | 17  (89.5) | 2  (4.4) | 1  (5.3) | 2  (4.4) | 1  (5.3) |
| NOT recommended features | | | | | | | | | | | | | | | |
| Blood test for genetic studies | 10  (3.9) | 8  (10.0) | 242  (94.2) | 69  (86.3) | 5  (1.9) | | 3  (3.8) | |  | 0  (0.0) | 2  (10.5) | 43  (95.6) | 17  (89.5) | 2  (4.4) | 0  (0.0) |
|  |  |  |  |  |  | |  | |  |  |  |  |  |  |  |
| Brain scan | 11  (4.3) | 7  (8.8) | 244  (94.9) | 71  (88.8) | 2  (0.8) | | 2  (2.5) | |  | 2  (4.4) | 2  (10.5) | 41  (91.1) | 17  (89.5) | 2  (4.4) | 0  (0.0) |

*Note*. *Has a high level of independence* and *Has some independence* *but needs support* are considered *as* High support; *Needs a high level of support in daily living* and *Needs high level institution-like* are considered as Low support*.* N/A = Question was not presented to the respondent group. Recommended and NOT recommended features were retrieved from the National Institute for Health and Care Excellence (2012). The original questions were the following: autistic adult = *Based on your experience, were the following factors part of your diagnostic evaluation for autism spectrum?*; Carer = *Were the following factors part of the adult's diagnostic evaluation for autism spectrum?*; Professional = *Thinking of the adult diagnostic service for autism spectrum that you know best, how often are the following factors part of the adult's diagnostic evaluation for autism spectrum in adults?* Values expressed as number of responders and frequencies (in parenthesis).

**Supplementary material 9**

*Recommended and NOT recommended features* *for an adult diagnostic evaluation for autism spectrum by countries*

| Answer | Autistic adults (N=354) | | | | | | |  | Carer (N=64) | | | |  | Professional (N=143/144) | | | | | |
| --- | --- | --- | --- | --- | --- | --- | --- | --- | --- | --- | --- | --- | --- | --- | --- | --- | --- | --- | --- |
|  | Yes | No | | Do not know | | Total | |  | Yes | No | Do not know | Total |  | Standard routine practice | Not standard practice, but often considered | Rarely considered | Never considered | Do not know | Total |
| **Recommended features** | | | | | | | | | | | | | | | | | | | |
|  | *n (%)* | | | | | *N* | |  | *n (%)* | | | *N* |  | *n (%)* | | | | | *N* |
| **Multidisciplinary team** | | | | | | | | | | | | |  |  |  |  |  |  |  |
| Denmark | 47(30.3) | 96 (61.9) | | 12 (7.7) | | 155 | |  | 15 (46.9) | 16 (50.0) | 1 (3.1) | 32 |  | 12  (52.2) | 2  (8.7) | 1  (4.4) | 2  (8.7) | 6  (26.1) | 23 |
|  |  |  | |  | |  | |  |  |  |  |  |  |  |  |  |  |  |  |
| Finland | 34 (70.8) | 11 (22.9) | | 3 (6.3) | | 48 | |  | 4 (100.0) | 0 | 0 | 4 |  | 32  (80.0) | 5  (12.5) | 1  (2.5) | 1  (2.5) | 1  (2.5) | 40 |
|  |  |  | |  | |  | |  |  |  |  |  |  |  |  |  |  |  |  |
| France | 30 (65.2) | 16 (34.8) | | 0 | | 46 | |  | 7 (58.3) | 5 (41.7) | 0 | 12 |  | 18  (66.7) | 6  (22.2) | 2  (7.4) | 0 | 1  (3.7) | 27 |
|  |  |  | |  | |  | |  |  |  |  |  |  |  |  |  |  |  |  |
| Spain | 7 (43.8) | 7 (43.8) | | 2 (12.4) | | 16 | |  | 2 (50.0) | 2 (50.0) | 0 | 4 |  | 6  (42.9) | 3  (21.4) | 2  (14.3) | 2  (14.3) | 1  (7.4) | 14 |
|  |  |  | |  | |  | |  |  |  |  |  |  |  |  |  |  |  |  |
| Italy | 7 (58.3) | 4 (33.3) | | 1 (8.3) | | 12 | |  | 4 (100.0) | 0 | 0 | 4 |  | 13  (65.0) | 6  (30.0) | 1  (5.0) | 0 | 0 | 20 |
|  |  |  | |  | |  | |  |  |  |  |  |  |  |  |  |  |  |  |
| Poland | 14 (63.6) | 6 (27.3) | | 2 (9.1) | | 22 | |  | 1 (100.0) | 0 | 0 | 1 |  | 6  (75.0) | 1  (12.5) | 1  (12.5) | 0 | 0 | 8 |
|  |  |  | |  | |  | |  |  |  |  |  |  |  |  |  |  |  |  |
| Iceland | 4 (28.6) | 7 (50.0) | | 3 (21.4) | | 14 | |  | 1 (33.3) | 2 (66.7) | 0 | 3 |  | 2  (50.0) | 1  (25.0) | 0 | 0 | 1  (25.0) | 4 |
|  |  |  | |  | |  | |  |  |  |  |  |  |  |  |  |  |  |  |
| United Kingdom | 8 (40.0) | 10 (50.0) | | 2 (10.0) | | 20 | |  | 0 | 0 | 0 | 0 |  | 6  (100.0) | 0 | 0 | 0 | 0 | 6 |
|  |  |  | |  | |  | |  |  |  |  |  |  |  |  |  |  |  |  |
| Republic of Ireland | 3 (33.3) | 6 (66.7) | | 0 | | 9 | |  | 1 (25.0) | 3 (75.0) | 0 | 4 |  | 0 | 0 | 0 | 0 | 0 | 0 |
|  |  |  | |  | |  | |  |  |  |  |  |  |  |  |  |  |  |  |
| German | 2 (40.0) | 3 (60.0) | | 0 | | 5 | |  | 0 | 0 | 0 | 0 |  | 0 | 0 | 0 | 0 | 0 | 0 |
| Portugal | 0 | 0 | | 0 | | 0 | |  | 0 | 0 | 0 | 0 |  | 1 (50.0) | 1  (50.0) | 0 | 0 | 0 | 2 |
| **Close person asked about adult’s symptoms** | | | | | | | | | | | | | | | | | | | |
| Denmark | 109 (70.3) | 41 (26.5) | | 5 (3.2) | | 155 | |  | 24 (75.0) | 8 (25.0) | 0 | 32 |  | 9 (39.1) | 6 (26.1) | 2 (8.7) | 0 | 6 (26.1) | 23 |
|  |  |  | |  | |  | |  |  |  |  |  |  |  |  |  |  |  |  |
| Finland | 33 (68.8) | 14 (29.2) | | 1 (2.1) | | 48 | |  | 4 (100.0) | 0 | 0 | 4 |  | 31 (77.5) | 8 (20.0) | 0 | 0 | 1 (2.5) | 40 |
|  |  |  | |  | |  | |  |  |  |  |  |  |  |  |  |  |  |  |
| France | 23 (50.0) | 23 (50.0) | | 0 | | 46 | |  | 8 (66.7) | 4 (33.3) | 0 | 12 |  | 21 (77.8) | 3 (11.1) | 1 (3.7) | 0 | 2 (7.4) | 27 |
|  |  |  | |  | |  | |  |  |  |  |  |  |  |  |  |  |  |  |
| Spain | 8 (50.0) | 8 (50.0) | | 0 | | 16 | |  | 4 (100.0) | 0 | 0 | 4 |  | 12 (85.7) | 2 (14.3) | 0 | 0 | 0 | 14 |
|  |  |  | |  | |  | |  |  |  |  |  |  |  |  |  |  |  |  |
| Italy | 8 (66.7) | 4 (33.3) | | 0 | | 12 | |  | 4 (100.0) | 0 | 0 | 4 |  | 18 (90.0) | 1 (5.0) | 0 | 0 | 1 (5.0) | 20 |
|  |  |  | |  | |  | |  |  |  |  |  |  |  |  |  |  |  |  |
| Poland | 16 (72.7) | 6 (27.3) | | 0 | | 22 | |  | 1 (100.0) | 0 | 0 | 1 |  | 4 (50.0) | 3 (37.5) | 1 (12.5) | 0 | 0 | 8 |
|  |  |  | |  | |  | |  |  |  |  |  |  |  |  |  |  |  |  |
| Iceland | 10 (71.4) | 4 (28.6) | | 0 | | 14 | |  | 3 (100.0) | 0 | 0 | 3 |  | 3 (75.0) | 0 | 1 (25.0) | 0 | 0 | 4 |
|  |  |  | |  | |  | |  |  |  |  |  |  |  |  |  |  |  |  |
| United Kingdom | 11 (55.0) | 9 (45.0) | | 0 | | 20 | |  | 0 | 0 | 0 | 0 |  | 6 (100.0) | 0 | 0 | 0 | 0 | 6 |
|  |  |  | |  | |  | |  |  |  |  |  |  |  |  |  |  |  |  |
| Republic of Ireland | 8 (88.9) | 1 (11.1) | | 0 | | 9 | |  | 3 (75.0) | 1 (25.0) | 0 | 4 |  | 0 | 0 | 0 | 0 | 0 | 0 |
|  |  |  | |  | |  | |  |  |  |  |  |  |  |  |  |  |  |  |
| German | 3 (60.0) | 2 (40.0) | | 0 | | 5 | |  | 0 | 0 | 0 | 0 |  | 0 | 0 | 0 | 0 | 0 | 0 |
| Portugal | 0 | 0 | | 0 | | 0 | |  | 0 | 0 | 0 | 0 |  | 2 (100.0) | 0 | 0 | 0 | 0 | 2 |
| **Questionnaire on symptoms** | | | | | | | | | | | | | | | | | | | |
| Denmark | 103 (66.5) | 45 (29.0) | | 7 (4.5) | | 155 | |  | N/A | | | |  | N/A | | | | | |
|  |  |  | |  | |  | |  |  |  |  |  |  |  |  |  |  |  |  |
| Finland | 45 (93.7) | 2 (4.2) | | 1 (2.1) | | 48 | |  | N/A | | | |  | N/A | | | | | |
|  |  |  | |  | |  | |  |  |  |  |  |  |  |  |  |  |  |  |
| France | 28 (60.9) | 18 (39.1) | | 0 | | 46 | |  | N/A | | | |  | N/A | | | | | |
|  |  |  | |  | |  | |  |  |  |  |  |  |  |  |  |  |  |  |
| Spain | 12 (75.0) | 3 (18.8) | | 1 (6.2) | | 16 | |  | N/A | | | |  | N/A | | | | | |
|  |  |  | |  | |  | |  |  |  |  |  |  |  |  |  |  |  |  |
| Italy | 10 (83.3) | 2 (16.7) | | 0 | | 12 | |  | N/A | | | |  | N/A | | | | | |
|  |  |  | |  | |  | |  |  |  |  |  |  |  |  |  |  |  |  |
| Poland | 14 (63.6) | 8 (36.4) | | 0 | | 22 | |  | N/A | | | |  | N/A | | | | | |
|  |  |  | |  | |  | |  |  |  |  |  |  |  |  |  |  |  |  |
| Iceland | 11 (78.6) | 3 (21.4) | | 0 | | 14 | |  | N/A | | | |  | N/A | | | | | |
|  |  |  | |  | |  | |  |  |  |  |  |  |  |  |  |  |  |  |
| United Kingdom | 14 (70.0) | 6 (30.0) | | 0 | | 20 | |  | N/A | | | |  | N/A | | | | | |
|  |  |  | |  | |  | |  |  |  |  |  |  |  |  |  |  |  |  |
| Republic of Ireland | 4 (44.4) | 4 (44.4) | | 1 (11.1) | | 9 | |  | N/A | | | |  | N/A | | | | | |
|  |  |  | |  | |  | |  |  |  |  |  |  |  |  |  |  |  |  |
| German | 4 (80.0) | 1 (20.0) | | 0 | | 5 | |  | N/A | | | |  | N/A | | | | | |
|  |  |  | |  | |  | |  |  |  |  |  |  |  |  |  |  |  |  |
| **Function evaluated in different settings** | | | | | | | | | | | | | | | | | | | |
| Denmark | 143 (92.3) | 7 (4.5) | | 5 (3.2) | | 155 | |  | 31 (96.9) | 1 (3.1) | 0 | 32 |  | 16 (69.6) | 2 (8.7) | 0 | 0 | 5 (21.7) | 23 |
|  |  |  | |  | |  | |  |  |  |  |  |  |  |  |  |  |  |  |
| Finland | 44 (91.7) | 3 (6.2) | | 1 (2.1) | | 48 | |  | 3 (75.0) | 0 | 1 (25.0) | 4 |  | 13 (32.5) | 10 (25.0) | 6 (15.0) | 5 (12.5) | 6 (3.7) | 40 |
|  |  |  | |  | |  | |  |  |  |  |  |  |  |  |  |  |  |  |
| France | 41 (89.1) | 4 (8.7) | | 1 (2.2) | | 46 | |  | 7 (58.3) | 5 (41.7) | 0 | 12 |  | 21 (77.8) | 4 (14.8) | 0 | 1 (3.7) | 1 (3.7) | 27 |
|  |  |  | |  | |  | |  |  |  |  |  |  |  |  |  |  |  |  |
| Spain | 15 (93.8) | 0 | | 1 (6.2) | | 16 | |  | 4 (100.0) | 0 | 0 | 4 |  | 13 (92.9) | 0 | 1 (7.1) | 0 | 0 | 14 |
|  |  |  | |  | |  | |  |  |  |  |  |  |  |  |  |  |  |  |
| Italy | 12 (100.0) | 0 | | 0 | | 12 | |  | 3 (75.0) | 1 (25.0) | 0 | 4 |  | 16 (80.0) | 2 (10.0) | 1 (5.0) | 0 | 1 (5.0) | 20 |
|  |  |  | |  | |  | |  |  |  |  |  |  |  |  |  |  |  |  |
| Poland | 22 (100.0) | 0 | | 0 | | 22 | |  | 1 (100.0) | 0 | 0 | 1 |  | 5 (62.5) | 3 (37.5) | 0 | 0 | 0 | 8 |
|  |  |  | |  | |  | |  |  |  |  |  |  |  |  |  |  |  |  |
| Iceland | 4 (28.6) | 10 (71.4) | | 0 | | 14 | |  | 3 (100.0) | 0 | 0 | 3 |  | 3 (75.0) | 0 | 1 (25.0) | 0 | 0 | 4 |
|  |  |  | |  | |  | |  |  |  |  |  |  |  |  |  |  |  |  |
| United Kingdom | 19 (95.0) | 1 (5.0) | | 0 | | 20 | |  | 0 | 0 | 0 | 0 |  | 6 (100.0) | 0 | 0 | 0 | 0 | 6 |
|  |  |  | |  | |  | |  |  |  |  |  |  |  |  |  |  |  |  |
| Republic of Ireland | 7 (77.8) | 0 | | 2 (22.2) | | 9 | |  | 4 (100.0) | 0 | 0 | 4 |  | 0 | 0 | 0 | 0 | 0 | 0 |
|  |  |  | |  | |  | |  |  |  |  |  |  |  |  |  |  |  |  |
| German | 5 (100.0) | 0 | | 0 | | 5 | |  | 0 | 0 | 0 | 0 |  | 0 | 0 | 0 | 0 | 0 | 0 |
| Portugal | 0 | 0 | | 0 | | 0 | |  | 0 | 0 | 0 | 0 |  | 1 (50.0) | 1 (50.0) | 0 | 0 | 0 | 2 |
| **Physical or mental problems** | | | | | | | | | | | | | | | | | | | |
| Denmark | 138 (89.0) | 11 (7.1) | | 6 (3.9) | | 155 | |  | 29 (90.6) | 2 (6.3) | 1 (3.1) | 32 |  | 16 (69.6) | 2 (8.7) | 0 | 0 | 5 (21.7) | 23 |
|  |  |  | |  | |  | |  |  |  |  |  |  |  |  |  |  |  |  |
| Finland | 44 (91.7) | 3 (6.2) | | 1 (2.1) | | 48 | |  | 3 (75.0) | 0 | 1 (25.0) | 4 |  | 30 (75.0) | 3 (7.5) | 3 (7.5) | 0 | 4 (10.0) | 40 |
|  |  |  | |  | |  | |  |  |  |  |  |  |  |  |  |  |  |  |
| France | 37 (80.4) | 6 (13.0) | | 3 (6.6) | | 46 | |  | 7 (58.3) | 5 (41.7) | 0 | 12 |  | 22 (81.5) | 2 (7.4) | 0 | 1 (3.7) | 2 (7.4) | 27 |
|  |  |  | |  | |  | |  |  |  |  |  |  |  |  |  |  |  |  |
| Spain | 10 (62.5) | 6 (37.5) | | 0 | | 16 | |  | 4 (100.0) | 0 | 0 | 4 |  | 12 (85.7) | 1 (7.1) | 1 (7.1) | 0 | 0 | 14 |
|  |  |  | |  | |  | |  |  |  |  |  |  |  |  |  |  |  |  |
| Italy | 8 (66.7) | 3 (25.0) | | 1 (8.3) | | 12 | |  | 4 (100.0) | 0 | 0 | 4 |  | 15 (75.0) | 3 (15.0) | 1 (5.0) | 0 | 1 (5.0) | 20 |
|  |  |  | |  | |  | |  |  |  |  |  |  |  |  |  |  |  |  |
| Poland | 21 (95.5) | 1 (4.5) | | 0 | | 22 | |  | 1 (100.0) | 0 | 0 | 1 |  |  |  |  |  |  |  |
|  |  |  | |  | |  | |  |  |  |  |  |  | 5 (62.5) | 3 (37.5) | 0 | 0 | 0 | 8 |
| Iceland | 8 (57.1) | 4 (28.5) | | 2 (14.3) | | 14 | |  | 2 (66.7) | 0 | 1 (33.3) | 3 |  | 3 (75.0) | 1 (25.0) | 0 | 0 | 0 | 4 |
|  |  |  | |  | |  | |  |  |  |  |  |  |  |  |  |  |  |  |
| United Kingdom | 17 (85.0) | 2 (10.0) | | 1 (5.0) | | 20 | |  | 0 | 0 | 0 | 0 |  | 6 (100.0) | 0 | 0 | 0 | 0 | 6 |
|  |  |  | |  | |  | |  |  |  |  |  |  |  |  |  |  |  |  |
| Republic of Ireland | 5 (55.6) | 0 | | 4 (44.4) | | 9 | |  | 4 (100.0) | 0 | 0 | 4 |  | 0 | 0 | 0 | 0 | 0 | 0 |
|  |  |  | |  | |  | |  |  |  |  |  |  |  |  |  |  |  |  |
| German | 4 (80.0) | 0 | | 0 | | 5 | |  | 0 | 0 | 0 | 0 |  | 0 | 0 | 0 | 0 | 0 | 0 |
| Portugal | 0 | 0 | | 0 | | 0 | |  | 0 | 0 | 0 | 0 |  | 2 (100.0) | 0 | 0 | 0 | 0 | 2 |
| **Development problems like ADHD** | | | | | | | | | | | | | | | | | | | |
| Denmark | 120 (77.4) | 26 (16.8) | | 9 (5.8) | | 155 | |  | 28 (87.5) | 4 (12.5) | 0 | 32 |  | 10 (43.5) | 5 (21.7) | 0 | 0 | 8 (34.8) | 23 |
|  |  |  | |  | |  | |  |  |  |  |  |  |  |  |  |  |  |  |
| Finland | 39 (81.3) | 7 (14.6) | | 2 (4.2) | | 48 | |  | 4 (100.0) | 0 | 0 | 4 |  | 31 (77.5) | 6 (15.0) | 0 | 0 | 3 (7.5) | 40 |
|  |  |  | |  | |  | |  |  |  |  |  |  |  |  |  |  |  |  |
| France | 36 (78.3) | 7 (15.2) | | 3 (6.5) | | 46 | |  | 7 (58.3) | 5 (41.7) | 0 | 12 |  | 24 (88.9) | 1 (3.7) | 0 | 2 (7.4) | 0 | 27 |
|  |  |  | |  | |  | |  |  |  |  |  |  |  |  |  |  |  |  |
| Spain | 10 (62.5) | 4 (25.0) | | 2 (12.5) | | 16 | |  | 4 (100.0) | 0 | 0 | 4 |  | 12 (85.7) | 2 (14.3) | 0 | 0 | 0 | 14 |
|  |  |  | |  | |  | |  |  |  |  |  |  |  |  |  |  |  |  |
| Italy | 10 (83.3) | 2 (16.7) | | 0 | | 12 | |  | 4 (100.0) | 0 | 0 | 4 |  | 13 (65.0) | 4 (20.0) | 1 (5.0) | 0 | 2 (10.0) | 20 |
|  |  |  | |  | |  | |  |  |  |  |  |  |  |  |  |  |  |  |
| Poland | 18 (81.8) | 3 (13.6) | | 1 (4.6) | | 22 | |  | 1 (100.0) | 0 | 0 | 1 |  | 5 (62.5) | 3 (37.5) | 0 | 0 | 0 | 8 |
|  |  |  | |  | |  | |  |  |  |  |  |  |  |  |  |  |  |  |
| Iceland | 7 (50.0) | 4 (28.6) | | 3 (21.4) | | 14 | |  | 3 (100.0) | 0 | 0 | 3 |  | 4 (100.0) | 0 | 0 | 0 | 0 | 4 |
|  |  |  | |  | |  | |  |  |  |  |  |  |  |  |  |  |  |  |
| United Kingdom | 14 (70.0) | 3 (15.0) | | 3 (15.0) | | 20 | |  | 0 | 0 | 0 | 0 |  | 6 (100.0) | 0 | 0 | 0 | 0 | 6 |
|  |  |  | |  | |  | |  |  |  |  |  |  |  |  |  |  |  |  |
| Republic of Ireland | 3 (33.3) | 2 (22.2) | | 4 (44.4) | | 9 | |  | 4 (100.0) | 0 | 0 | 4 |  | 0 | 0 | 0 | 0 | 0 | 0 |
|  |  |  | |  | |  | |  |  |  |  |  |  |  |  |  |  |  |  |
| German | 4 (80.0) | 0 | | 1 (20.0) | | 5 | |  | 0 | 0 | 0 | 0 |  | 0 | 0 | 0 | 0 | 0 | 0 |
| Portugal | 0 | 0 | | 0 | | 0 | |  | 0 | 0 | 0 | 0 |  | 1 (50.0) | 1 (50.0) | 0 | 0 | 0 | 2 |
| **Language/communication difficulties** | | | | | | | | | | | | | | | | | | | |
| Denmark | 135 (87.1) | 14 (9.0) | | 6 (3.9) | | 155 | |  | 30 (93.8) | 2 (6.2) | 0 | 32 |  | 18 (78.3) | 1 (4.4) | 0 | 0 | 4 (17.4) | 23 |
|  |  |  | |  | |  | |  |  |  |  |  |  |  |  |  |  |  |  |
| Finland | 41 (85.4) | 6 (12.5) | | 1 (2.1) | | 48 | |  | 4 (100.0) | 0 | 0 | 4 |  | 31 (77.5) | 5 (12.5) | 2 (5.0) | 0 | 2 (5.0) | 40 |
|  |  |  | |  | |  | |  |  |  |  |  |  |  |  |  |  |  |  |
| France | 40 (87.0) | 5 (10.9) | | 1 (2.2) | | 46 | |  | 10 (83.3) | 2 (16.7) | 0 | 12 |  | 21 (77.8) | 3 (11.1) | 0 | 1 (3.7) | 2 (7.4) | 27 |
|  |  |  | |  | |  | |  |  |  |  |  |  |  |  |  |  |  |  |
| Spain | 13 (81.3) | 3 (18.7) | | 0 | | 16 | |  | 4 (100.0) | 0 | 0 | 4 |  | 13 (92.9) | 1 (7.1) | 0 | 0 | 0 | 14 |
|  |  |  | |  | |  | |  |  |  |  |  |  |  |  |  |  |  |  |
| Italy | 11 (91.7) | 1 (8.3) | | 0 | | 12 | |  | 4 (100.0) | 0 | 0 | 4 |  | 15 (75.0) | 2 (10.0) | 2 (10.0) | 0 | 1 (5.0) | 20 |
|  |  |  | |  | |  | |  |  |  |  |  |  |  |  |  |  |  |  |
| Poland | 21 (95.5) | 1 (4.5) | | 0 | | 22 | |  | 1 (100.0) | 0 | 0 | 1 |  | 6 (75.0) | 2 (25.0) | 0 | 0 | 0 | 8 |
|  |  |  | |  | |  | |  |  |  |  |  |  |  |  |  |  |  |  |
| Iceland | 10 (71.4) | 1 (7.1) | | 3 (21.4) | | 14 | |  | 3 (100.0) | 0 | 0 | 3 |  | 3 (75.0) | 1 (25.0) | 0 | 0 | 0 | 4 |
|  |  |  | |  | |  | |  |  |  |  |  |  |  |  |  |  |  |  |
| United Kingdom | 18 (90.0) | 1 (5.0) | | 1 (5.0) | | 20 | |  | 0 | 0 | 0 | 0 |  | 6 (100.0) | 0 | 0 | 0 | 0 | 0 |
|  |  |  | |  | |  | |  |  |  |  |  |  |  |  |  |  |  |  |
| Republic of Ireland | 4 (44.4) | 2 (22.2) | | 3 (33.4) | | 9 | |  | 4 (100.0) | 0 | 0 | 4 |  | 0 | 0 | 0 | 0 | 0 | 0 |
|  |  |  | |  | |  | |  |  |  |  |  |  |  |  |  |  |  |  |
| German | 4 (80.0) | 1 (20.0) | | 0 | | 5 | |  | 0 | 0 | 0 | 0 |  | 0 | 0 | 0 | 0 | 0 | 0 |
| Portugal | 0 | 0 | | 0 | | 0 | |  | 0 | 0 | 0 | 0 |  | 1 (50.0) | 1 (50.0) | 0 | 0 | 0 | 0 |
| **Sensory problems** | | | | | | | | | | | | | | | | | | | |
| Denmark | 123 (79.4) | 23 (14.8) | | 9 (5.8) | | 155 | |  | 22 (68.8) | 7 (21.8) | 3 (9.4) | 32 |  | 9 (39.1) | 5 (21.7) | 1 (4.4) | 1 (4.4) | 7 (30.4) | 23 |
|  |  |  | |  | |  | |  |  |  |  |  |  |  |  |  |  |  |  |
| Finland | 43 (89.6) | 4 (8.3) | | 1 (2.1) | | 48 | |  | 2 (50.0) | 0 | 2 (50.0) | 4 |  | 27 (67.5) | 5 (12.5) | 1 (2.5) | 3 (7.5) | 4 (10.0) | 40 |
|  |  |  | |  | |  | |  |  |  |  |  |  |  |  |  |  |  |  |
| France | 41 (89.1) | 4 (8.7) | | 1 (2.2) | | 46 | |  | 8 (66.7) | 4 (33.3) | 0 | 12 |  | 18 (66.7) | 7 (25.9) | 0 | 1 (3.7) | 1 (3.7) | 27 |
|  |  |  | |  | |  | |  |  |  |  |  |  |  |  |  |  |  |  |
| Spain | 11 (68.8) | 5 (31.2) | | 0 | | 16 | |  | 4 (100.0) | 0 | 0 | 4 |  | 11 (78.6) | 1 (7.1) | 2 (14.2) | 0 | 0 | 14 |
|  |  |  | |  | |  | |  |  |  |  |  |  |  |  |  |  |  |  |
| Italy | 12 (100.0) | 0 | | 0 | | 12 | |  | 4 (100.0) | 0 | 0 | 4 |  | 13 (65.0) | 3 (15.0) | 3 (15.0) | 0 | 1 (5.0) | 20 |
|  |  |  | |  | |  | |  |  |  |  |  |  |  |  |  |  |  |  |
| Poland | 21 (95.5) | 0 | | 1 (4.5) | | 22 | |  | 1 (100.0) | 0 | 0 | 1 |  | 5 (62.5) | 2 (25.0) | 1 (12.5) | 0 | 0 | 8 |
|  |  |  | |  | |  | |  |  |  |  |  |  |  |  |  |  |  |  |
| Iceland | 6 (42.9) | 6 (42.9) | | 2 (14.3) | | 14 | |  | 2 (66.7) | 0 | 1 (33.3) | 3 |  | 1 (25.0) | 2 (50.0) | 0 | 0 | 1 (25.0) | 4 |
|  |  |  | |  | |  | |  |  |  |  |  |  |  |  |  |  |  |  |
| United Kingdom | 18 (90.0) | 2 (10.0) | | 0 | | 20 | |  | 0 | 0 | 0 | 0 |  | 5 (83.3) | 1 (16.7) | 0 | 0 | 0 | 6 |
|  |  |  | |  | |  | |  |  |  |  |  |  |  |  |  |  |  |  |
| Republic of Ireland | 6 (66.7) | 2 (22.2) | | 1 (11.1) | | 9 | |  | 3 (75.0) | 1 (25.0) | 0 | 4 |  | 0 | 0 | 0 | 0 | 0 | 0 |
|  |  |  | |  | |  | |  |  |  |  |  |  |  |  |  |  |  |  |
| German | 4 (80.0) | 0 | | 1 (20.0) | | 5 | |  | 0 | 0 | 0 | 0 |  | 0 | 0 | 0 | 0 | 0 | 0 |
| Portugal | 0 | 0 | | 0 | | 0 | |  | 0 | 0 | 0 | 0 |  | 1 (50.0) | 0 | 1 (50.0) | 0 | 0 | 2 |
| **Self-harm or harm to others** | | | | | | | | | | | | | | | | | | | |
| Denmark | 92 (59.4) | 49 (31.6) | | 14 (9.0) | | 155 | |  | 24 (75.0) | 6 (18.8) | 2 (6.2) | 32 |  | 13 (56.5) | 3 (13.0) | 0 | 0 | 7 (30.4) | 23 |
|  |  |  | |  | |  | |  |  |  |  |  |  |  |  |  |  |  |  |
| Finland | 27 (56.2) | 13 (27.1) | | 8 (16.7) | | 48 | |  | 2 (50.0) | 0 | 2 (50.0) | 4 |  | 29 (72.5) | 6 (15.0) | 2 (5.0) | 0 | 3 (7.5) | 40 |
|  |  |  | |  | |  | |  |  |  |  |  |  |  |  |  |  |  |  |
| France | 24 (52.2) | 18 (39.1) | | 4 (8.7) | | 46 | |  | 9 (75.0) | 3 (25.0) | 0 | 12 |  | 16 (59.3) | 9 (33.3) | 1 (3.7) | 0 | 1 (3.7) | 27 |
|  |  |  | |  | |  | |  |  |  |  |  |  |  |  |  |  |  |  |
| Spain | 9 (56.3) | 3 (18.7) | | 4 (25.0) | | 16 | |  | 3 (75.0) | 1 (25.0) | 0 | 4 |  | 12 (85.7) | 2 (14.3) | 0 | 0 | 0 | 14 |
|  |  |  | |  | |  | |  |  |  |  |  |  |  |  |  |  |  |  |
| Italy | 8 (66.7) | 4 (33.3) | | 0 | | 12 | |  | 4 (100.0) | 0 | 0 | 4 |  | 16 (80.0) | 3 (15.0) | 0 | 0 | 1 (5.0) | 20 |
|  |  |  | |  | |  | |  |  |  |  |  |  |  |  |  |  |  |  |
| Poland | 12 (54.6) | 7 (31.8) | | 3 (13.6) | | 22 | |  | 1 (100.0) | 0 | 0 | 1 |  | 4 (50.0) | 3 (37.5) | 1 (12.5) | 0 | 0 | 8 |
|  |  |  | |  | |  | |  |  |  |  |  |  |  |  |  |  |  |  |
| Iceland | 6 (42.9) | 6 (42.9) | | 2 (14.3) | | 14 | |  | 2 (66.7) | 1 (33.3) | 0 | 3 |  | 2 (50.0) | 2 (50.0) | 0 | 0 | 0 | 4 |
|  |  |  | |  | |  | |  |  |  |  |  |  |  |  |  |  |  |  |
| United Kingdom | 9 (45.0) | 7 (35.0) | | 4 (20.0) | | 20 | |  | 0 | 0 | 0 | 0 |  | 6 (100.0) | 0 | 0 | 0 | 0 | 6 |
|  |  |  | |  | |  | |  |  |  |  |  |  |  |  |  |  |  |  |
| Republic of Ireland | 3 (33.3) | 4 (44.4) | | 2 (22.2) | | 9 | |  | 3 (75.0) | 1 (25.0) | 0 | 4 |  | 0 | 0 | 0 | 0 | 0 | 0 |
|  |  |  | |  | |  | |  |  |  |  |  |  |  |  |  |  |  |  |
| German | 2 (40.0) | 1 (20.0) | | 2 (40.0) | | 5 | |  | 0 | 0 | 0 | 0 |  | 0 | 0 | 0 | 0 | 0 | 0 |
| Portugal | 0 | 0 | | 0 | | 0 | |  | 0 | 0 | 0 | 0 |  | 2 (100.0) | 0 | 0 | 0 | 0 | 2 |
| **Being abused/ neglected or taken advantage of** | | | | | | | | | | | | | | | | | | | |
| Denmark | 63 (40.6) | 71 (45.8) | | 21 (13.6) | | 155 | |  | 12 (37.5) | 13 (40.6) | 7 (21.9) | 32 |  | 9 (39.1) | 4 (17.4) | 0 | 0 | 10 (43.5) | 23 |
|  |  |  | |  | |  | |  |  |  |  |  |  |  |  |  |  |  |  |
| Finland | 17 (35.4) | 24 (50.0) | | 7 (14.6) | | 48 | |  | 2 (50.0) | 0 | 2 (50.0) | 4 |  | 24 (60.0) | 8 (20.09 | 2 (5.0) | 1 (2.5) | 5 (12.5) | 40 |
|  |  |  | |  | |  | |  |  |  |  |  |  |  |  |  |  |  |  |
| France | 24 (52.2) | 19 (41.3) | | 3 (6.5) | | 46 | |  | 5 (41.7) | 6 (50.0) | 1 (8.3) | 12 |  | 5 (18.5) | 10 (37.0) | 4 (14.8) | 2 (7.4) | 6 (22.2) | 27 |
|  |  |  | |  | |  | |  |  |  |  |  |  |  |  |  |  |  |  |
| Spain | 6 (37.5) | 6 (37.5) | | 4 (25.0) | | 16 | |  | 0 | 4 (100.0) | 0 | 4 |  | 5 (38.5) | 6 (46.2) | 2 (15.4) | 0 | 0 | 13 |
|  |  |  | |  | |  | |  |  |  |  |  |  |  |  |  |  |  |  |
| Italy | 5 (41.7) | 7 (58.3) | | 0 | | 12 | |  | 3 (75.0) | 0 | 1 (25.0) | 4 |  | 6 (30.0) | 7 (35.0) | 2 (10.0) | 2 (10.0) | 3 (15.0) | 20 |
|  |  |  | |  | |  | |  |  |  |  |  |  |  |  |  |  |  |  |
| Poland | 9 (40.9) | 12 (54.6) | | 1 (4.5) | | 22 | |  | 1 (100.0) | 0 | 0 | 1 |  | 2 (25.0) | 5 (62.5) | 0 | 1 (12.5) | 0 | 8 |
|  |  |  | |  | |  | |  |  |  |  |  |  |  |  |  |  |  |  |
| Iceland | 4 (28.6) | 6 (42.9) | | 4 (28.6) | | 14 | |  | 2 (66.7) | 0 | 1 (33.3) | 3 |  | 1 (25.0) | 1 (25.0) | 0 | 0 | 2 (50.0) | 4 |
|  |  |  | |  | |  | |  |  |  |  |  |  |  |  |  |  |  |  |
| United Kingdom | 9 (45.0) | 9 (45.0) | | 2 (10.0) | | 20 | |  | 0 | 0 | 0 | 0 |  | 6 (100.0) | 0 | 0 | 0 | 0 | 0 |
|  |  |  | |  | |  | |  |  |  |  |  |  |  |  |  |  |  |  |
| Republic of Ireland | 2 (22.2) | 4 (44.4) | | 3 (33.3) | | 9 | |  | 2 (50.0) | 2 (50.0) | 0 | 4 |  | 0 | 0 | 0 | 0 | 0 | 0 |
|  |  |  | |  | |  | |  |  |  |  |  |  |  |  |  |  |  |  |
| German | 2 (40.0) | 3 (60.0) | | 0 | | 5 | |  | 0 | 0 | 0 | 0 |  | 0 | 0 | 0 | 0 | 0 | 0 |
| Portugal | 0 | 0 | | 0 | | 0 | |  | 0 | 0 | 0 | 0 |  | 1 (50.0) | 1 (50.0) | 0 | 0 | 0 | 0 |
| **Contact with the police** | | | | | | | | | | | | | | | | | | | |
| Denmark | 29 (18.7) | 103 (66.5) | | 23 (14.8) | | 155 | |  | 3 (9.4) | 18 (56.2) | 11 (34.4) | 32 |  | 1 (4.4) | 2 (8.7) | 4 (17.4) | 3 (13.0) | 13 (56.5) | 23 |
|  |  |  | |  | |  | |  |  |  |  |  |  |  |  |  |  |  |  |
| Finland | 9 (18.8) | 29 (60.4) | | 10 (20.8) | | 48 | |  | 1 (25.0) | 1 (25.0) | 2 (50.0) | 4 |  | 10 (25.0) | 6 (15.0) | 12 (30.0) | 2 (5.0) | 10 (25.0) | 40 |
|  |  |  | |  | |  | |  |  |  |  |  |  |  |  |  |  |  |  |
| France | 8 (17.4) | 36 (78.3) | | 2 (4.3) | | 46 | |  | 1 (8.33) | 10 (83.3) | 1 (8.3) | 12 |  | 1 (3.7) | 6 (22.2) | 8 (29.6) | 3 (11.1) | 9 (33.3) | 27 |
|  |  |  | |  | |  | |  |  |  |  |  |  |  |  |  |  |  |  |
| Spain | 2 (12.5) | 13 (81.3) | | 1 (6.2) | | 16 | |  | 0 | 4 (100.0) | 0 | 4 |  | 1 (7.7) | 5 (38.5) | 6 (46.2) | 0 | 1 (7.7) | 13 |
|  |  |  | |  | |  | |  |  |  |  |  |  |  |  |  |  |  |  |
| Italy | 2 (16.7) | 10 (83.3) | | 0 | | 12 | |  | 0 | 4 (100.0) | 0 | 4 |  | 5 (25.0) | 6 (30.0) | 2 (10.0) | 3 (15.0) | 4 (20.0) | 20 |
|  |  |  | |  | |  | |  |  |  |  |  |  |  |  |  |  |  |  |
| Poland | 8 (36.4) | 13 (59.1) | | 1 (4.5) | | 22 | |  | 0 | 1 (100.0) | 0 | 1 |  | 0 | 4 (50.0) | 3 (37.5) | 1 (12.5) | 0 | 8 |
|  |  |  | |  | |  | |  |  |  |  |  |  |  |  |  |  |  |  |
| Iceland | 3 (21.4) | 10 (71.4) | | 1 (7.2) | | 14 | |  | 1 (33.3) | 1 (33.3) | 1 (33.3) | 3 |  | 1 (25.0) | 1 (25.0) | 0 | 1 (25.0) | 1 (25.0) | 4 |
|  |  |  | |  | |  | |  |  |  |  |  |  |  |  |  |  |  |  |
| United Kingdom | 3 (15.0) | 15 (75.0) | | 2 (10.0) | | 20 | |  | 0 | 0 | 0 | 0 |  | 6 (100.0) | 0 | 0 | 0 | 0 | 0 |
|  |  |  | |  | |  | |  |  |  |  |  |  |  |  |  |  |  |  |
| Republic of Ireland | 1 (11.1) | 6 (66.7) | | 2 (22.2) | | 9 | |  | 2 (50.0) | 2 (50.0) | 0 | 4 |  | 0 | 0 | 0 | 0 | 0 | 0 |
|  |  |  | |  | |  | |  |  |  |  |  |  |  |  |  |  |  |  |
| German | 0 | 4 (80.0) | | 1 (20.0) | | 5 | |  | 0 | 0 | 0 | 0 |  | 0 | 0 | 0 | 0 | 0 | 0 |
| Portugal | 0 | 0 | | 0 | | 0 | |  | 0 | 0 | 0 | 0 |  | 0 | 1 (50.0) | 1 (50.0) | 0 | 0 | 2 |
| **Adult’s development** | | | | | | | | | | | | | | | | | | | |
| Denmark | N/A | | | | | |  | | 28 (87.5) | 3 (9.4) | 1 (3.1) | 32 |  | 12 (52.2) | 4 (17.4) | 0 | 2 (8.7) | 5 (21.7) | 23 |
|  |  |  | |  | |  |  | |  |  |  |  |  |  |  |  |  |  |  |
| Finland | N/A | | | | | |  | | 4 (100.0) | 0 | 0 | 4 |  | 35 (87.5) | 3 (7.5) | 0 | 0 | 2 (5.0) | 40 |
|  |  |  | |  | |  |  | |  |  |  |  |  |  |  |  |  |  |  |
| France | N/A | | | | | |  | | 6 (50.0) | 6 (50.0) | 0 | 12 |  | 25 (92.6) | 0 | 1 (3.7) | 0 | 1 (3.7) | 27 |
|  |  |  | |  | |  |  | |  |  |  |  |  |  |  |  |  |  |  |
| Spain | N/A | | | | | |  | | 4 (100.0) | 0 | 0 | 4 |  | 11 (84.6) | 2 (15.4) | 0 | 0 | 0 | 13 |
|  |  |  | |  | |  |  | |  |  |  |  |  |  |  |  |  |  |  |
| Italy | N/A | | | | | |  | | 3 (75.0) | 1 (25.0) | 0 | 4 |  | 15 (75.0) | 2 (10.0) | 0 | 0 | 3 (15.0) | 20 |
|  |  |  | |  | |  |  | |  |  |  |  |  |  |  |  |  |  |  |
| Poland | N/A | | | | | |  | | 1 (100.0) | 0 | 0 | 1 |  | 5 (62.5) | 3 (37.5) | 0 | 0 | 0 | 8 |
|  |  |  | |  | |  |  | |  |  |  |  |  |  |  |  |  |  |  |
| Iceland | N/A | | | | | |  | | 3 (100.0) | 0 | 0 | 3 |  | 3 (75.0) | 1 (25.0) | 0 | 0 | 0 | 4 |
|  |  |  | |  | |  |  | |  |  |  |  |  |  |  |  |  |  |  |
| United Kingdom | N/A | | | | | |  | | 0 | 0 | 0 | 0 |  | 6 (100.0) | 0 | 0 | 0 | 0 | 6 |
|  |  |  | |  | |  |  | |  |  |  |  |  |  |  |  |  |  |  |
| Republic of Ireland | N/A | | | | | |  | | 3 (75.0) | 1 (25.0) | 0 | 4 |  | 0 | 0 | 0 | 0 | 0 | 0 |
|  |  |  | |  | |  |  | |  |  |  |  |  |  |  |  |  |  |  |
| German | N/A | | | | | |  | | 0 | 0 | 0 | 0 |  | 0 | 0 | 0 | 0 | 0 | 0 |
| Portugal | N/A | | | | | |  | | 0 | 0 | 0 | 0 |  | 1 (50.0) | 1 (50.0) | 0 | 0 | 0 | 2 |
| **Behavioral problems** | | | | | | | | | | | | | | | | | | | |
| Denmark | N/A | | | | | |  | | 30 (93.8) | 0 | 2 (6.2) | 32 |  | 18 (78.3) | 1 84.4) | 0 | 0 | 1 (5.0) | 23 |
|  |  |  | |  | |  |  | |  |  |  |  |  |  |  |  |  |  |  |
| Finland | N/A | | | | | |  | | 3 (75.0) | 0 | 1 (25.0) | 4 |  | 31 (77.5) | 5 (12.5) | 2 (5.0) | 0 | 2 (5.0) | 40 |
|  |  |  | |  | |  |  | |  |  |  |  |  |  |  |  |  |  |  |
| France | N/A | | | | | |  | | 10 (83.3) | 2 (16.7) | 0 | 12 |  | 21 (77.8) | 5 (18.5) | 0 | 0 | 1 (3.7) | 27 |
|  |  |  | |  | |  |  | |  |  |  |  |  |  |  |  |  |  |  |
| Spain | N/A | | | | | |  | | 4 (100.0) | 0 | 0 | 4 |  | 12 (92.3) | 0 | 1 (7.7) | 0 | 0 | 13 |
|  |  |  | |  | |  |  | |  |  |  |  |  |  |  |  |  |  |  |
| Italy | N/A | | | | | |  | | 4 (100.0) | 0 | 0 | 4 |  | 17 (85.0) | 2 (10.0) | 0 | 0 | 1 (5.0) | 20 |
|  |  |  | |  | |  |  | |  |  |  |  |  |  |  |  |  |  |  |
| Poland | N/A | | | | | |  | | 1 (100.0) | 0 | 0 | 1 |  | 5 (62.5) | 3 (37.5) | 0 | 0 | 0 | 8 |
|  |  |  | |  | |  |  | |  |  |  |  |  |  |  |  |  |  |  |
| Iceland | N/A | | | | | |  | | 2 (66.7) | 1 (33.3) | 0 | 3 |  | 3 (75.0) | 1 (25.0) | 0 | 0 | 0 | 4 |
|  |  |  | |  | |  |  | |  |  |  |  |  |  |  |  |  |  |  |
| United Kingdom | N/A | | | | | |  | | 0 | 0 | 0 | 0 |  | 6 (100.0) | 0 | 0 | 0 | 0 | 6 |
|  |  |  | |  | |  |  | |  |  |  |  |  |  |  |  |  |  |  |
| Republic of Ireland | N/A | | | | | |  | | 4 (100.0) | 0 | 0 | 4 |  | 0 | 0 | 0 | 0 | 0 | 0 |
|  |  |  | |  | |  |  | |  |  |  |  |  |  |  |  |  |  |  |
| German | N/A | | | | | |  | | 0 | 0 | 0 | 0 |  | 0 | 0 | 0 | 0 | 0 | 0 |
| Portugal | N/A | | | | | |  | | 0 | 0 | 0 | 0 |  | 2 (100.0) | 0 | 0 | 0 | 0 | 2 |
|  |  | | | | | |  | |  |  |  |  |  |  |  |  |  |  |  |
| **NOT recommended features for a routine evaluation** | | | | | | | | | | | | | | | | | | | |
| **Blood test for genetic studies** | | | | | | | | | | | | | | | | | | | |
| Denmark | 1 (0.7) | | 151 (97.4) | | 3 (1.9) | 155 |  | | 0 | 31 (96.9) | 1 (3.1) | 32 |  | 1 (4.4) | 4 (17.4) | 4 (17.4) | 3 (13.0) | 11 (47.8) | 23 |
|  |  | |  | |  |  |  | |  |  |  |  |  |  |  |  |  |  |  |
| Finland | 1 (2.1) | | 43 (89.6) | | 4 (8.3) | 48 |  | | 0 | 3 (75.0) | 1 (25.0) | 4 |  | 2 (5.0) | 6 (15.0) | 9 (22.5) | 12 (30.0) | 11 (27.5) | 40 |
|  |  | |  | |  |  |  | |  |  |  |  |  |  |  |  |  |  |  |
| France | 8 (17.4) | | 38 (82.6) | | 0 | 46 |  | | 1 (8.3) | 11 (91.7) | 0 | 12 |  | 7 (25.9) | 10 (37.0) | 4 (14.8) | 3 (11.1) | 3 (11.1) | 27 |
|  |  | |  | |  |  |  | |  |  |  |  |  |  |  |  |  |  |  |
| Spain | 2 (12.5) | | 14 (87.5) | | 0 | 16 |  | | 0 | 4 (100.0) | 0 | 4 |  | 1 (7.7) | 7 (53.9) | 4 (30.8) | 1 (7.7) | 0 | 13 |
|  |  | |  | |  |  |  | |  |  |  |  |  |  |  |  |  |  |  |
| Italy | 1 (8.3) | | 11 (91.7) | | 0 | 12 |  | | 1 (25.0) | 3 (75.0) | 0 | 4 |  | 8 (40.0) | 6 (30.0) | 4 (20.0) | 0 | 2 (10.0) | 20 |
|  |  | |  | |  |  |  | |  |  |  |  |  |  |  |  |  |  |  |
| Poland | 1 (4.6) | | 21 (95.4) | | 0 | 22 |  | | 0 | 1 (100.0) | 0 | 1 |  | 0 | 3 (37.5) | 5 (62.5) | 0 | 0 | 8 |
|  |  | |  | |  |  |  | |  |  |  |  |  |  |  |  |  |  |  |
| Iceland | 1 (7.1) | | 11 (78.6) | | 2 (14.3) | 14 |  | | 0 | 3 (100.0) | 0 | 3 |  | 0 | 1 (25.0) | 0 | 1 (25.0) | 2 (50.0) | 4 |
|  |  | |  | |  |  |  | |  |  |  |  |  |  |  |  |  |  |  |
| United Kingdom | 2 (10.0) | | 18 (90.0) | | 0 | 20 |  | | 0 | 0 | 0 | 0 |  | 0 | 0 | 3 (50.0) | 2 (33.3) | 1 (16.7) | 6 |
|  |  | |  | |  |  |  | |  |  |  |  |  |  |  |  |  |  |  |
| Republic of Ireland | 1 (11.1) | | 8 (88.9) | | 0 | 9 |  | | 0 | 4 (100.0) | 0 | 4 |  | 0 | 0 | 0 | 0 | 0 | 0 |
|  |  | |  | |  |  |  | |  |  |  |  |  |  |  |  |  |  |  |
| German | 0 | | 5 (100.0) | | 0 | 5 |  | | 0 | 0 | 0 | 0 |  | 0 | 0 | 0 | 0 | 0 | 0 |
|  |  | |  | |  |  |  | |  |  |  |  |  |  |  |  |  |  |  |
| Portugal | 0 | | 0 | | 0 | 0 |  | | 0 | 0 | 0 | 0 |  | 0 | 2 (100.0) | 0 | 0 | 0 | 2 |
| **Brain scan** | | | | | | | | | | | | | | | | | | | |
| Denmark | 3 (1.9) | | 149 (96.1) | | 3 (1.9) | 155 |  | | 1 (3.1) | 30 (93.8) | 1 (3.1) | 32 |  | 1 (4.4) | 4 (17.4) | 4 (17.4) | 3 (13.0) | 11 (47.8) | 23 |
|  |  | |  | |  |  |  | |  |  |  |  |  |  |  |  |  |  |  |
| Finland | 3 (6.2) | | 44 (91.7) | | 1 (2.1) | 48 |  | | 0 | 3 (75.0) | 1 (25.0) | 4 |  | 7 (17.5) | 5 (12.5) | 15 (37.5) | 4 (10.0) | 9 (22.5) | 40 |
|  |  | |  | |  |  |  | |  |  |  |  |  |  |  |  |  |  |  |
| France | 5 (10.9) | | 41 (89.1) | | 0 | 46 |  | | 1 (8.3) | 11 (91.7) | 0 | 12 |  | 4 (14.8) | 8 (29.6) | 7 (25.9) | 4 (14.8) | 4 (14.8) | 27 |
|  |  | |  | |  |  |  | |  |  |  |  |  |  |  |  |  |  |  |
| Spain | 2 (12.5) | | 14 (87.5) | | 0 | 16 |  | | 1 (25.0) | 3 (75.0) | 0 | 4 |  | 2 (15.4) | 6 (46.2) | 4 (30.8) | 1 (7.7) | 0 | 13 |
|  |  | |  | |  |  |  | |  |  |  |  |  |  |  |  |  |  |  |
| Italy | 1 (8.3) | | 11 (91.7) | | 0 | 12 |  | | 0 | 4 (100.0) | 0 | 4 |  | 7 (35.0) | 4 (20.0) | 2 (10.0) | 1 (5.0) | 6 (30.0) | 20 |
|  |  | |  | |  |  |  | |  |  |  |  |  |  |  |  |  |  |  |
| Poland | 4 (18.2) | | 18 (81.8) | | 0 | 22 |  | | 1 (100.0) | 0 | 0 | 1 |  | 2 (25.0) | 0 | 5 (62.5) | 1 (12.5) | 0 | 8 |
|  |  | |  | |  |  |  | |  |  |  |  |  |  |  |  |  |  |  |
| Iceland | 0 | | 14 (100.0) | | 0 | 14 |  | | 0 | 3 (100.0) | 0 | 3 |  | 1 (25.0) | 1 (25.0) | 0 | 0 | 2 (50.0) | 4 |
|  |  | |  | |  |  |  | |  |  |  |  |  |  |  |  |  |  |  |
| United Kingdom | 1 (5.0) | | 19 (95.0) | | 0 | 20 |  | | 0 | 0 | 0 | 0 |  | 0 | 0 | 3 (50.0) | 2 (33.3) | 1 (16.7) | 6 |
|  |  | |  | |  |  |  | |  |  |  |  |  |  |  |  |  |  |  |
| Republic of Ireland | 0 | | 9 (100.0) | | 0 | 9 |  | | 0 | 4 (100.0) | 0 | 4 |  | 0 | 0 | 0 | 0 | 0 | 0 |
|  |  | |  | |  |  |  | |  |  |  |  |  |  |  |  |  |  |  |
| German | 0 | | 5 (100.0) | | 0 | 5 |  | | 0 | 0 | 0 | 0 |  | 0 | 0 | 0 | 0 | 0 | 0 |
|  |  | |  | |  |  |  | |  |  |  |  |  |  |  |  |  |  |  |
| Portugal | 0 | | 0 | | 0 | 0 |  | | 0 | 0 | 0 | 0 |  | 0 | 2 (100.0) | 0 | 0 | 0 | 0 |

*Note*. Recommended and NOT recommended features were retrieved from the National Institute for Health and Care Excellence (2012). N/A=Question was not presented to the respondent group. The questions were the following: autistic adult=*Based on your experience, were the following factors part of your diagnostic evaluation for autism spectrum?*; Carer=*Were the following factors part of the adult's diagnostic evaluation for autism spectrum?*; Professional=*Thinking of the adult diagnostic service for autism spectrum that you know best, how often are the following factors part of the adult's diagnostic evaluation for autism spectrum in adults?.*

**Supplementary material 10**

*Recommended and NOT recommended features* for a routine adult diagnostic evaluation for autism spectrum. Analysis removing adult and carer respondents who reported the adult’s autism diagnosis at a time before the 2012 publication of NICE guidelines.

| Answer | Autistic adult (N=333) | | |  | Carer (N=58) | | |
| --- | --- | --- | --- | --- | --- | --- | --- |
|  | Recommended features | | | | | | |
|  | Yes | No | Do not know |  | Yes | No | Do not know |
|  | *n (%)* | | |  | *n (%)* | | |
| Multidisciplinary team | 147 (44.1) | 161 (48.4) | 25 (7.5) |  | 31 (53.5) | 26 (44.8) | 1 (1.7) |
|  |  |  |  |  |  |  |  |
| Close person asked about adult’s symptoms | 219 (65.8) | 108 (32.4) | 6 (1.8) |  | 46 (79.3) | 12 (20.7) | 0 |
|  |  |  |  |  |  |  |  |
| Questionnaire on symptoms | 235 (70.6) | 89 (26.7) | 9 (2.7) |  | N/A |  |  |
|  |  |  |  |  |  |  |  |
| Function evaluated in different settings | 300 (90.1) | 23 (6.9) | 10 (3.0) |  | 51 (87.9) | 7 (12.1) | 0 |
|  |  |  |  |  |  |  |  |
| Physical or mental problems | 282 (84.7) | 32 (9.6) | 19 (5.7) |  | 48 (82.8) | 7(12.1) | 3 (5.2) |
|  |  |  |  |  |  |  |  |
| Development problems like ADHD | 252 (75.7) | 55 (16.5) | 26 (7.8) |  | 50 (86.2) | 8 (13.8) | 0 |
|  |  |  |  |  |  |  |  |
| Language / communication difficulties | 286 (85.9) | 33 (9.9) | 14 (4.2) |  | 54 (93.1) | 4 (6.9) | 0 |
|  |  |  |  |  |  |  |  |
| Sensory problems | 275 (82.6) | 43 (12.9) | 15 (4.5) |  | 42 (72.4) | 11 (19.0) | 5 (8.6) |
|  |  |  |  |  |  |  |  |
| Self-harm or harm to others | 185 (55.6) | 107 (32.1) | 41 (12.3) |  | 43 (74.1) | 12 (20.7) | 3 (5.2) |
|  |  |  |  |  |  |  |  |
| Being abused / neglected or taken advantage of | 133 (39.9) | 157 (47.2) | 43 (12.9) |  | 26 (44.8) | 23 (39.7) | 9 (15.5) |
| Contact with the police | 63 (18.9) | 228 (68.5) | 42 (12.6) |  | 8 (13.8) | 37 (63.8) | 13 (22.4) |
| Adult’s development | N/A |  |  |  | 47 (81.0) | 10 (17.2) | 1 (1.8) |
|  |  |  |  |  |  |  |  |
| Behavioral problems | N/A |  |  |  | 53 (91.4) | 3 (5.2) | 2 (3.4) |
|  |  |  |  |  |  |  |  |
| Consultation with other experts | N/A |  |  |  | N/A |  |  |
|  |  |  |  |  |  |  |  |
| Core autistic behaviors | N/A |  |  |  | N/A |  |  |
|  |  |  |  |  |  |  |  |
| Direct observation | N/A |  |  |  | N/A |  |  |
|  |  |  |  |  |  |  |  |
| Standard tests for autism | N/A |  |  |  | N/A |  |  |
|  |  |  |  |  |  |  |  |
| Standard tests for cognitive or psychological functioning | N/A |  |  |  | N/A |  |  |
|  |  |  |  |  |  |  |  |
| Physical examination | N/A |  |  |  | N/A |  |  |
|  |  |  |  |  |  |  |  |
| NOT recommended features for a routine evaluation | | | | | | | |
| Blood test for genetic studies | 13 (3.9) | 312 (93.7) | 8 (2.4) |  | 2 (3.5) | 55 (94.8) | 1 (1.7) |
|  |  |  |  |  |  |  |  |
| Brain scan | 16 (4.8) | 313 (94.0) | 4 (1.2) |  | 4 (6.9) | 54 (93.1) | 0 |
|  |  |  |  |  |  |  |  |
| Biological tests | N/A |  |  |  | N/A |  |  |

*Note*. Recommended and NOT recommended features for a routine evaluation were retrieved from the National Institute for Health and Care Excellence (2012). N/A=Question was not presented to the respondent group. The questions were the following: autistic adult=*Based on your experience, were the following factors part of your diagnostic evaluation for autism spectrum?*; Carer=*Were the following factors part of the adult's diagnostic evaluation for autism spectrum?*; Professional=*Thinking of the adult diagnostic service for autism spectrum that you know best, how often are the following factors part of the adult's diagnostic evaluation for autism spectrum in adults?.*

**Supplementary material 11**

*Number of recommended features for autistic adult post-diagnosis support reported by responders*

| Number of recommendations | Autistic adult  (N=344) | Carer  (N=64) | Professional  (N=139) |
| --- | --- | --- | --- |
|  | *n (%)* | *n (%)* | *n (%)* |
| 0 | 229 (66.6) | 30 (46.9) | 23 (16.6) |
| 1 | 80 (23.3) | 15 (23.4) | 14 (10.1) |
| 2 | 27 (7.9) | 12 (18.8) | 17 (12.2) |
| 3 | 7 (2.0) | 5 (7.8) | 9 (6.5) |
| 4 | 1 (0.3) | 1 (1.6) | 14 (10.1) |
| 5 |  | 1 (1.6) | 33 (23.7) |
| 6 |  |  | 29 (20.9) |

**Supplementary material 12**

*Stratified results of information on recommended features for autistic adult post-diagnosis support by autistic by adults’ gender, high/some independence and high level of support/institution-like care of the autistic adult cared reported by carers*

| Answer | Autistic adult  *(N = 334)* | | | | | | | | | | | |  | Carer  *(N = 64)* | | | | | |
| --- | --- | --- | --- | --- | --- | --- | --- | --- | --- | --- | --- | --- | --- | --- | --- | --- | --- | --- | --- |
|  | Female | | Male | | Female | | Male | | Female | | | Male |  | High or some independence | High-level of support or institution-like care | High or some independence | High-level of support or institution-like care | High or some independence | High-level of support or institution-like care |
|  | Yes | | | | No | | | | | Do not know | | |  | Yes | | No | | Do not know | |
| Written recommendations for care and follow-up for non-medical problems | 45 (17.6) | | 18 (23.1) | | 196  (76.6) | | 50  (64.1) | | | 15  (5.9) | | 10  (12.8) |  | 19  (42.2) | 3  (15.8) | 23  (51.1) | 15  (78.9) | 3  (6.7) | 1  (5.3) |
|  |  | |  | |  | |  | | |  | |  |  |  |  |  |  |  |  |
| Written recommendations for health care | 39 (15.2) | | 12 (15.4) | | 199  (77.7) | | 60  (76.9) | | | 18  (7.0) | | 6  (7.7) |  | 6  (13.3) | 2  (10.5) | 35  (77.8) | 15  (78.9) | 4  (8.9) | 2  (10.5) |
|  |  | |  | |  | |  | | |  | |  |  |  |  |  |  |  |  |
| ‘Health passport' to carry important information about the adult needs and care | 2  (0.8) | | 3  (3.9) | | 134  (91.4) | | 70  (89.8) | | | 20  (7.8) | | 5  (6.4) |  | 4  (8.9) | 1  (5.3) | 37  (82.2) | 17  (89.5) | 4  (8.9) | 1  (5.3) |
|  |  | |  | |  | |  | | |  | |  |  |  |  |  |  |  |  |
| Referral for specialist care for health or medical problems | 28 (10.9) | | 12 (15.4) | | 209  (81.6) | | 61  (78.2) | | | 19  (7.4) | | 5  (6.4) |  | 17  (37.8) | 3  (15.8) | 26  (57.8) | 16  (84.2) | 2  (4.4) | 0  (0.0) |
|  |  |  | |  | |  | |  | | |  | |  |  |  |  |  |  |  |
| Written recommendations for how to manage a crisis | N/A | | | | | | | | | | | |  | 3  (6.7) | 2  (10.5) | 37  (82.2) | 15  (78.9) | 5  (11.1) | 2  (10.5) |
|  |  | | | | | | | | | | | |  |  |  |  |  |  |  |
| Written recommendations for managing risks | N/A | | | | | | | | | | | |  | 3  (6.7) | 0  (0.0) | 31  (68.9) | 18  (94.7) | 11  (24.4) | 1  (5.3) |

*Note*. N/A = Question was not presented to the respondent group. *No, but it was needed* and *No, and it was NOT needed* are considered *as* NO. The original questions were the following: autistic adult = *After you got the autism spectrum diagnosis, which of the following things happened?*; Carer = *After the adult got the autism spectrum diagnosis, which of the following things happened?*; Professional = *Thinking of the adult diagnostic service for autism spectrum that you know best, how often are the following factors considered as parts of the post-diagnostic activities for autistic adults?* Values expressed as number of responders and frequencies (in parenthesis).

**Supplementary material 13**

*Recommended features for autistic adult post-diagnosis support by countries*

| Answer | | | | |  | | | | | | Autistic adult  (N=344) | | | | | | | | | | | | | | | | | | | | | | | | | | | | |  | | | | | | | | | | Carer  (N=64) | | | | | | | | | | | | | | | | | | | | | | | | | | | | | | | | | | | | | | | | | | | | | | | | | | | | |  | | | | | | | | | | Professional (N=139) | | | | | | | | | | | | | | | | | | | | | | | | | | | | | | | | | | | | | | | | | | | | | | | | | | | | |
| --- | --- | --- | --- | --- | --- | --- | --- | --- | --- | --- | --- | --- | --- | --- | --- | --- | --- | --- | --- | --- | --- | --- | --- | --- | --- | --- | --- | --- | --- | --- | --- | --- | --- | --- | --- | --- | --- | --- | --- | --- | --- | --- | --- | --- | --- | --- | --- | --- | --- | --- | --- | --- | --- | --- | --- | --- | --- | --- | --- | --- | --- | --- | --- | --- | --- | --- | --- | --- | --- | --- | --- | --- | --- | --- | --- | --- | --- | --- | --- | --- | --- | --- | --- | --- | --- | --- | --- | --- | --- | --- | --- | --- | --- | --- | --- | --- | --- | --- | --- | --- | --- | --- | --- | --- | --- | --- | --- | --- | --- | --- | --- | --- | --- | --- | --- | --- | --- | --- | --- | --- | --- | --- | --- | --- | --- | --- | --- | --- | --- | --- | --- | --- | --- | --- | --- | --- | --- | --- | --- | --- | --- | --- | --- | --- | --- | --- | --- | --- | --- | --- | --- | --- | --- | --- | --- | --- | --- | --- | --- | --- | --- | --- | --- | --- | --- |
|  |  |  |  |  | Yes | | | | | No, but it was needed | | | | | | | No and it was not needed | | | | | | | | | Do not know | | | | | Total | | | | | | |  | | | | | | | | | | Yes | | | | | | | | | | | | No, but it was needed | | | | | | | | | No and it was not needed | | | | | | | | | | Do not know | | | | | | | | | | Total | | | | | | | | | | | | | | |  | | | | | | | | | | | Standard routine practice | | | | | | | | | Not standard practice, but often considered | | | | | | | | | | Rarely considered | | | | | | | | | | Never considered | | | | | | | | | | Do not know | | | | | | | | | | Total | |
|  |  |  |  |  | *n (%)* | | | | | | | | | | | | | | | | | | | | | | | | | | *N* | | | | | | |  | | | | | | | | | | *n (%)* | | | | | | | | | | | | | | | | | | | | | | | | | | | | | | | | | | | | | | | | | *N* | | | | | | | | | | | | | | |  | | | | | | | | | | | *n (%)* | | | | | | | | | | | | | | | | | | | | | | | | | | | | | | | | | | | | | | | | | | | | | | | | | *N* | |
| **Written recommendations for care and follow-up for non-medical problems** | | | | | | | | | | | | | | | | | | | | | | | | | | | | | | | | | | | | | | | | | | | | | | | | | | | | | | | | | | | | | | | | | | | | | | | | | | | | | | | | | | | | | | | | | | | | | | | | | | | | | | | | | | | | | | | | | | | | | | | | | | | | | | | | | | | | | | | | | | | | | | | | | | | | | | | | | | | | | | | | | | | | | |
| Denmark | | | | | 26 (16.9) | | | | | 94 (61.0) | | | | | | | 22 (14.3) | | | | | | | | | 12 (7.8) | | | | | 154 | | | | | | |  | | | | | | | | | | 8 (25.0) | | | | | | | | | | | | 20 (62.5) | | | | | | | | | 1 (3.1) | | | | | | | | | | 3 (9.4) | | | | | | | | | | 32 | | | | | | | | | | | | | | |  | | | | | | | | | | | 10 (47.6) | | | | | | | | | 3 (14.3) | | | | | | | | | | 1 (4.8) | | | | | | | | | | 0 | | | | | | | | | | 7 (33.3) | | | | | | | | | | 21 | |
|  | | | | |  | | | | |  | | | | | | |  | | | | | | | | |  | | | | |  | | | | | | |  | | | | | | | | | |  | | | | | | | | | | | |  | | | | | | | | |  | | | | | | | | | |  | | | | | | | | | |  | | | | | | | | | | | | | | |  | | | | | | | | | | |  | | | | | | | | |  | | | | | | | | | |  | | | | | | | | | |  | | | | | | | | | |  | | | | | | | | | |  | |
| Finland | | | | | 3 (6.2) | | | | | 36 (75.0) | | | | | | | 8 (16.7) | | | | | | | | | 1 (2.1) | | | | | 48 | | | | | | |  | | | | | | | | | | 0 | | | | | | | | | | | | 3 (75.0) | | | | | | | | | 0 | | | | | | | | | | 1 (25.0) | | | | | | | | | | 4 | | | | | | | | | | | | | | |  | | | | | | | | | | | 9 (23.1) | | | | | | | | | 7 (18.0) | | | | | | | | | | 8 (20.5) | | | | | | | | | | 2 (5.1) | | | | | | | | | | 13 (33.3) | | | | | | | | | | 39 | |
|  | | | | |  | | | | |  | | | | | | |  | | | | | | | | |  | | | | |  | | | | | | |  | | | | | | | | | |  | | | | | | | | | | | |  | | | | | | | | |  | | | | | | | | | |  | | | | | | | | | |  | | | | | | | | | | | | | | |  | | | | | | | | | | |  | | | | | | | | |  | | | | | | | | | |  | | | | | | | | | |  | | | | | | | | | |  | | | | | | | | | |  | |
| France | | | | | 12 (26.7) | | | | | 19 (42.2) | | | | | | | 10 (22.2) | | | | | | | | | 4 (8.9) | | | | | 45 | | | | | | |  | | | | | | | | | | 6 (50.0) | | | | | | | | | | | | 5 (41.7) | | | | | | | | | 1 (8.3) | | | | | | | | | | 0 | | | | | | | | | | 12 | | | | | | | | | | | | | | |  | | | | | | | | | | | 22 (81.5) | | | | | | | | | 2 (7.4) | | | | | | | | | | 0 | | | | | | | | | | 0 | | | | | | | | | | 3 (11.1) | | | | | | | | | | 27 | |
|  | | | | |  | | | | |  | | | | | | |  | | | | | | | | |  | | | | |  | | | | | | |  | | | | | | | | | |  | | | | | | | | | | | |  | | | | | | | | |  | | | | | | | | | |  | | | | | | | | | |  | | | | | | | | | | | | | | |  | | | | | | | | | | |  | | | | | | | | |  | | | | | | | | | |  | | | | | | | | | |  | | | | | | | | | |  | | | | | | | | | |  | |
| Spain | | | | | 4 (25.0) | | | | | 5 (31.2) | | | | | | | 7 (43.8) | | | | | | | | | 0 | | | | | 16 | | | | | | |  | | | | | | | | | | 3 (75.0) | | | | | | | | | | | | 1 (25.0) | | | | | | | | | 0 | | | | | | | | | | 0 | | | | | | | | | | 4 | | | | | | | | | | | | | | |  | | | | | | | | | | | 6 (46.2) | | | | | | | | | 6 (46.2) | | | | | | | | | | 1 (7.7) | | | | | | | | | | 0 | | | | | | | | | | 0 | | | | | | | | | | 13 | |
|  | | | | |  | | | | |  | | | | | | |  | | | | | | | | |  | | | | |  | | | | | | |  | | | | | | | | | |  | | | | | | | | | | | |  | | | | | | | | |  | | | | | | | | | |  | | | | | | | | | |  | | | | | | | | | | | | | | |  | | | | | | | | | | |  | | | | | | | | |  | | | | | | | | | |  | | | | | | | | | |  | | | | | | | | | |  | | | | | | | | | |  | |
| Italy | | | | | 2 (16.7) | | | | | 1 (8.3) | | | | | | | 6 (50.0) | | | | | | | | | 3 (25.0) | | | | | 12 | | | | | | |  | | | | | | | | | | 2 (50.0) | | | | | | | | | | | | 2 (50.0) | | | | | | | | | 0 | | | | | | | | | | 0 | | | | | | | | | | 4 | | | | | | | | | | | | | | |  | | | | | | | | | | | 11 (57.9) | | | | | | | | | 5 (26.3) | | | | | | | | | | 1 (5.3) | | | | | | | | | | 1 (5.3) | | | | | | | | | | 1 (5.3) | | | | | | | | | | 19 | |
|  | | | | |  | | | | |  | | | | | | |  | | | | | | | | |  | | | | |  | | | | | | |  | | | | | | | | | |  | | | | | | | | | | | |  | | | | | | | | |  | | | | | | | | | |  | | | | | | | | | |  | | | | | | | | | | | | | | |  | | | | | | | | | | |  | | | | | | | | |  | | | | | | | | | |  | | | | | | | | | |  | | | | | | | | | |  | | | | | | | | | |  | |
| Poland | | | | | 4 (18.2) | | | | | 7 (31.8) | | | | | | | 11 (50.0) | | | | | | | | | 0 | | | | | 22 | | | | | | |  | | | | | | | | | | 1 (100.0) | | | | | | | | | | | | 0 | | | | | | | | | 0 | | | | | | | | | | 0 | | | | | | | | | | 1 | | | | | | | | | | | | | | |  | | | | | | | | | | | 5 (62.5) | | | | | | | | | 3 (37.5) | | | | | | | | | | 0 | | | | | | | | | | 0 | | | | | | | | | | 0 | | | | | | | | | | 8 | |
|  | | | | |  | | | | |  | | | | | | |  | | | | | | | | |  | | | | |  | | | | | | |  | | | | | | | | | |  | | | | | | | | | | | |  | | | | | | | | |  | | | | | | | | | |  | | | | | | | | | |  | | | | | | | | | | | | | | |  | | | | | | | | | | |  | | | | | | | | |  | | | | | | | | | |  | | | | | | | | | |  | | | | | | | | | |  | | | | | | | | | |  | |
| Iceland | | | | | 0 | | | | | 7 (53.8) | | | | | | | 5 (38.5) | | | | | | | | | 1 (7.7) | | | | | 13 | | | | | | |  | | | | | | | | | | 0 | | | | | | | | | | | | 2 (66.7) | | | | | | | | | 1 (33.3) | | | | | | | | | | 0 | | | | | | | | | | 3 | | | | | | | | | | | | | | |  | | | | | | | | | | | 1 (25.0) | | | | | | | | | 0 | | | | | | | | | | 1 (25.0) | | | | | | | | | | 1 (25.0) | | | | | | | | | | 1 (25.0) | | | | | | | | | | 4 | |
|  | | | | |  | | | | |  | | | | | | |  | | | | | | | | |  | | | | |  | | | | | | |  | | | | | | | | | |  | | | | | | | | | | | |  | | | | | | | | |  | | | | | | | | | |  | | | | | | | | | |  | | | | | | | | | | | | | | |  | | | | | | | | | | |  | | | | | | | | |  | | | | | | | | | |  | | | | | | | | | |  | | | | | | | | | |  | | | | | | | | | |  | |
| United Kingdom | | | | | 4 (20.0) | | | | | 11 (55.0) | | | | | | | 2 (10.0) | | | | | | | | | 3 (15.0) | | | | | 20 | | | | | | |  | | | | | | | | | | 0 | | | | | | | | | | | | 0 | | | | | | | | | 0 | | | | | | | | | | 0 | | | | | | | | | | 0 | | | | | | | | | | | | | | |  | | | | | | | | | | | 6 (100.0) | | | | | | | | | 0 | | | | | | | | | | 0 | | | | | | | | | | 0 | | | | | | | | | | 0 | | | | | | | | | | 0 | |
|  | | | | |  | | | | |  | | | | | | |  | | | | | | | | |  | | | | |  | | | | | | |  | | | | | | | | | |  | | | | | | | | | | | |  | | | | | | | | |  | | | | | | | | | |  | | | | | | | | | |  | | | | | | | | | | | | | | |  | | | | | | | | | | |  | | | | | | | | |  | | | | | | | | | |  | | | | | | | | | |  | | | | | | | | | |  | | | | | | | | | |  | |
| Republic of Ireland | | | | | 6 (66.7) | | | | | 1 (11.1) | | | | | | | 0 | | | | | | | | | 2 (22.2) | | | | | 9 | | | | | | |  | | | | | | | | | | 2 (50.0) | | | | | | | | | | | | 2 (50.0) | | | | | | | | | 0 | | | | | | | | | | 0 | | | | | | | | | | 4 | | | | | | | | | | | | | | |  | | | | | | | | | | | 0 | | | | | | | | | 0 | | | | | | | | | | 0 | | | | | | | | | | 0 | | | | | | | | | | 0 | | | | | | | | | | 0 | |
|  | | | | |  | | | | |  | | | | | | |  | | | | | | | | |  | | | | |  | | | | | | |  | | | | | | | | | |  | | | | | | | | | | | |  | | | | | | | | |  | | | | | | | | | |  | | | | | | | | | |  | | | | | | | | | | | | | | |  | | | | | | | | | | |  | | | | | | | | |  | | | | | | | | | |  | | | | | | | | | |  | | | | | | | | | |  | | | | | | | | | |  | |
| German | | | | | 2 (40.0) | | | | | 1 (20.0) | | | | | | | 2 (40.0) | | | | | | | | | 0 | | | | | 5 | | | | | | |  | | | | | | | | | | 0 | | | | | | | | | | | | 0 | | | | | | | | | 0 | | | | | | | | | | 0 | | | | | | | | | | 0 | | | | | | | | | | | | | | |  | | | | | | | | | | | 0 | | | | | | | | | 0 | | | | | | | | | | 0 | | | | | | | | | | 0 | | | | | | | | | | 0 | | | | | | | | | | 0 | |
| Portugal | | | | | 0 | | | | | 0 | | | | | | | 0 | | | | | | | | | 0 | | | | | 0 | | | | | | |  | | | | | | | | | | 0 | | | | | | | | | | | | 0 | | | | | | | | | 0 | | | | | | | | | | 0 | | | | | | | | | | 0 | | | | | | | | | | | | | | |  | | | | | | | | | | | 2 (100.0) | | | | | | | | | 0 | | | | | | | | | | 0 | | | | | | | | | | 0 | | | | | | | | | | 0 | | | | | | | | | | 0 | |
| **Written recommendations for health care** | | | | | | | | | | | | | | | | | | | | | | | | | | | | | | | | | | | | | | | | | | | | | | | | | | | | | | | | | | | | | | | | | | | | | | | | | | | | | | | | | | | | | | | | | | | | | | | | | | | | | | | | | | | | | | | | | | | | | | | | | | | | | | | | | | | | | | | | | | | | | | | | | | | | | | | | | | | | | | | | | | | | | |
| Denmark | | | | | 18 (11.7) | | | | | 89 (57.8) | | | | | | | 34 (22.1) | | | | | | | | | 13 (8.4) | | | | | 154 | | | | | | |  | | | | | | | | | | 3 (9.4) | | | | | | | | | | | | 21 (65.6) | | | | | | | | | 5 (15.6) | | | | | | | | | | 3 (9.4) | | | | | | | | | | 32 | | | | | | | | | | | | | |  | | | | | | | | | | 6 (28.6) | | | | | | | | | | 2 (9.5) | | | | | | | | | | 2 (9.5) | | | | | | | | | | 0 | | | | | | | | | | 11 (52.3) | | | | | | | | | | 21 | | |
|  | | | | |  | | | | |  | | | | | | |  | | | | | | | | |  | | | | |  | | | | | | |  | | | | | | | | | |  | | | | | | | | | | | |  | | | | | | | | |  | | | | | | | | | |  | | | | | | | | | |  | | | | | | | | | | | | | |  | | | | | | | | | |  | | | | | | | | | |  | | | | | | | | | |  | | | | | | | | | |  | | | | | | | | | |  | | | | | | | | | |  | | |
| Finland | | | | | 6 (12.5) | | | | | 33 (68.7) | | | | | | | 7 (14.6) | | | | | | | | | 2 (4.2) | | | | | 48 | | | | | | |  | | | | | | | | | | 0 | | | | | | | | | | | | 1 (25.0) | | | | | | | | | 0 | | | | | | | | | | 3 (75.0) | | | | | | | | | | 4 | | | | | | | | | | | | | |  | | | | | | | | | | 7 (17.9) | | | | | | | | | | 13 (33.3) | | | | | | | | | | 8 (20.5) | | | | | | | | | | 2 (5.1) | | | | | | | | | | 9 (23.1) | | | | | | | | | | 39 | | |
|  | | | | |  | | | | |  | | | | | | |  | | | | | | | | |  | | | | |  | | | | | | |  | | | | | | | | | |  | | | | | | | | | | | |  | | | | | | | | |  | | | | | | | | | |  | | | | | | | | | |  | | | | | | | | | | | | | |  | | | | | | | | | |  | | | | | | | | | |  | | | | | | | | | |  | | | | | | | | | |  | | | | | | | | | |  | | | | | | | | | |  | | |
| France | | | | | 11 (24.4) | | | | | 19 (42.2) | | | | | | | 13 (28.9) | | | | | | | | | 2 (4.4) | | | | | 45 | | | | | | |  | | | | | | | | | | 3 (25.0) | | | | | | | | | | | | 6 (50.0) | | | | | | | | | 3 (25.0) | | | | | | | | | | 0 | | | | | | | | | | 12 | | | | | | | | | | | | | |  | | | | | | | | | | 16 (56.3) | | | | | | | | | | 7 825.9) | | | | | | | | | | 0 | | | | | | | | | | 0 | | | | | | | | | | 4 (14.8) | | | | | | | | | | 27 | | |
|  | | | | |  | | | | |  | | | | | | |  | | | | | | | | |  | | | | |  | | | | | | |  | | | | | | | | | |  | | | | | | | | | | | |  | | | | | | | | |  | | | | | | | | | |  | | | | | | | | | |  | | | | | | | | | | | | | |  | | | | | | | | | |  | | | | | | | | | |  | | | | | | | | | |  | | | | | | | | | |  | | | | | | | | | |  | | | | | | | | | |  | | |
| Spain | | | | | 1 (6.2) | | | | | 7 (43.8) | | | | | | | 6 (37.5) | | | | | | | | | 2 (12.5) | | | | | 16 | | | | | | |  | | | | | | | | | | 0 | | | | | | | | | | | | 2 (50.0) | | | | | | | | | 2 (50.0) | | | | | | | | | | 0 | | | | | | | | | | 4 | | | | | | | | | | | | | |  | | | | | | | | | | 4 (30.7) | | | | | | | | | | 7 (53.9) | | | | | | | | | | 2 815.4) | | | | | | | | | | 0 | | | | | | | | | | 0 | | | | | | | | | | 13 | | |
|  | | | | |  | | | | |  | | | | | | |  | | | | | | | | |  | | | | |  | | | | | | |  | | | | | | | | | |  | | | | | | | | | | | |  | | | | | | | | |  | | | | | | | | | |  | | | | | | | | | |  | | | | | | | | | | | | | |  | | | | | | | | | |  | | | | | | | | | |  | | | | | | | | | |  | | | | | | | | | |  | | | | | | | | | |  | | | | | | | | | |  | | |
| Italy | | | | | 1 (8.3) | | | | | 1 (8.3) | | | | | | | 9 (75.0) | | | | | | | | | 1 (8.3) | | | | | 12 | | | | | | |  | | | | | | | | | | 0 | | | | | | | | | | | | 2 (50.0) | | | | | | | | | 2 (50.0) | | | | | | | | | | 0 | | | | | | | | | | 4 | | | | | | | | | | | | | |  | | | | | | | | | | 9 (47.4) | | | | | | | | | | 6 (31.6) | | | | | | | | | | 2 (10.5) | | | | | | | | | | 0 | | | | | | | | | | 2 (10.5) | | | | | | | | | | 19 | | |
|  | | | | |  | | | | |  | | | | | | |  | | | | | | | | |  | | | | |  | | | | | | |  | | | | | | | | | |  | | | | | | | | | | | |  | | | | | | | | |  | | | | | | | | | |  | | | | | | | | | |  | | | | | | | | | | | | | |  | | | | | | | | | |  | | | | | | | | | |  | | | | | | | | | |  | | | | | | | | | |  | | | | | | | | | |  | | | | | | | | | |  | | |
| Poland | | | | | 4 (18.2) | | | | | 7 (31.8) | | | | | | | 11 (50.0) | | | | | | | | | 0 | | | | | 22 | | | | | | |  | | | | | | | | | | 1 (100.0) | | | | | | | | | | | | 0 | | | | | | | | | 0 | | | | | | | | | | 0 | | | | | | | | | | 1 | | | | | | | | | | | | | |  | | | | | | | | | | 1 (12.5) | | | | | | | | | | 6 (75.0) | | | | | | | | | | 1 (12.5) | | | | | | | | | | 0 | | | | | | | | | | 0 | | | | | | | | | | 8 | | |
|  | | | | |  | | | | |  | | | | | | |  | | | | | | | | |  | | | | |  | | | | | | |  | | | | | | | | | |  | | | | | | | | | | | |  | | | | | | | | |  | | | | | | | | | |  | | | | | | | | | |  | | | | | | | | | | | | | |  | | | | | | | | | |  | | | | | | | | | |  | | | | | | | | | |  | | | | | | | | | |  | | | | | | | | | |  | | | | | | | | | |  | | |
| Iceland | | | | | 2 (15.4) | | | | | 6 (46.1) | | | | | | | 4 (30.8) | | | | | | | | | 1 (7.7) | | | | | 13 | | | | | | |  | | | | | | | | | | 0 | | | | | | | | | | | | 2 (66.7) | | | | | | | | | 1 (33.3) | | | | | | | | | | 0 | | | | | | | | | | 3 | | | | | | | | | | | | | |  | | | | | | | | | | 0 | | | | | | | | | | 0 | | | | | | | | | | 1 (25.0) | | | | | | | | | | 1 (25.0) | | | | | | | | | | 2 (50.0) | | | | | | | | | | 4 | | |
|  | | | | |  | | | | |  | | | | | | |  | | | | | | | | |  | | | | |  | | | | | | |  | | | | | | | | | |  | | | | | | | | | |  | | | | | | | | | |  | | | | | | | | |  | | | | | | | | | |  | | | | | | | | | | | | | |  | | | | | | | | |  | | | | | | | | | | |  | | | | | | | | | |  | | | | | | | | | |  | | | | | | | | | |  | | | | | | | | |  | | | | | |
| United Kingdom | | | | | 2 (10.0) | | | | | 10 (50.0) | | | | | | | 7 (35.0) | | | | | | | | | 1 | | | | | 20 | | | | | | |  | | | | | | | | | | 0 | | | | | | | | | | 0 | | | | | | | | | | 0 | | | | | | | | | 0 | | | | | | | | | | 0 | | | | | | | | | | | | | |  | | | | | | | | | 4 (66.7) | | | | | | | | | | | 1 (16.7) | | | | | | | | | | 1 (16.7) | | | | | | | | | | 0 | | | | | | | | | | 0 | | | | | | | | | 6 | | | | | |
|  | | | | |  | | | | |  | | | | | | |  | | | | | | | | |  | | | | |  | | | | | | |  | | | | | | | | | |  | | | | | | | | | |  | | | | | | | | | |  | | | | | | | | |  | | | | | | | | | |  | | | | | | | | | | | | | |  | | | | | | | | |  | | | | | | | | | | |  | | | | | | | | | |  | | | | | | | | | |  | | | | | | | | | |  | | | | | | | | |  | | | | | |
| Republic of Ireland | | 4 (44.4) | | | | | | | 1 (11.1) | | | | | | | 2 (22.2) | | | | | | | 2 (22.2) | | | | | | | 9 | | | | |  | | | | | | | | | | | 1 (25.0) | | | | | | | | | | 3 (75.0) | | | | | | | | | | 0 | | | | | | | | | 0 | | | | | | | | | | 4 | | | | | | | | | | | | | |  | | | | | | | | | 0 | | | | | | | | | | | 0 | | | | | | | | | | 0 | | | | | | | | | | | | 0 | | | | | | | | | | 0 | | | | | | | | | | 0 | | | | |
|  |  | | | | | | |  | | | | | | |  | | | | | |  | | | | | | |  | | | | | |  | | | | | | | | | | |  | | | | | | | | | |  | | | | | | | | | |  | | | | | | | | |  | | | | | | | | | |  | | | | | | | | | | | | | |  | | | | | | | | |  | | | | | | | | | | |  | | | | | | | | | |  | | | | | | | | | | |  | | | | | | | | | |  | | | | | | | | |  | | | | | | | |
| German | 2 (40.0) | | | | | | | 2 (40.0) | | | | | | | 1 (20.0) | | | | | | 0 | | | | | | | 5 | | | | | |  | | | | | | | | | | | 0 | | | | | | | | | | 0 | | | | | | | | | | 0 | | | | | | | | | 0 | | | | | | | | | | 0 | | | | | | | | | | | | | |  | | | | | | | | | 0 | | | | | | | | | | | 0 | | | | | | | | | | 0 | | | | | | | | | | | 0 | | | | | | | | | | 0 | | | | | | | | | 0 | | | | | | | |
| Portugal | 0 | | | | | | | 0 | | | | | | | 0 | | | | | | 0 | | | | | | | 0 | | | | | |  | | | | | | | | | | | 0 | | | | | | | | | | 0 | | | | | | | | | | 0 | | | | | | | | | 0 | | | | | | | | | | 0 | | | | | | | | | | | | | |  | | | | | | | | | 0 | | | | | | | | | | | 2 (100.0) | | | | | | | | | | 0 | | | | | | | | | | | 0 | | | | | | | | | | 0 | | | | | | | | | 2 | | | | | | | |
|  |  | | | | | | |  | | | | | | |  | | | | | |  | | | | | | |  | | | | | |  | | | | | | | | | | |  | | | | | | | | | |  | | | | | | | | | |  | | | | | | | | |  | | | | | | | | | |  | | | | | | | | | | | | | |  | | | | | | | | |  | | | | | | | | | | |  | | | | | | | | | |  | | | | | | | | | | |  | | | | | | | | | |  | | | | | | | | |  | | | | | | | |
| **Health passport to carry important information about the adult needs and care** | | | | | | | | | | | | | | | | | | | | | | | | | | | | | | | | | | | | | | | | | | | | | | | | | | | | | | | | | | | | | | | | | | | | | | | | | | | | | | | | | | | | | | | | | | | | | | | | | | | | | | | | | | | | | | | | | | | | | | | | | | | | | | | | | | | | | | | | | | | | | | | | | | | | | | | | | | | | | | | | | | | | | |
| Denmark | 1 (0.6) | | | | | | | 99 (64.3) | | | | | | | 40 (26.0) | | | | | | | 14 (9.1) | | | | | | 154 | | | | | |  | | | | | | | | | | | 2 (6.2) | | | | | | | | | | 26 (81.3) | | | | | | | | | | 2 (6.2) | | | | | | | | | 2 (6.2) | | | | | | | | | | 32 | | | | | | | | | | | | | |  | | | | | | | | | 1 (4.8) | | | | | | | | | | | 2 (9.5) | | | | | | | | | | 3 (14.3) | | | | | | | | | | | 2 (9.5) | | | | | | | | | | 13 (61.9) | | | | | | | | | 21 | | | | | | | |
|  |  | | | | | | |  | | | | | | |  | | | | | | |  | | | | | |  | | | | | |  | | | | | | | | | | |  | | | | | | | | | |  | | | | | | | | | |  | | | | | | | | |  | | | | | | | | | |  | | | | | | | | | | | | | |  | | | | | | | | |  | | | | | | | | | | |  | | | | | | | | | |  | | | | | | | | | | |  | | | | | | | | | |  | | | | | | | | |  | | | | | | | |
| Finland | 0 | | | | | | | 33 (68.7) | | | | | | | 13 (27.1) | | | | | | | 2 (4.2) | | | | | | 48 | | | | | |  | | | | | | | | | | | 1 (25.0) | | | | | | | | | | 2 (50.0) | | | | | | | | | | 0 | | | | | | | | | 1 (25.0) | | | | | | | | | | 4 | | | | | | | | | | | | | |  | | | | | | | | | 1 (2.6) | | | | | | | | | | | 4 (10.3) | | | | | | | | | | 10 (25.6) | | | | | | | | | | | 9 (23.1) | | | | | | | | | | 15 (38.5) | | | | | | | | | 39 | | | | | | | |
|  |  | | | | | | |  | | | | | | |  | | | | | | |  | | | | | |  | | | | | |  | | | | | | | | | | |  | | | | | | | | | |  | | | | | | | | | |  | | | | | | | | |  | | | | | | | | | |  | | | | | | | | | | | | | |  | | | | | | | | |  | | | | | | | | | | |  | | | | | | | | | |  | | | | | | | | | | |  | | | | | | | | | |  | | | | | | | | |  | | | | | | | |
| France | 2 (4.4) | | | | | | | 32 (71.1) | | | | | | | 8 (17.8) | | | | | | | 3 (6.7) | | | | | | 45 | | | | | |  | | | | | | | | | | | 1 (8.3) | | | | | | | | | | 8 (66.7) | | | | | | | | | | 1 (8.3) | | | | | | | | | 2 (16.7) | | | | | | | | | | 12 | | | | | | | | | | | | | |  | | | | | | | | | 2 (7.4) | | | | | | | | | | | 6 (22.2) | | | | | | | | | | 5 (18.5) | | | | | | | | | | | 11 (40.7) | | | | | | | | | | 3 (11.1) | | | | | | | | | 27 | | | | | | | |
|  |  | | | | | | |  | | | | | | |  | | | | | | |  | | | | | |  | | | | | |  | | | | | | | | | | |  | | | | | | | | | |  | | | | | | | | | |  | | | | | | | | |  | | | | | | | | | |  | | | | | | | | | | | | | |  | | | | | | | | |  | | | | | | | | | | |  | | | | | | | | | |  | | | | | | | | | | |  | | | | | | | | | |  | | | | | | | | |  | | | | | | | |
| Spain | 0 | | | | | | | 10 (62.5) | | | | | | | 5 (31.2) | | | | | | | 1 (6.3) | | | | | | 16 | | | | | |  | | | | | | | | | | | 0 | | | | | | | | | | 0 | | | | | | | | | | 4 (100.0) | | | | | | | | | 0 | | | | | | | | | | 4 | | | | | | | | | | | | | |  | | | | | | | | | 2 (15.4) | | | | | | | | | | | 2 (15.4) | | | | | | | | | | 6 (46.2) | | | | | | | | | | | 3 (23.1) | | | | | | | | | | 0 | | | | | | | | | 13 | | | | | | | |
|  |  | | | | | | |  | | | | | | |  | | | | | | |  | | | | | |  | | | | | |  | | | | | | | | | | |  | | | | | | | | | |  | | | | | | | | | |  | | | | | | | | |  | | | | | | | | | |  | | | | | | | | | | | | | |  | | | | | | | | |  | | | | | | | | | | |  | | | | | | | | | |  | | | | | | | | | | |  | | | | | | | | | |  | | | | | | | | |  | | | | | | | |
| Italy | 0 | | | | | | | 5 (41.7) | | | | | | | 6 (50.0) | | | | | | | 1 (8.3) | | | | | | 12 | | | | | |  | | | | | | | | | | | 0 | | | | | | | | | | 4 (100.0) | | | | | | | | | | 0 | | | | | | | | | 0 | | | | | | | | | | 4 | | | | | | | | | | | | | |  | | | | | | | | | 0 | | | | | | | | | | | 4 (21.1) | | | | | | | | | | 5 (26.3) | | | | | | | | | | | 8 (42.1) | | | | | | | | | | 2 (10.5) | | | | | | | | | 19 | | | | | | | |
|  |  | | | | | | |  | | | | | | |  | | | | | | |  | | | | | |  | | | | | |  | | | | | | | | | | |  | | | | | | | | | |  | | | | | | | | | |  | | | | | | | | |  | | | | | | | | | |  | | | | | | | | | | | | | |  | | | | | | | | |  | | | | | | | | | | |  | | | | | | | | | |  | | | | | | | | | | |  | | | | | | | | | |  | | | | | | | | |  | | | | | | | |
| Poland | 0 | | | | | | | 6 (27.3) | | | | | | | 16 (72.7) | | | | | | | 0 | | | | | | 22 | | | | | |  | | | | | | | | | | | 0 | | | | | | | | | | 0 | | | | | | | | | | 1 (100.0) | | | | | | | | | 0 | | | | | | | | | | 1 | | | | | | | | | | | | | |  | | | | | | | | | 1 (12.5) | | | | | | | | | | | 3 (37.5) | | | | | | | | | | 2 (25.0) | | | | | | | | | | | 2 (25.0) | | | | | | | | | | 0 | | | | | | | | | 8 | | | | | | | |
|  |  | | | | | | |  | | | | | | |  | | | | | | |  | | | | | |  | | | | | |  | | | | | | | | | | |  | | | | | | | | | |  | | | | | | | | | |  | | | | | | | | |  | | | | | | | | | |  | | | | | | | | | | | | | |  | | | | | | | | |  | | | | | | | | | | |  | | | | | | | | | |  | | | | | | | | | | |  | | | | | | | | | |  | | | | | | | | |  | | | | | | | |
| Iceland | 0 | | | | | | | 4 (30.8) | | | | | | | 9 (69.2) | | | | | | | 0 | | | | | | 13 | | | | | |  | | | | | | | | | | | 0 | | | | | | | | | | 1 (33.3) | | | | | | | | | | 2 (66.6) | | | | | | | | | 0 | | | | | | | | | | 3 | | | | | | | | | | | | | |  | | | | | | | | | 0 | | | | | | | | | | | 0 | | | | | | | | | | 0 | | | | | | | | | | | 2 (50.0) | | | | | | | | | | 2 (50.0) | | | | | | | | | 4 | | | | | | | |
|  |  | | | | | | |  | | | | | | |  | | | | | | |  | | | | | |  | | | | | |  | | | | | | | | | | |  | | | | | | | | | |  | | | | | | | | | |  | | | | | | | | |  | | | | | | | | | |  | | | | | | | | | | | | | |  | | | | | | | | |  | | | | | | | | | | |  | | | | | | | | | |  | | | | | | | | | | |  | | | | | | | | | |  | | | | | | | | |  | | | | | | | |
| United Kingdom | 1 (5.0) | | | | | | | 10 (50.0) | | | | | | | 7 (35.0) | | | | | | | 2 (10.0) | | | | | | 20 | | | | | |  | | | | | | | | | | | 0 | | | | | | | | | | 0 | | | | | | | | | | 0 | | | | | | | | | 0 | | | | | | | | | | 0 | | | | | | | | | | | | | |  | | | | | | | | | 3 (50.0) | | | | | | | | | | | 0 | | | | | | | | | | 2 (33.3) | | | | | | | | | | | 1 (16.7) | | | | | | | | | | 0 | | | | | | | | | 6 | | | | | | | |
|  |  | | | | | |  | | | | | | |  | | | | | |  | | | | | | |  | | | | | |  | | | | | | | | | | |  | | | | | | | | | |  | | | | | | | | | |  | | | | | | | | |  | | | | | | | | | |  | | | | | | | | | | | | |  | | | | | | | | | |  | | | | | | | | | | |  | | | | | | | | | |  | | | | | | | | | | |  | | | | | | | | | |  | | | | | | | | |  | | | | | | | | |
| Republic of Ireland | 1 (11.1) | | | | | | 6 (66.7) | | | | | | | 1 (11.1) | | | | | | 1 (11.1) | | | | | | | 9 | | | | | |  | | | | | | | | | | | 1 (25.0) | | | | | | | | | | 3 (75.0) | | | | | | | | | | 0 | | | | | | | | | 0 | | | | | | | | | | 4 | | | | | | | | | | | | |  | | | | | | | | | | 0 | | | | | | | | | | | 0 | | | | | | | | | | 0 | | | | | | | | | | | 0 | | | | | | | | | | 0 | | | | | | | | | 0 | | | | | | | | |
|  |  | | | | | |  | | | | | | |  | | | | | |  | | | | | | |  | | | | | |  | | | | | | | | | | |  | | | | | | | | | |  | | | | | | | | | |  | | | | | | | | |  | | | | | | | | | |  | | | | | | | | | | | | |  | | | | | | | | | |  | | | | | | | | | | |  | | | | | | | | | |  | | | | | | | | | | |  | | | | | | | | | |  | | | | | | | | |  | | | | | | | | |
| German | 0 | | | | | | 0 | | | | | | | 4 (80.0) | | | | | | 1 (20.0) | | | | | | | 5 | | | | | |  | | | | | | | | | | | 0 | | | | | | | | | | 0 | | | | | | | | | | 0 | | | | | | | | | 0 | | | | | | | | | | 0 | | | | | | | | | | | | |  | | | | | | | | | | 0 | | | | | | | | | | | 0 | | | | | | | | | | 0 | | | | | | | | | | | 0 | | | | | | | | | | 0 | | | | | | | | | 0 | | | | | | | | |
| Portugal | 0 | | | | | | 0 | | | | | | | 0 | | | | | | 0 | | | | | | | 0 | | | | | |  | | | | | | | | | | | 0 | | | | | | | | | | 0 | | | | | | | | | | 0 | | | | | | | | | 0 | | | | | | | | | | 0 | | | | | | | | | | | | |  | | | | | | | | | | 0 | | | | | | | | | | | 1 (50.0) | | | | | | | | | | 0 | | | | | | | | | | | 1 (50.0) | | | | | | | | | | 0 | | | | | | | | | 2 | | | | | | | | |
|  | | |  | | | | | | | | |  | | | | | |  | | | | | |  | | |  | | | | | | | | |  | | | | |  | | | | | | | | | |  | | | | | | | | | |  | | | | | | | | |  | | | | | | | | | |  | | | | | | | | | |  | | |  | | | | | | | | | | | |  | | | | | | | | | | |  | | | | | | | | | | | | | | | | | | |  | | | | | | | | | |  | | | | | | | | | | | | | | | | | | | |  |
| **Referral for specialist care for health or medical problems** | | | | | | | | | | | | | | | | | | | | | | | | | | | | | | | | | | | | | | | | | | | | | | | | | | | | | | | | | | | | | | | | | | | | | | | | | | | | | | | | | | | | | | | | | | | | | | | | | | | | | | | | | | | | | | | | | | | | | | | | | | | | | | | | | | | | | | | | | | | | | | | | | | | | | | | | | | | | | | | | | | | | | |
| Denmark | 17 (11.0) | | | | | | 85 (55.2) | | | | | | | 43 (27.9) | | | | | | 9 (5.8) | | | | | | | 154 | | | | |  | | | | | | | | | | | 6 (18.7) | | | | | | | | | | 18 (56.3) | | | | | | | | | | 7 (21.9) | | | | | | | | | 1 (3.1) | | | | | | | | | | 32 | | | | | | | | | | | | |  | | | | | | | | | | | 5 (23.8) | | | | | | | | | | | 5 (23.8) | | | | | | | | | | 1 (4.8) | | | | | | | | | | | 1 (4.8) | | | | | | | | | | 9 (42.9) | | | | | | | | | 21 | | | | | | | | |
|  |  | | | | | |  | | | | | | |  | | | | | |  | | | | | | |  | | | | |  | | | | | | | | | | |  | | | | | | | | | |  | | | | | | | | | |  | | | | | | | | |  | | | | | | | | | |  | | | | | | | | | | | | |  | | | | | | | | | | |  | | | | | | | | | | |  | | | | | | | | | |  | | | | | | | | | | |  | | | | | | | | | |  | | | | | | | | |  | | | | | | | | |
| Finland | 8 (16.7) | | | | | | 29 (60.4) | | | | | | | 10 (20.8) | | | | | | 1 (2.1) | | | | | | | 48 | | | | |  | | | | | | | | | | | 1 (25.0) | | | | | | | | | | 2 (50.0) | | | | | | | | | | 0 | | | | | | | | | 1 (25.0) | | | | | | | | | | 4 | | | | | | | | | | | | |  | | | | | | | | | | | 15 (38.5) | | | | | | | | | | | 10 (25.6) | | | | | | | | | | 8 (20.5) | | | | | | | | | | | 0 | | | | | | | | | | 6 (15.4) | | | | | | | | | 39 | | | | | | | | |
|  |  | | | | | |  | | | | | | |  | | | | | |  | | | | | | |  | | | | |  | | | | | | | | | | |  | | | | | | | | | |  | | | | | | | | | |  | | | | | | | | |  | | | | | | | | | |  | | | | | | | | | | | | |  | | | | | | | | | | |  | | | | | | | | | | |  | | | | | | | | | |  | | | | | | | | | | |  | | | | | | | | | |  | | | | | | | | |  | | | | | | | | |
| France | 8 (17.8) | | | | | | 20 (44.4) | | | | | | | 13 (28.9) | | | | | | 4 (8.9) | | | | | | | 45 | | | | |  | | | | | | | | | | | 4 (33.3) | | | | | | | | | | 7 (58.3) | | | | | | | | | | 1 (8.3) | | | | | | | | | 0 | | | | | | | | | | 12 | | | | | | | | | | | | |  | | | | | | | | | | | 13 (48.2) | | | | | | | | | | | 6 (22.2) | | | | | | | | | | 2 (7.4) | | | | | | | | | | | 2 (7.4) | | | | | | | | | | 4 (14.8) | | | | | | | | | 27 | | | | | | | | |
|  |  | | | | | |  | | | | | | |  | | | | | |  | | | | | | |  | | | | |  | | | | | | | | | | |  | | | | | | | | | |  | | | | | | | | | |  | | | | | | | | |  | | | | | | | | | |  | | | | | | | | | | | | |  | | | | | | | | | | |  | | | | | | | | | | |  | | | | | | | | | |  | | | | | | | | | | |  | | | | | | | | | |  | | | | | | | | |  | | | | | | | | |
| Spain | 2 (12.5) | | | | | | 6 (37.5) | | | | | | | 7 (43.8) | | | | | | 1 (6.2) | | | | | | | 16 | | | | |  | | | | | | | | | | | 3 (75.0) | | | | | | | | | | 0 | | | | | | | | | | 1 (25.0) | | | | | | | | | 0 | | | | | | | | | | 4 | | | | | | | | | | | | |  | | | | | | | | | | | 6 (46.2) | | | | | | | | | | | 6 (46.2) | | | | | | | | | | 1 (7.7) | | | | | | | | | | | 0 | | | | | | | | | | 0 | | | | | | | | | 13 | | | | | | | | |
|  |  | | | | | |  | | | | | | |  | | | | | |  | | | | | | |  | | | | |  | | | | | | | | | | |  | | | | | | | | | |  | | | | | | | | | |  | | | | | | | | |  | | | | | | | | | |  | | | | | | | | | | | | |  | | | | | | | | | | |  | | | | | | | | | | |  | | | | | | | | | |  | | | | | | | | | | |  | | | | | | | | | |  | | | | | | | | |  | | | | | | | | |
| Italy | 0 | | | | | | 3 (25.0) | | | | | | | 8 (66.7) | | | | | | 1 (8.3) | | | | | | | 12 | | | | |  | | | | | | | | | | | 2 (50.0) | | | | | | | | | | 1 (25.0) | | | | | | | | | | 1 (25.0) | | | | | | | | | 0 | | | | | | | | | | 4 | | | | | | | | | | | | |  | | | | | | | | | | | 9 (47.4) | | | | | | | | | | | 7 (36.8) | | | | | | | | | | 2 (10.5) | | | | | | | | | | | 0 | | | | | | | | | | 1 (5.3) | | | | | | | | | 19 | | | | | | | | |
|  |  | | | | | |  | | | | | | |  | | | | | |  | | | | | | |  | | | | |  | | | | | | | | | | |  | | | | | | | | | |  | | | | | | | | | |  | | | | | | | | |  | | | | | | | | | |  | | | | | | | | | | | | |  | | | | | | | | | | |  | | | | | | | | | | |  | | | | | | | | | |  | | | | | | | | | | |  | | | | | | | | | |  | | | | | | | | |  | | | | | | | | |
| Poland | 3 (13.6) | | | | | | 7 (31.8) | | | | | | | 11 (50.0) | | | | | | 1 (4.6) | | | | | | | 22 | | | | |  | | | | | | | | | | | 1 (100.0) | | | | | | | | | | 0 | | | | | | | | | | 0 | | | | | | | | | 0 | | | | | | | | | | 1 | | | | | | | | | | | | |  | | | | | | | | | | | 2 (25.0) | | | | | | | | | | | 4 (50.0) | | | | | | | | | | 2 (25.0) | | | | | | | | | | | 0 | | | | | | | | | | 0 | | | | | | | | | 8 | | | | | | | | |
|  |  | | | | | |  | | | | | | |  | | | | | |  | | | | | | |  | | | | |  | | | | | | | | | | |  | | | | | | | | | |  | | | | | | | | | |  | | | | | | | | |  | | | | | | | | | |  | | | | | | | | | | | | |  | | | | | | | | | | |  | | | | | | | | | | |  | | | | | | | | | |  | | | | | | | | | | |  | | | | | | | | | |  | | | | | | | | |  | | | | | | | | |
| Iceland | 0 | | | | | | 5 (38.5) | | | | | | | 5 (38.5) | | | | | | 3 (23.1) | | | | | | | 13 | | | | |  | | | | | | | | | | | 2 (66.7) | | | | | | | | | | 1 (33.3) | | | | | | | | | | 0 | | | | | | | | | 0 | | | | | | | | | | 3 | | | | | | | | | | | | |  | | | | | | | | | | | 0 | | | | | | | | | | | 2 (50.0) | | | | | | | | | | 0 | | | | | | | | | | | 0 | | | | | | | | | | 2 (50.0) | | | | | | | | | 4 | | | | | | | | |
|  |  | | | | | |  | | | | | | |  | | | | | |  | | | | | | |  | | | | |  | | | | | | | | | | |  | | | | | | | | | |  | | | | | | | | | |  | | | | | | | | |  | | | | | | | | | |  | | | | | | | | | | | | |  | | | | | | | | | | |  | | | | | | | | | | |  | | | | | | | | | |  | | | | | | | | | | |  | | | | | | | | | |  | | | | | | | | |  | | | | | | | | |
| United Kingdom | 1 (5.0) | | | | | | 11 (55.0) | | | | | | | 7 (35.0) | | | | | | 1 (5.0) | | | | | | | 20 | | | | |  | | | | | | | | | | | 0 | | | | | | | | | | 0 | | | | | | | | | | 0 | | | | | | | | | 0 | | | | | | | | | | 0 | | | | | | | | | | | | |  | | | | | | | | | | | 5 (83.3) | | | | | | | | | | | 1 (16.7) | | | | | | | | | | 0 | | | | | | | | | | | 0 | | | | | | | | | | 0 | | | | | | | | | 6 | | | | | | | | |
|  |  | | | | | |  | | | | | | |  | | | | | |  | | | | | | |  | | | | |  | | | | | | | | | | |  | | | | | | | | | |  | | | | | | | | | |  | | | | | | | | |  | | | | | | | | | |  | | | | | | | | | | | | |  | | | | | | | | | | |  | | | | | | | | | | |  | | | | | | | | | |  | | | | | | | | | | |  | | | | | | | | | |  | | | | | | | | |  | | | | | | | | |
| Republic of Ireland | 1 (11.1) | | | | | | 4 (44.4) | | | | | | | 2 (22.2) | | | | | | 2 (22.2) | | | | | | | 9 | | | | |  | | | | | | | | | | | 1 (25.0) | | | | | | | | | | 3 (75.0) | | | | | | | | | | 0 | | | | | | | | | 0 | | | | | | | | | | 4 | | | | | | | | | | | | |  | | | | | | | | | | | 0 | | | | | | | | | | | 0 | | | | | | | | | | 0 | | | | | | | | | | | 0 | | | | | | | | | | 0 | | | | | | | | | 0 | | | | | | | | |
|  |  | | | | | |  | | | | | | |  | | | | | |  | | | | | | |  | | | | |  | | | | | | | | | | |  | | | | | | | | | |  | | | | | | | | | |  | | | | | | | | |  | | | | | | | | | |  | | | | | | | | | | | | |  | | | | | | | | | | |  | | | | | | | | | | |  | | | | | | | | | |  | | | | | | | | | | |  | | | | | | | | | |  | | | | | | | | |  | | | | | | | | |
| German | 0 | | | | | | 1 (20.0) | | | | | | | 3 (60.0) | | | | | | 1 (20.0) | | | | | | | 5 | | | | |  | | | | | | | | | | | 0 | | | | | | | | | | 0 | | | | | | | | | | 0 | | | | | | | | | 0 | | | | | | | | | | 0 | | | | | | | | | | | | |  | | | | | | | | | | | 0 | | | | | | | | | | | 0 | | | | | | | | | | 0 | | | | | | | | | | | 0 | | | | | | | | | | 0 | | | | | | | | | 0 | | | | | | | | |
| Portugal | 0 | | | | | | 0 | | | | | | | 0 | | | | | | 0 | | | | | | | 0 | | | | |  | | | | | | | | | | | 0 | | | | | | | | | | 0 | | | | | | | | | | 0 | | | | | | | | | 0 | | | | | | | | | | 0 | | | | | | | | | | | | |  | | | | | | | | | | | 2 (100.0) | | | | | | | | | | | 0 | | | | | | | | | | 0 | | | | | | | | | | | 0 | | | | | | | | | | 0 | | | | | | | | | 2 | | | | | | | | |
|  | | |  | | | | | | | | |  | | | | | |  | | | | | |  | | |  | | | | | | | | |  | | | | |  | | | | | | | | | |  | | | | | | | | | |  | | | | | | | | |  | | | | | | | | | |  | | | | | | | | | |  | | |  | | | | | | | | | | | |  | | | | | | | | | | |  | | | | | | | | | | | | | | | | | | |  | | | | | | | | | |  | | | | | | | | | | | | | | | | | | | |  |
| **Written recommendations for how to manage a crisis** | | | | | | | | | | | | | | | | | | | | | | | | | | | | | | | | | | | | | | | | | | | | | | | | | | | | | | | | | | | | | | | | | | | | | | | | | | | | | | | | | | | | | | | | | | | | | | | | | | | | | | | | | | | | | | | | | | | | | | | | | | | | | | | | | | | | | | | | | | | | | | | | | | | | | | | | | | | | | | | | | | | | | |
| Denmark | | | | N/A | | | | | | | | | | | | | | | | | | | | | | | | | | | | | | | | |  | | | | | | | | | | 0 | | | | | | | | | | 23 (71.9) | | | | | | | | | | 6 (18.7) | | | | | | | | | 3 (9.4) | | | | | | | | | | 32 | | | | | | | | | | | | | |  | | | | | | | | | 4 (19.1) | | | | | | | | | | | 5 (23.8) | | | | | | | | | | 1 (4.8) | | | | | | | | | | 0 | | | | | | | | | | 11 (52.4) | | | | | | | | | 21 | | | | | | |
|  | | | |  | | | | | | | | | | | | | | | | | | | | | | | | | | | | | | | | |  | | | | | | | | | |  | | | | | | | | | |  | | | | | | | | | |  | | | | | | | | |  | | | | | | | | | |  | | | | | | | | | | | | | |  | | | | | | | | |  | | | | | | | | | | |  | | | | | | | | | |  | | | | | | | | | |  | | | | | | | | | |  | | | | | | | | |  | | | | | | |
| Finland | | | | N/A | | | | | | | | | | | | | | | | | | | | | | | | | | | | | | | | |  | | | | | | | | | | 0 | | | | | | | | | | 1 (25.0) | | | | | | | | | | 0 | | | | | | | | | 3 (75.0) | | | | | | | | | | 4 | | | | | | | | | | | | | |  | | | | | | | | | 4 (10.3) | | | | | | | | | | | 8 (20.6) | | | | | | | | | | 12 (30.8) | | | | | | | | | | 2 (5.1) | | | | | | | | | | 13 (33.3) | | | | | | | | | 39 | | | | | | |
|  | | | |  | | | | | | | | | | | | | | | | | | | | | | | | | | | | | | | | |  | | | | | | | | | |  | | | | | | | | | |  | | | | | | | | | |  | | | | | | | | |  | | | | | | | | | |  | | | | | | | | | | | | | |  | | | | | | | | |  | | | | | | | | | | |  | | | | | | | | | |  | | | | | | | | | |  | | | | | | | | | |  | | | | | | | | |  | | | | | | |
| France | | | | N/A | | | | | | | | | | | | | | | | | | | | | | | | | | | | | | | | |  | | | | | | | | | | 4 (33.3) | | | | | | | | | | 5 (41.7) | | | | | | | | | | 3 (25.0) | | | | | | | | | 0 | | | | | | | | | | 12 | | | | | | | | | | | | | |  | | | | | | | | | 7 (25.9) | | | | | | | | | | | 8 (29.6) | | | | | | | | | | 5 (18.5) | | | | | | | | | | 1 (3.7) | | | | | | | | | | 6 (22.2) | | | | | | | | | 27 | | | | | | |
|  | | | |  | | | | | | | | | | | | | | | | | | | | | | | | | | | | | | | | |  | | | | | | | | | |  | | | | | | | | | |  | | | | | | | | | |  | | | | | | | | |  | | | | | | | | | |  | | | | | | | | | | | | | |  | | | | | | | | |  | | | | | | | | | | |  | | | | | | | | | |  | | | | | | | | | |  | | | | | | | | | |  | | | | | | | | |  | | | | | | |
| Spain | | | | N/A | | | | | | | | | | | | | | | | | | | | | | | | | | | | | | | | |  | | | | | | | | | | 1 (25.0) | | | | | | | | | | 2 (50.0) | | | | | | | | | | 1 (25.0) | | | | | | | | | 0 | | | | | | | | | | 4 | | | | | | | | | | | | | |  | | | | | | | | | 6 (46.2) | | | | | | | | | | | 4 (30.8) | | | | | | | | | | 3 (23.1) | | | | | | | | | | 0 | | | | | | | | | | 0 | | | | | | | | | 13 | | | | | | |
|  | | | |  | | | | | | | | | | | | | | | | | | | | | | | | | | | | | | | | |  | | | | | | | | | |  | | | | | | | | | |  | | | | | | | | | |  | | | | | | | | |  | | | | | | | | | |  | | | | | | | | | | | | | |  | | | | | | | | |  | | | | | | | | | | |  | | | | | | | | | |  | | | | | | | | | |  | | | | | | | | | |  | | | | | | | | |  | | | | | | |
| Italy | | | | N/A | | | | | | | | | | | | | | | | | | | | | | | | | | | | | | | | |  | | | | | | | | | | 0 | | | | | | | | | | 3 (75.0) | | | | | | | | | | 1 (25.0) | | | | | | | | | 0 | | | | | | | | | | 4 | | | | | | | | | | | | | |  | | | | | | | | | 11 (57.9) | | | | | | | | | | | 3 (15.8) | | | | | | | | | | 3 (15.8) | | | | | | | | | | 0 | | | | | | | | | | 2 (10.5) | | | | | | | | | 19 | | | | | | |
|  | | | |  | | | | | | | | | | | | | | | | | | | | | | | | | | | | | | | | |  | | | | | | | | | |  | | | | | | | | | |  | | | | | | | | | |  | | | | | | | | |  | | | | | | | | | |  | | | | | | | | | | | | | |  | | | | | | | | |  | | | | | | | | | | |  | | | | | | | | | |  | | | | | | | | | |  | | | | | | | | | |  | | | | | | | | |  | | | | | | |
| Poland | | | | N/A | | | | | | | | | | | | | | | | | | | | | | | | | | | | | | | | |  | | | | | | | | | | 0 | | | | | | | | | | 1 (100.0) | | | | | | | | | | 0 | | | | | | | | | 0 | | | | | | | | | | 1 | | | | | | | | | | | | | |  | | | | | | | | | 0 | | | | | | | | | | | 6 (75.0) | | | | | | | | | | 2 (25.0) | | | | | | | | | | 0 | | | | | | | | | | 0 | | | | | | | | | 8 | | | | | | |
|  | | | |  | | | | | | | | | | | | | | | | | | | | | | | | | | | | | | | | |  | | | | | | | | | |  | | | | | | | | | |  | | | | | | | | | |  | | | | | | | | |  | | | | | | | | | |  | | | | | | | | | | | | | |  | | | | | | | | |  | | | | | | | | | | |  | | | | | | | | | |  | | | | | | | | | |  | | | | | | | | | |  | | | | | | | | |  | | | | | | |
| Iceland | | | | N/A | | | | | | | | | | | | | | | | | | | | | | | | | | | | | | | | |  | | | | | | | | | | 0 | | | | | | | | | | 1 (33.3) | | | | | | | | | | 2 (66.7) | | | | | | | | | 0 | | | | | | | | | | 3 | | | | | | | | | | | | | |  | | | | | | | | | 0 | | | | | | | | | | | 0 | | | | | | | | | | 1 (25.0) | | | | | | | | | | 1 (25.0) | | | | | | | | | | 2 (50.0) | | | | | | | | | 4 | | | | | | |
|  | | | |  | | | | | | | | | | | | | | | | | | | | | | | | | | | | | | | | |  | | | | | | | | | |  | | | | | | | | | |  | | | | | | | | | |  | | | | | | | | |  | | | | | | | | | |  | | | | | | | | | | | | | |  | | | | | | | | |  | | | | | | | | | | |  | | | | | | | | | |  | | | | | | | | | |  | | | | | | | | | |  | | | | | | | | |  | | | | | | |
| United Kingdom | | | | N/A | | | | | | | | | | | | | | | | | | | | | | | | | | | | | | | | |  | | | | | | | | | | 0 | | | | | | | | | | 0 | | | | | | | | | | 0 | | | | | | | | | 0 | | | | | | | | | | 0 | | | | | | | | | | | | | |  | | | | | | | | | 2 (33.3) | | | | | | | | | | | 3 (50.0) | | | | | | | | | | 1 (16.7) | | | | | | | | | | 0 | | | | | | | | | | 0 | | | | | | | | | 6 | | | | | | |
|  | | | |  | | | | | | | | | | | | | | | | | | | | | | | | | | | | | | | | |  | | | | | | | | | |  | | | | | | | | | |  | | | | | | | | | |  | | | | | | | | |  | | | | | | | | | |  | | | | | | | | | | | | | |  | | | | | | | | |  | | | | | | | | | | |  | | | | | | | | | |  | | | | | | | | | |  | | | | | | | | | |  | | | | | | | | |  | | | | | | |
| Republic of Ireland | | | | N/A | | | | | | | | | | | | | | | | | | | | | | | | | | | | | | | | |  | | | | | | | | | | 0 | | | | | | | | | | 3 (75.0) | | | | | | | | | | 0 | | | | | | | | | 1 (25.0) | | | | | | | | | | 4 | | | | | | | | | | | | | |  | | | | | | | | | 0 | | | | | | | | | | | 0 | | | | | | | | | | 0 | | | | | | | | | | 0 | | | | | | | | | | 0 | | | | | | | | | 0 | | | | | | |
|  | | | |  | | | | | | | | | | | | | | | | | | | | | | | | | | | | | | | | |  | | | | | | | | | |  | | | | | | | | | |  | | | | | | | | | |  | | | | | | | | |  | | | | | | | | | |  | | | | | | | | | | | | | |  | | | | | | | | |  | | | | | | | | | | |  | | | | | | | | | |  | | | | | | | | | |  | | | | | | | | | |  | | | | | | | | |  | | | | | | |
| German | | | | N/A | | | | | | | | | | | | | | | | | | | | | | | | | | | | | | | | |  | | | | | | | | | | 0 | | | | | | | | | | 0 | | | | | | | | | | 0 | | | | | | | | | 0 | | | | | | | | | | 0 | | | | | | | | | | | | | |  | | | | | | | | | 0 | | | | | | | | | | | 0 | | | | | | | | | | 0 | | | | | | | | | | 0 | | | | | | | | | | 0 | | | | | | | | | 0 | | | | | | |
|  | | | |  | | | | | | | | | | | | | | | | | | | | | | | | | | | | | | | | |  | | | | | | | | | |  | | | | | | | | | |  | | | | | | | | | |  | | | | | | | | |  | | | | | | | | | |  | | | | | | | | | | | | | |  | | | | | | | | |  | | | | | | | | | | |  | | | | | | | | | |  | | | | | | | | | |  | | | | | | | | | |  | | | | | | | | |  | | | | | | |
| Portugal | | | | N/A | | | | | | | | | | | | | | | | | | | | | | | | | | | | | | | | |  | | | | | | | | | | 0 | | | | | | | | | | 0 | | | | | | | | | | 0 | | | | | | | | | 0 | | | | | | | | | | 0 | | | | | | | | | | | | | |  | | | | | | | | | 2 (100.0) | | | | | | | | | | | 0 | | | | | | | | | | 0 | | | | | | | | | | 0 | | | | | | | | | | 0 | | | | | | | | | 2 | | | | | | |
|  | | | | | |  | | | | | | |  | | | | | |  | | | | | |  | | | |  | | | | | | | | | |  | | |  | | | | | | | | | |  | | | | | | | | | |  | | | | | | | | |  | | | | | | | | | |  | | | | | | | | | | |  | | | | |  | | | | | | | | | | | | | | | | |  | | | | | | | | | | | |  | | | | | | | | | | |  | | | | | | | | | |  | | | | | | | | |  | | | | | | | | | |
| **Written recommendations for managing risks** | | | | | | | | | | | | | | | | | | | | | | | | | | | | | | | | | | | | | | | | | | | | | | | | | | | | | | | | | | | | | | | | | | | | | | | | | | | | | | | | | | | | | | | | | | |  | | |  | | | | | | | | | | | | | | | | | |  | | | | | | | | | | | | |  | | | | | | | | | | |  | | | | | | | | | |  | | | | | | | | |  | | | | | | | | | | |
| Denmark | | | | | | N/A | | | | | | | | | | | | | | | | | | | | | | | | | | | | | | | | |  | | | | | | | | | | 8 (6.2) | | | | | | | | | | 14 (43.8) | | | | | | | | | 7 (21.9) | | | | | | | | | | 9 (28.1) | | | | | | | | | | 32 | | | | | | | | | | | | | |  | | | | | | | | | 7 (33.3) | | | | | | | | | | | 2 (9.5) | | | | | | | | | | 1 (4.8) | | | | | | | | | | 0 | | | | | | | | | | 11 (52.4) | | | | | | | | | | 21 | | | |
|  | | | | | |  | | | | | | | | | | | | | | | | | | | | | | | | | | | | | | | | |  | | | | | | | | | |  | | | | | | | | | |  | | | | | | | | |  | | | | | | | | | |  | | | | | | | | | |  | | | | | | | | | | | | | |  | | | | | | | | |  | | | | | | | | | | |  | | | | | | | | | |  | | | | | | | | | |  | | | | | | | | | |  | | | | | | | | | |  | | | |
| Finland | | | | | | N/A | | | | | | | | | | | | | | | | | | | | | | | | | | | | | | | | |  | | | | | | | | | | 0 | | | | | | | | | | 3 (75.0) | | | | | | | | | 0 | | | | | | | | | | 1 (25.0) | | | | | | | | | | 4 | | | | | | | | | | | | | |  | | | | | | | | | 4 (10.3) | | | | | | | | | | | 8 (20.5) | | | | | | | | | | 9 (23.1) | | | | | | | | | | 2 (5.1) | | | | | | | | | | 16 (41.0) | | | | | | | | | | 39 | | | |
|  | | | | | |  | | | | | | | | | | | | | | | | | | | | | | | | | | | | | | | | |  | | | | | | | | | |  | | | | | | | | | |  | | | | | | | | |  | | | | | | | | | |  | | | | | | | | | |  | | | | | | | | | | | | | |  | | | | | | | | |  | | | | | | | | | | |  | | | | | | | | | |  | | | | | | | | | |  | | | | | | | | | |  | | | | | | | | | |  | | | |
| France | | | | | | N/A | | | | | | | | | | | | | | | | | | | | | | | | | | | | | | | | |  | | | | | | | | | | 0 | | | | | | | | | | 7 (58.3) | | | | | | | | | 4 (33.3) | | | | | | | | | | 1 (8.33) | | | | | | | | | | 12 | | | | | | | | | | | | | |  | | | | | | | | | 12 (44.4) | | | | | | | | | | | 7 (25.9) | | | | | | | | | | 3 (11.1) | | | | | | | | | | 0 | | | | | | | | | | 5 (18.5) | | | | | | | | | | 27 | | | |
|  | | | | | |  | | | | | | | | | | | | | | | | | | | | | | | | | | | | | | | | |  | | | | | | | | | |  | | | | | | | | | |  | | | | | | | | |  | | | | | | | | | |  | | | | | | | | | |  | | | | | | | | | | | | | |  | | | | | | | | |  | | | | | | | | | | |  | | | | | | | | | |  | | | | | | | | | |  | | | | | | | | | |  | | | | | | | | | |  | | | |
| Spain | | | | | | N/A | | | | | | | | | | | | | | | | | | | | | | | | | | | | | | | | |  | | | | | | | | | | 0 | | | | | | | | | | 1 (25.0) | | | | | | | | | 3 (75.0) | | | | | | | | | | 0 | | | | | | | | | | 4 | | | | | | | | | | | | | |  | | | | | | | | | 4 (30.8) | | | | | | | | | | | 6 (46.2) | | | | | | | | | | 3 (23.1) | | | | | | | | | | 0 | | | | | | | | | | 0 | | | | | | | | | | 13 | | | |
|  | | | | | |  | | | | | | | | | | | | | | | | | | | | | | | | | | | | | | | | |  | | | | | | | | | |  | | | | | | | | | |  | | | | | | | | |  | | | | | | | | | |  | | | | | | | | | |  | | | | | | | | | | | | | |  | | | | | | | | |  | | | | | | | | | | |  | | | | | | | | | |  | | | | | | | | | |  | | | | | | | | | |  | | | | | | | | | |  | | | |
| Italy | | | | | | N/A | | | | | | | | | | | | | | | | | | | | | | | | | | | | | | | | |  | | | | | | | | | | 0 | | | | | | | | | | 3 (75.0) | | | | | | | | | 1 (25.0) | | | | | | | | | | 0 | | | | | | | | | | 4 | | | | | | | | | | | | | |  | | | | | | | | | 8 (42.1) | | | | | | | | | | | 5 (26.3) | | | | | | | | | | 3 (17.8) | | | | | | | | | | 1 (5.3) | | | | | | | | | | 2 (10.5) | | | | | | | | | | 19 | | | |
|  | | | | | |  | | | | | | | | | | | | | | | | | | | | | | | | | | | | | | | | |  | | | | | | | | | |  | | | | | | | | | |  | | | | | | | | |  | | | | | | | | | |  | | | | | | | | | |  | | | | | | | | | | | | | |  | | | | | | | | |  | | | | | | | | | | |  | | | | | | | | | |  | | | | | | | | | |  | | | | | | | | | |  | | | | | | | | | |  | | | |
| Poland | | | | | | N/A | | | | | | | | | | | | | | | | | | | | | | | | | | | | | | | | |  | | | | | | | | | | 0 | | | | | | | | | | 0 | | | | | | | | | 1 (100.0) | | | | | | | | | | 0 | | | | | | | | | | 1 | | | | | | | | | | | | | |  | | | | | | | | | 2 (25.0) | | | | | | | | | | | 5 (62.5) | | | | | | | | | | 1 (12.5) | | | | | | | | | | 0 | | | | | | | | | | 0 | | | | | | | | | | 8 | | | |
|  | | | | | |  | | | | | | | | | | | | | | | | | | | | | | | | | | | | | | | | |  | | | | | | | | | |  | | | | | | | | | |  | | | | | | | | |  | | | | | | | | | |  | | | | | | | | | |  | | | | | | | | | | | | | |  | | | | | | | | |  | | | | | | | | | | |  | | | | | | | | | |  | | | | | | | | | |  | | | | | | | | | |  | | | | | | | | | |  | | | |
| Iceland | | | | | | N/A | | | | | | | | | | | | | | | | | | | | | | | | | | | | | | | | |  | | | | | | | | | | 0 | | | | | | | | | | 1 (33.3) | | | | | | | | | 2 (66.7) | | | | | | | | | | 0 | | | | | | | | | | 3 | | | | | | | | | | | | | |  | | | | | | | | | 0 | | | | | | | | | | | 0 | | | | | | | | | | 1 (25.0) | | | | | | | | | | 1 (25.0) | | | | | | | | | | 2 (50.0) | | | | | | | | | | 4 | | | |
|  | | | | | |  | | | | | | | | | | | | | | | | | | | | | | | | | | | | | | | | |  | | | | | | | | | |  | | | | | | | | | |  | | | | | | | | |  | | | | | | | | | |  | | | | | | | | | |  | | | | | | | | | | | | | |  | | | | | | | | |  | | | | | | | | | | |  | | | | | | | | | |  | | | | | | | | | |  | | | | | | | | | |  | | | | | | | | | |  | | | |
| United Kingdom | | | | | | N/A | | | | | | | | | | | | | | | | | | | | | | | | | | | | | | | | |  | | | | | | | | | | 0 | | | | | | | | | | 0 | | | | | | | | | 0 | | | | | | | | | | 0 | | | | | | | | | | 0 | | | | | | | | | | | | | |  | | | | | | | | | 3 (50.0) | | | | | | | | | | | 3 (50.0) | | | | | | | | | | 0 | | | | | | | | | | 0 | | | | | | | | | | 0 | | | | | | | | | | 6 | | | |
|  | | | | | |  | | | | | | | | | | | | | | | | | | | | | | | | | | | | | | | | |  | | | | | | | | | |  | | | | | | | | | |  | | | | | | | | |  | | | | | | | | | |  | | | | | | | | | |  | | | | | | | | | | | | | |  | | | | | | | | |  | | | | | | | | | | |  | | | | | | | | | |  | | | | | | | | | |  | | | | | | | | | |  | | | | | | | | | |  | | | |
| Republic of Ireland | | | | | | N/A | | | | | | | | | | | | | | | | | | | | | | | | | | | | | | | | |  | | | | | | | | | | 1 (25.0) | | | | | | | | | | 2 (50.0) | | | | | | | | | 0 | | | | | | | | | | 1 (25.0) | | | | | | | | | | 4 | | | | | | | | | | | | | |  | | | | | | | | | 0 | | | | | | | | | | | 0 | | | | | | | | | | 0 | | | | | | | | | | 0 | | | | | | | | | | 0 | | | | | | | | | | 0 | | | |
|  | | | | | |  | | | | | | | | | | | | | | | | | | | | | | | | | | | | | | | | |  | | | | | | | | | |  | | | | | | | | | |  | | | | | | | | |  | | | | | | | | | |  | | | | | | | | | |  | | | | | | | | | | | | | |  | | | | | | | | |  | | | | | | | | | | |  | | | | | | | | | |  | | | | | | | | | |  | | | | | | | | | |  | | | | | | | | | |  | | | |
| German | | | | | | N/A | | | | | | | | | | | | | | | | | | | | | | | | | | | | | | | | |  | | | | | | | | | | 0 | | | | | | | | | | 0 | | | | | | | | | 0 | | | | | | | | | | 0 | | | | | | | | | | 0 | | | | | | | | | | | | | |  | | | | | | | | | 0 | | | | | | | | | | | 0 | | | | | | | | | | 0 | | | | | | | | | | 0 | | | | | | | | | | 0 | | | | | | | | | | 0 | | | |
|  | | | | | |  | | | | | | | | | | | | | | | | | | | | | | | | | | | | | | | | |  | | | | | | | | | |  | | | | | | | | | |  | | | | | | | | |  | | | | | | | | | |  | | | | | | | | | |  | | | | | | | | | | | | | |  | | | | | | | | |  | | | | | | | | | | |  | | | | | | | | | |  | | | | | | | | | |  | | | | | | | | | |  | | | | | | | | | |  | | | |
| Portugal | | | | | | N/A | | | | | | | | | | | | | | | | | | | | | | | | | | | | | | | | |  | | | | | | | | | | 0 | | | | | | | | | | 0 | | | | | | | | | 0 | | | | | | | | | | 0 | | | | | | | | | | 0 | | | | | | | | | | | | | |  | | | | | | | | | 1 (50.0) | | | | | | | | | | | 1 (50.0) | | | | | | | | | | 0 | | | | | | | | | | 0 | | | | | | | | | | 0 | | | | | | | | | | 2 | | | |

**Supplementary material 14**

*Recommended features for autistic adult post-diagnosis support. Analysis removing adult and carer respondents who reported the adult’s autism diagnosis at a time before the 2012 publication of NICE guidelines.*

| Answer | Autistic adult (N=331) | | | |  | Carer (N=58) | | | |
| --- | --- | --- | --- | --- | --- | --- | --- | --- | --- |
|  | Yes | No, but it was needed | No and it was not needed | Do not know |  | Yes | No, but it was needed | No and it was not needed | Do not know |
| Written recommendations for care and follow-up for non-medical problems | 61 (18.4) | 175 (52.9) | 70 (21.2) | 25 (7.6) |  | 20 (34.5) | 32 (55.2) | 3 (5.2) | 3 (5.2) |
| Written recommendations for health care | 49 (14.8) | 168 (50.8) | 90 (27.2) | 24 (7.2) |  | 8 (13.8) | 34 (58.6) | 12 (20.7) | 4 (6.9) |
| ‘Health passport' to carry important information about the adult needs and care | 5 (1.5) | 197 (59.5) | 105 (31.7) | 24 (7.3) |  | 4 (6.9) | 41 (70.7) | 9 (15.5) | 4 (6.9) |
| Referral for specialist care for health or medical problems | 36  (10.9) | 168 (50.8) | 103 (31.1) | 24 (7.2) |  | 17 (29.3) | 30 (51.7) | 10 (17.2) | 1 (1.7) |
| Written recommendations for how to manage a crisis | N/A | | | |  | 5 (8.6) | 35 (60.3) | 13 (22.4) | 5 (8.6) |
| Written recommendations for managing risks | N/A | | | |  | 3 (5.2) | 28 (48.3) | 16 (27.6) | 11 (19.0) |

*Note*. Values expressed as number of responders and frequencies (in parenthesis). N/A=Question was not presented to the respondent group. The questions were the following: autistic adult=*After you got the autism spectrum diagnosis, which of the following things happened?*; Carer=*After the adult got the autism spectrum diagnosis, which of the following things happened?*; Professional=*Thinking of the adult diagnostic service for autism spectrum that you know best, how often are the following factors considered as parts of the post-diagnostic activities for autistic adults?*.
